# Biomarkers of mRNA vaccine efficacy derived from mechanistic modeling of tumor-immune interactions

**DOI:** 10.1371/journal.pcbi.1013163

**Published:** 2025-06-12

**Authors:** Chrysovalantis Voutouri, Lance L. Munn, Triantafyllos Stylianopoulos, Rakesh K. Jain

**Affiliations:** 1 Cancer Biophysics Laboratory, Department of Mechanical and Manufacturing Engineering, University of Cyprus, Nicosia, Cyprus; 2 Edwin L Steele Laboratories, Department of Radiation Oncology, Massachusetts General Hospital and Harvard Medical School, Boston, Massachusetts, United States of America; Northumbria University, UNITED KINGDOM OF GREAT BRITAIN AND NORTHERN IRELAND

## Abstract

The success of mRNA vaccines against infectious diseases such as COVID-19 has opened new avenues for their application in oncology. In cancer immunotherapy, mRNA vaccines—typically encapsulated in lipid nanoparticles (LNPs) 100–200 nm in size—enable delivery of tumor-specific antigens to activate immune responses. Here, we investigated the efficacy of mRNA vaccines in cancer by modeling tumor-immune interactions and tumor microenvironment (TME) dynamics to identify predictive biomarkers. Using a mechanistic mathematical model, we simulated tumor growth, immune cell dynamics, and vaccine pharmacokinetics in virtual cohorts of 1,635 patients generated via Latin hypercube sampling. Our simulations demonstrated a 45% average tumor size reduction and a 60% increase in CD8 + T cell infiltration in responsive tumors. Multiple regression analyses validated the predictive power of both pre- and on-treatment biomarkers. Key predictors of vaccine efficacy included antigen-presenting cell (APC) density and cytotoxic T cell fraction. Specifically, an APC density above 500 cells/mm³ in lymph nodes correlated with a 55% increase in vaccine response rates, while a cytotoxic T cell fraction above 20% in tumors was associated with a 60% reduction in tumor volume. A reduced M2/M1 macrophage ratio further improved treatment outcomes by 50%, highlighting the role of reprograming immunosuppressive macrophages. TME characteristics significantly influenced vaccine efficacy. Low extracellular matrix (ECM) density—modeled as a 5–10 × increase in hydraulic conductivity—combined with medium cytokine levels (IL-2 and TNF-α at 10–50 pg/ml), created optimal conditions for immune activation. Under these conditions, vaccine uptake improved by 35% and cytotoxic T cell infiltration increased by 65%, resulting in up to a 50% improvement in therapeutic outcomes. Model predictions aligned with pre-clinical data from melanoma and breast cancer models. These findings provide a framework for optimizing mRNA vaccine strategies and advancing personalized cancer immunotherapy.

## Author summary

Messenger RNA (mRNA) vaccines have shown remarkable success in preventing infectious diseases like COVID-19. Their potential in treating cancer is now gaining attention, especially for activating the immune system to recognize and destroy tumor cells. However, cancer cells grow in complex environments that can block immune responses and limit the effectiveness of these vaccines. In this study, we developed a detailed computer model that mimics how cancer cells and the immune system interact, including the structure of tumors and how immune cells behave. Using this model, we tested different treatment strategies in over 1,600 virtual patients to find the best conditions for mRNA vaccines to work. We discovered specific markers—such as immune cell levels and tumor structure—that can predict whether a patient will respond well to treatment. Our findings also show that reprogramming the tumor microenvironment and timing of treatment can significantly boost vaccine effectiveness. This research provides a roadmap for improving cancer immunotherapy and tailoring treatments to each patient, bringing us closer to personalized and more successful cancer care.

## Introduction

The promise of mRNA vaccines in oncology builds upon the success of COVID-19 mRNA vaccines, such as Moderna’s mRNA-1273 and Pfizer-BioNTech’s BNT162b2, which revolutionized the treatment of infectious diseases [1,2]. These vaccines, encapsulated within lipid nanoparticles (LNPs), protect mRNA from degradation and facilitate its delivery into cells [3–7]. In cancer treatment, mRNA vaccines introduce instructions for cells to produce tumor-specific antigens, thereby priming the immune system to recognize and attack cancer cells [3]. This process leverages the Cancer-Immunity cycle, a critical framework for understanding the interactions between cancer and the immune system [8]. However, the tumor microenvironment (TME) presents significant challenges to the efficacy of mRNA vaccines [9–13]. Tumor heterogeneity [14], both within and between different tumor types, complicates therapeutic interventions. The dense and stiff TME, characterized by compressed blood vessels, hypo-perfusion and hypoxia, impairs immune cell infiltration and function [14–19]. These conditions create barriers to the effective delivery and efficacy of mRNA vaccines [3,20,21]. Given these challenges, there is a pressing need to develop biomarkers predictive of response and optimize mRNA vaccine schedules and combinations with other therapies to enhance their effectiveness [22].

Computational modeling offers a useful tool to explore the complex interactions within the TME and the immune response to mRNA vaccines [23] Mechanistic mathematical models can simulate the dynamics of tumor growth, immune responses, and mRNA vaccine interactions, providing insights into optimal vaccination strategies [24–29]. Our mechanistic model incorporates multiple tumor-immune interactions, tumor microenvironmental factors, and pharmacokinetics/pharmacodynamics of mRNA vaccines, offering a comprehensive framework for predicting therapeutic outcomes. Simpler models often lack the capacity to simulate the complex dynamics of tumor stiffness, hydraulic conductivity, and cytokine-driven immune responses. For example, by integrating spatial and temporal heterogeneity within the TME, our model captures phenomena such as immune cell infiltration under varied ECM densities and cytokine production, which are crucial for understanding therapy efficacy. This complexity enables predictions of synergistic effects between mRNA vaccines and immune checkpoint inhibitors, which would not be possible with oversimplified frameworks. Such insights are vital for optimizing cancer immunotherapy strategies.

The novelty of our study lies in the integration of TME characteristics—such as ECM density, cytokine production, and immune cell infiltration dynamics—into a mechanistic mathematical model that predicts mRNA vaccine efficacy. Unlike traditional models [24–29], our approach incorporates both spatial and temporal heterogeneity of the TME to identify key biomarkers and optimize therapeutic strategies. This provides new insights into how microenvironmental factors influence vaccine uptake, immune activation, and overall treatment outcomes, advancing the field of personalized cancer immunotherapy.

In this study, we combine our mathematical frameworks for mechanistic modeling of COVID-19 mRNA vaccines and tumor-immune interactions [28,30–35] in order to investigate biological events and parameters that determine the vaccine efficacy in cancer therapy. These models incorporate principles from systems biology to simulate the intricate interplay between cancer cells, immune cells, and the TME. We further include detailed characteristics of the TME, such as increased tumor stiffness and vascular compression/hypo-perfusion, as well as the pharmacokinetics of vaccines and immune response dynamics. We modeled these factors and integrated pre-clinical data into the model, with the aim of identifying optimal mRNA vaccination schedules that can overcome the barriers posed by TME against immune response. Statistical analysis of the simulations’ results reveals biomarkers reflecting the effectiveness of vaccination.

## Methods

### Model description

**Fig 1:**
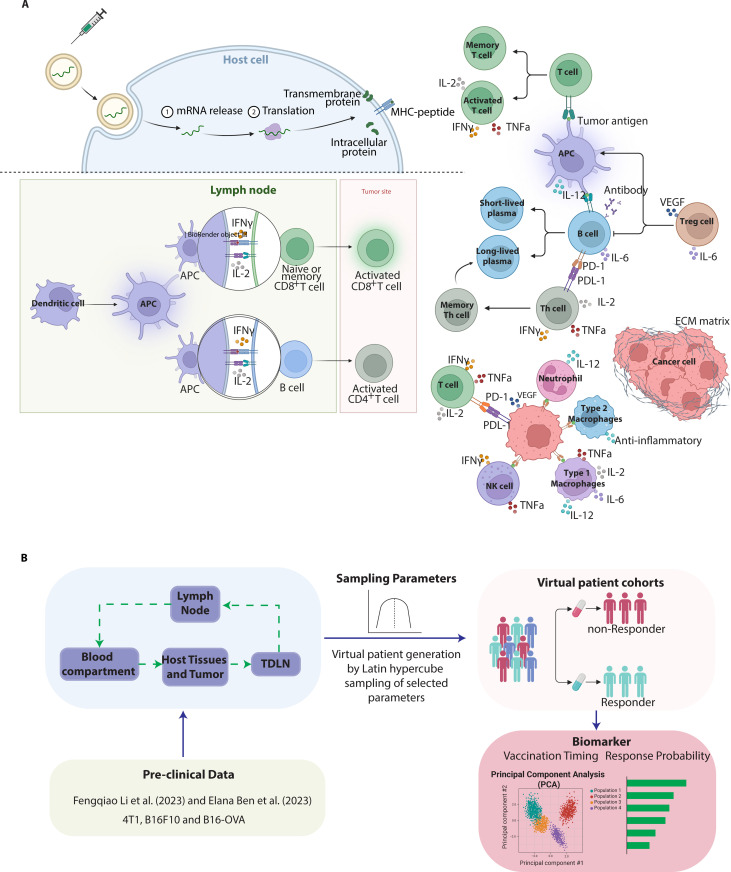
Biological Interplay and Pathways Incorporated in the Mathematical Model. (A) The schematic highlights the interactions between tumor cells and immune cells, showing key biological processes incorporated into the mathematical model. It focuses on tumor cell proliferation, influenced by oxygen levels in the tissue, and the death of tumor cells by immune cells, such as effector CD8 + T cells, natural killer cells, type 1 macrophages, and neutrophils. The figure also illustrates the activation of immune pathways by tumor antigens, immune checkpoints, and the detailed steps of vaccination-induced immunity, such as the generation of antigen-presenting cells, CD4+ and CD8 + effector T cells, memory T cells, and plasma B cells. (B) The model structure consists of four compartments: the tumor and surrounding host tissue, the tumor-draining lymph node (TDLN), the blood circulation, and lymph nodes. These compartments are used to simulate interactions between cancer and the immune system. The model integrates existing data and employs Latin hypercube sampling to generate virtual patient cohorts, with biomarker validation performed through feature selection and statistical analyses. Created with BioRender.com.

We developed a mechanistic model (Fig 1A) to provide a comprehensive analysis of tumor-immune cell interactions and TME to simulate mRNA vaccine uptake and effectiveness. Our model includes detailed representations of the following processes:

- Tumor Growth: The model captures the dynamics of tumor growth, considering the interactions among cancer cells, stromal cells, and the immune system. It integrates factors such as tissue stiffness, hypoxia, and vessel functionality to simulate the TME’s impact on cell proliferation and therapeutic efficacy.
- Immune Cell Dynamics: The model simulates the recruitment, activation, and suppression of various immune cells within the TME. It includes detailed equations for pro-inflammatory and anti-inflammatory cytokines, neutrophils, dendritic cells, T cells (CD4+ and CD8+), B cells, macrophages, and natural killer cells.
- Pharmacokinetics (PK): The PK model simulates vaccine transport and immune response dynamics within the TME. mRNA vaccines were encapsulated in LNPs approximately 100–200 nm diameter, designed to protect the mRNA from enzymatic degradation and enhance its stability in circulation. These LNPs facilitate efficient cellular uptake through endocytosis, ensuring effective delivery of the mRNA to antigen-presenting cells. While the model accounts for vaccine pharmacokinetics, including uptake and immune activation dynamics, it does not explicitly incorporate LNP particle size as a parameter. Instead, size-related effects are indirectly captured through uptake rate constants and biodistribution modeling. In the model, the LNPs were administered via intravenous (IV) injection, and their biodistribution was incorporated into the model using pharmacokinetic parameters to simulate their transport and uptake in different tissues, including the tumor microenvironment. Uptake rate constants were assigned based on values reported in the literature and adjusted during model calibration to reproduce observed tumor size and immune cell infiltration dynamics. These parameters fall within physiologically reasonable ranges observed in prior studies of lipid nanoparticle-mediated mRNA delivery.
- Modeling Immune Checkpoint Inhibitors (ICIs): We modeled the action of ICIs by incorporating their effects on immune checkpoint pathways, specifically the PD-1/PD-L1 axis. Vaccination protocols were simulated by administering mRNA vaccines on different initiation days, with doses adjusted based on pre-clinical data to ensure consistency with experimental designs. Simulations were run for 30 days to capture both short- and long-term effects of these therapies. Parameters were calibrated using control (PBS-treated) groups to ensure baseline accuracy before introducing treatment-specific modifications. The ICI dosing schedule (Days 8, 11, 14, 17) was applied uniformly across all simulations to ensure consistent comparison of immune and other TME effects.
- A representation of the simulation workflow is provided in Fig A in S1 Text, outlining the step-by-step process from initial parameter selection and model setup to simulation execution, data analysis, and validation with experimental data.

The analysis was initiated on Day 7, as preclinical data suggest that this time point aligns with the emergence of measurable immune responses and early tumor progression, providing a meaningful window to evaluate treatment effects [36,37]. Key time points (Days 8, 11, 14, and 17) were selected to capture distinct phases of the immune response and tumor growth dynamics. A single dose of the mRNA vaccine was administered to reflect its potent immunogenic effect observed in experimental models, whereas multiple doses of ICI were included to mimic clinical practices where continuous immune modulation is necessary.

The equations and parameters used in the model are included in the S1 Text, including production and degradation rates of cytokines, immune cell activation rates, and vaccine particle dynamics.

### Biomarker identification

We identified biomarkers and validated them using our model to predict mRNA vaccine efficacy, leading to a reduction in tumor growth. We identified both pre-treatment and on-treatment biomarkers.

### Virtual patient cohort simulations

We generated virtual patient cohorts (Fig 1B) using Latin hypercube sampling to ensure a representative distribution of physiological conditions and tumor heterogeneity [24–26,38,39]. This method allowed us to simulate 1,635 virtual patients, balancing computational feasibility with statistical robustness. The cohort size was chosen to encompass a diverse range of TME characteristics, ensuring that model predictions were representative of real-world variability. Smaller cohort sizes or random sampling could risk underrepresenting critical subpopulations, whereas the chosen size allowed for robust predictions and meaningful insights into biomarker efficacy.

### Statistical analysis

We performed statistical analyses, including principal components analysis (PCA) and non-negative matrix factorization (NMF), were performed to quantify the predictive accuracy of biomarkers and vaccination strategies. Confidence intervals, p-values, and effect sizes were calculated to support the validity of the findings. Subgroup analyses evaluated the efficacy of mRNA vaccines across different tumor types. In this analysis, we distinguished between two treatment scoring methods. The treatment score used in the PCA is based on the Euclidean distance, which quantifies the trajectory of immune response dynamics relative to a baseline (no-tumor) state. This metric captures the overall shift in immune-related parameters. In contrast, the treatment score used for tumor response evaluation is based on the RECIST criteria [40], which focuses on changes in tumor volume, with a reduction greater than 30% indicating a positive response.

### Model validation

**Fig 2:**
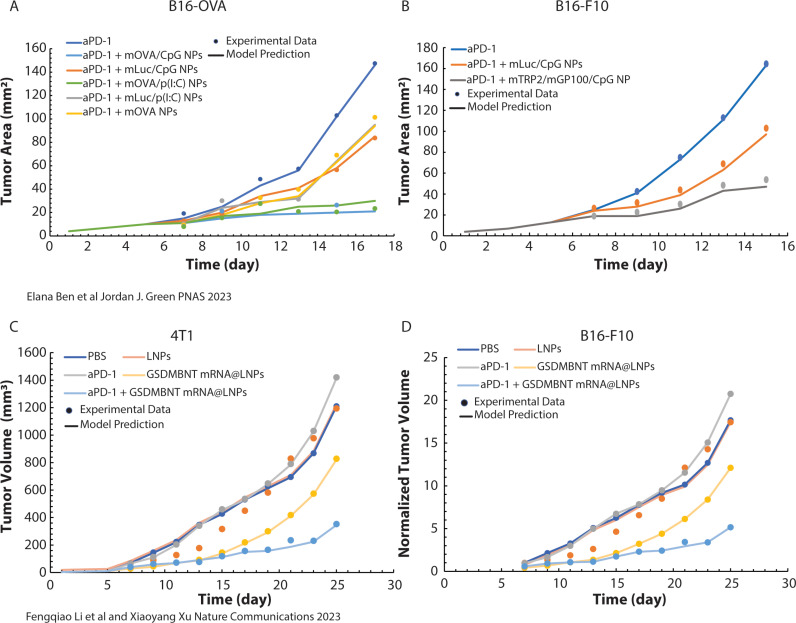
Comparison of Mechanistic Model Predictions with Experimental Data. Comparison of our mechanistic model predictions with experimental findings from two independent studies. (A-B) Correlation of model predictions with experimental results from Elana Ben et al. [36] (2023), showing the enhanced efficacy of combining mRNA vaccines with checkpoint inhibitors. The treatments investigated include various mRNA-LNP formulations such as mOVA/CpG NPs, mLuc/CpG NPs, mOVA/p(I:C) NPs, mLuc/p(I:C) NPs, mOVA NPs, and mTRP2/CpG NPs. (C-D) Comparison of predicted immune response and antitumor efficacy of mRNA vaccine strategies with experimental data from Fengqiao Li et al. [37] (2023), highlighting the role of GSDMBNT-encoding mRNA LNPs. The consistent findings between our model and these experimental studies validate the efficacy of mRNA-based strategies in cancer immunotherapy (χ^2^ < 0.0002, observed across all fittings performed for different tumor models and treatment scenarios).

In the absence of clinical data, the model was validated against pre-clinical data by comparing model predictions of tumor growth with independent studies involving mRNA vaccines, including adjunctive therapies, such as ICI [36,37]. To validate the predictive capability of our mechanistic model, we compared the simulation outputs with preclinical data from melanoma and breast cancer murine models. Fig 2 illustrates the correlation between predicted tumor growth dynamics and observed experimental results, demonstrating high consistency across multiple datasets. Additionally, immune response markers, such as CD8 + T cell infiltration levels and cytokine expression profiles, showed strong alignment with published experimental findings (Fig B in S1 Text).

The simulations were conducted using the finite elements software COMSOL Multiphysics (Burlington, MA).

## Results

### Estimating model parameter values and matching with experimental data

Our model incorporates a wide range of biological and pharmacological parameters to simulate the complex interactions between cancer cells, immune cells, and the TME. Most values of model parameters were taken from the literature and are listed in Table A in S1 Text. Key parameters, including the tumor cell proliferation rate parameters (λc, Kc), and pharmacokinetics of mRNA vaccines ($K_{cell-Vaccine}^{on}$, $K_{cell-Vaccine}^{off}$, $K_{protein}$) were estimated by fitting the model to pertinent experimental data and are outlined in Table 1. These parameters were selected because they either depend on cancer cell type (λc, Kc) or capture essential aspects of mRNA vaccine response and their values cannot be found in the literature. For data fitting, we employed a nonlinear least squares optimization method to minimize discrepancies between model predictions and experimental data, focusing on tumor growth and immune cell parameters/data. By systematically adjusting these parameters, we ensured that the model accurately captured the complexity of tumor-immune interactions and treatment responses (Fig 2).

**Table 1:** Overview of model parameters adjusted for validation across cancer cell lines and treatments. The table lists the parameters that were varied to validate the model against experimental data from different studies, categorized by cancer cell line and treatment types. Parameters include those related to tumor growth, and pharmacokinetics of mRNA vaccine delivery.

Ben et al. [36]						
	**Cancer cell line**					
**Parameters**	**B16-OVA**	**B16-F10**				
λc (growth rate parameter) [day − 1]	2.85	3.23				
Kc (growth rate parameter) [mol ∙ m − 3]	0.0082	0.0073				
	**Nanoparticle Formulations**					
	**mOVA/CpG NPs**	**mLuc/CpG NPs**	**mOVA/p(I:C) NPs**	**mLuc/p(I:C) NPs**	**mOVA NPs**	**mTRP2/CpG Np**
$K_{cell-Vaccine}^{on}$ [ml/h] (Binding of the vaccine particles to cells)	17	12	17	12	17	14
$K_{cell-Vaccine}^{off}$ [m^3^/h] (Unbinding of the vaccine particles to cells)	5.22	3.22	5.22	3.22	5.22	4.35
$K_{protein}$ [1/s] (production of antigen from the translation of the mRNA)	8.52e-5	8.52e-5	12.2e-5	12.2e-5	2.52e-5	8.52e-5
**Li et al. [37]**						
	**Cancer cell line**					
	**4T1**	**B16-F10**				
λc (growth rate parameter) [day − 1]	4.85	3.23				
Kc (growth rate parameter) [mol ∙ m − 3]	0.0064	0.0073				
	**Nanoparticle Formulations**					
	**mRNA/LNPs**					
$K_{cell-Vaccine}^{on}$ [ml/h] (Binding of the vaccine particles to cells)	21					
$K_{cell-Vaccine}^{off}$ [m^3^/h] (Unbinding of the vaccine particles to cells)	4.22					
$K_{protein}$ [1/s] (production of antigen from the translation of the mRNA)	9.14e-5					

Specifically, we compared our results with those reported in the studies by Ben et al. [37] (Fig 2A and 2B) and Li et al. [36] (Fig 2C and 2D). These studies provide independent evidence supporting the efficacy of mRNA lipid nanoparticle (LNP) strategies and their role in enhancing cancer immunotherapy. Li and colleagues demonstrated that mRNA LNPs encoding the N-terminus of gasdermin (GSDMBNT) could sensitize tumors to anti-PD-1 immunotherapy. Their findings showed that this approach led to robust antitumor immunity, promoting tumor growth inhibition in two mouse models of melanoma, B16-OVA and B16-F10. Ben and colleagues explored the use of mRNA-LNPs to enhance the efficacy of ICI in murine breast (4T1) and melanoma (B16-F10) tumors. Tumor growth rates were held constant across conditions to provide a consistent baseline for comparing treatment effects. This approach isolates the impact of immunotherapy, avoiding confounding effects due to variable tumor dynamics.

They investigated various nanoparticle formulations, including mOVA/CpG NPs, mLuc/CpG NPs, mOVA/p(I:C) NPs, mLuc/p(I:C) NPs, mOVA NPs, and mTRP2/CpG NPs. These formulations delivered mRNA encoding tumor-associated antigens such as tyrosinase-related protein 2 (TRP2), or model antigens, such as ovalbumin (OVA) combined with adjuvants like CpG and poly(I:C).

To derive the values of the growth parameters λc and Kc, we fitted the tumor growth predictions of the model to the experimental data of the control group for each tumor type and for each study. We kept these values constant across all treatment groups for each study. Therefore, we fitted the remaining three parameters related to vaccine response to each treatment group, ensuring that the model accurately reflected the distinct treatment responses observed in the experimental data. This approach allowed us to capture both the baseline tumor growth characteristics and the specific immune responses elicited by the different therapies.

Apart from the tumor growth results, our model predictions regarding the percentage of CD8 + T cell infiltration align with the experimental data, as shown in Fig B in S1 Text, where the increase in CD8 + T cell levels correlates with improved treatment response.

### Understanding the impact of vaccination timing on immune response

**Fig 3:**
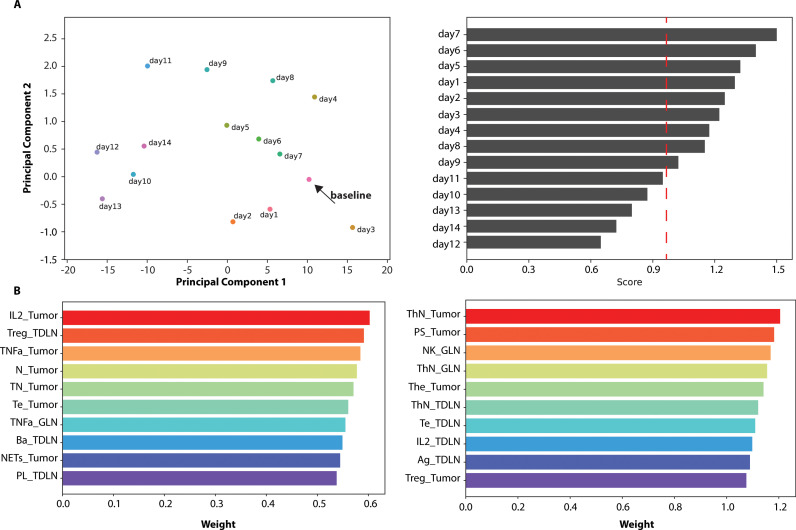
Principal Components Analysis (PCA) and Non-Negative Matrix Factorization (NMF) of Vaccination Outcomes. (A) PCA plot showing the trajectory of immune response in principal component space for various vaccination schedules. Each point represents a different vaccination time point. The separation of trajectories illustrates the impact of vaccination timing on the immune response, highlighting the importance of optimizing vaccination schedules. This general pattern suggests that immune modulation through vaccination timing could be a universal mechanism applicable to other cancer types and immunotherapies. (B) NMF analysis identifying two primary biological programs (Program-1 and Program-2) driving treatment responses. Program-1 includes factors such as IL2 levels in the tumor, Treg cells in the Tumor-Draining Lymph Node (TDLN), TNF-alpha levels in the tumor, neutrophils in the tumor, naïve CD8 + T cells in the tumor, activated CD8 + T cells in the tumor, TNF-alpha in the general lymph nodes, activated B cells in the TDLN, neutrophil extracellular traps (NETs) in the tumor, and long-lived plasma (antibody-secreting) B cells in the TDLN. Program-2 includes factors such as naïve CD4 + T cells (ThN) in the tumor, short-lived plasma (antibody-secreting) B cells in the tumor, natural killer cells in the general lymph nodes, naïve CD4 + T cells in the general lymph nodes, activated CD4 + T cells in the tumor, naïve CD4 + T cells in the TDLN, activated CD8 + T cells in the TDLN, IL2 in the TDLN, antigens in the TDLN, and Treg cells in the tumor. These programs illustrate how specific biological pathways are associated with different treatment responses, highlighting the complexity of the immune response to vaccination and other interventions. The identification of these programs suggests potential biomarkers that could guide therapeutic decisions in clinical practice.

Next, we set out to examine the effect of vaccination timing on immune response. We ran multiple simulations using the baseline values of model parameters (Tables 1 and A in S1 Text), whose value was defined by fitting to the experimental data in Fig 2. In the simulations, we varied the vaccination time from day 1 to day 14 of tumor growth and each simulation ran for 30 days. The results at the end of each simulation correspond to the day 30 outcomes. To examine in detail the disease trajectory and vaccination efficacy, we performed a PCA on the matrix of the output variables predicted by the model. This analysis is useful for visualizing how important parameters are affected by various vaccination schedules. Fig 3A depicts the trajectory in principal component space for each vaccination time point, with the day 30 outcomes. The analysis reveals that varying the day of vaccination initiation leads to distinct immune response trajectories and thus, varying outcomes, as indicated by the separation in principal component space. A trajectory closer to the case of no tumor (baseline) suggests that the immune system has returned to a more regulated or healthy state, indicating a more effective immune response. In this case, the baseline represents a state of immune homeostasis, with lower tumor burden and reduced inflammation, which would signify a successful therapeutic outcome. This separation highlights the importance of optimizing vaccination timing to achieve a trajectory that moves toward this desired state. This suggests that optimizing vaccination timing could improve outcomes not just in the strategy tested here, but potentially in broader cancer immunotherapy strategies. The first two principal components contributed 68% and 21% to the total variance, respectively. Detailed contribution scores for Program-1 and Program-2 components are provided in Table B in S1 Text.

An advantage of our modeling approach is the ability to move from merely observing differential outcomes to understanding the underlying mechanisms that drive these results. To quantify and compare treatment outcomes, we introduced a treatment score, a composite measure derived from key model outputs. The score is calculated based on the Euclidean distance of each daily immune response trajectory in the PCA space from the case with no tumor, serving as a baseline reference. This distance accounts for changes in tumor size reduction, immune cell infiltration, and cytokine levels in the tumor, offering a holistic assessment of treatment efficacy. By integrating these factors, the treatment score provides a quantitative framework for evaluating the effectiveness of different therapeutic scenarios [41]. Higher Treatment Score ratios indicate the specific treatment returned the patient towards baseline status. The generalized interpretation of this score can assist in assessing treatment responses across different patient populations and therapeutic settings.

In order to directly link specific components of the model to biological processes and clinical outcomes, we performed a NMF to identify distinct biological programs responsible for treatment responses. Each output variable in the model represents a measurable biological parameter (such as IL-6 levels in the tumor). The biological programs defined here consist of combinations of model outputs that are strongly associated with a particular outcome. We aimed to decompose the N x M feature matrix into two primary biological programs (k = 2) that represent the key drivers of treatment response. Each biological program is a group of factors that work together to influence whether a treatment will be successful. The treatment score, used to classify these outcomes, was calculated based on the RECIST criteria (Response Evaluation Criteria in Solid Tumors), which is commonly used in clinical settings to measure tumor response. The score is based on changes in tumor volume, with a reduction of more than 30% classified as a response. The duration of therapy before quantifying response probability was chosen based on preclinical studies, ensuring sufficient time to capture meaningful changes in tumor volume and immune response. We then separately identified dominant programs for different treatment timing scenarios that led to favorable outcomes versus those that did not, using the treatment score defined above. One of the identified programs (Program-1) includes factors such as the IL2 level in the tumor, the amount of Treg on the Tumor-draining Lymph node (TDLN), TNFa in the Tumor, Neutrophils in the Tumor, naïve CD8 + T cells in the Tumor, Activated CD8 + T cells in the Tumor, TNFa in the General Lymph Nodes, Activated B cells in Tumor Draining Lymph Nodes, Neutrophils Extracellular Traps (NETs) in the Tumor, and Long-lived plasma (antibody-secreting) B cells (PL) in Tumor Draining Lymph Nodes (Fig 3B). Another program (Program-2) includes factors such as naïve CD4 + T cells (ThN) in the Tumor, Short-lived plasma (antibody-secreting) B cells (PS) in the Tumor, Natural Killer Cells in General Lymph Nodes, naïve CD4 + T cells in General Lymph Nodes, Activated CD4 + T cells in the Tumor, naïve CD4 + T cells in Tumor Draining Lymph Nodes, Activated CD8 + T cells in Tumor Draining Lymph Nodes, IL2 in Tumor Draining Lymph Nodes, Antigen in Tumor Draining Lymph Nodes and Treg in the Tumor. These programs illustrate how specific biological pathways are associated with different treatment responses, highlighting the complexity of the immune response to vaccination and other interventions (Fig 3B). These programs illustrate the complex interplay of immune pathways influencing treatment outcomes, with implications for tailoring immunotherapies in different clinical contexts.

### Biomarkers of response probabilities across different therapies and tumor types

Virtual patient cohorts generated through Latin hypercube sampling provided a comprehensive dataset for in-silico trials. This approach enabled us to explore the impact of key parameters, such as APC density, cytokine production, and extracellular matrix (ECM) density, on mRNA vaccine efficacy. The statistical distributions of these virtual cohorts also allowed for subgroup analyses, identifying predictive biomarkers and validating model predictions.

To explore the effects of different therapies on biomarker response probabilities in cancer treatment, we analyzed pre-treatment and on-treatment biomarker data for melanoma and breast cancer using a heatmap visualization approach [42,43]. The response probabilities for each biomarker were evaluated for three different therapies: mRNA vaccine, immune checkpoint inhibition (ICI), and the combination of mRNA vaccine with immune checkpoint inhibition (mRNA + ICI).

**Fig 4:**
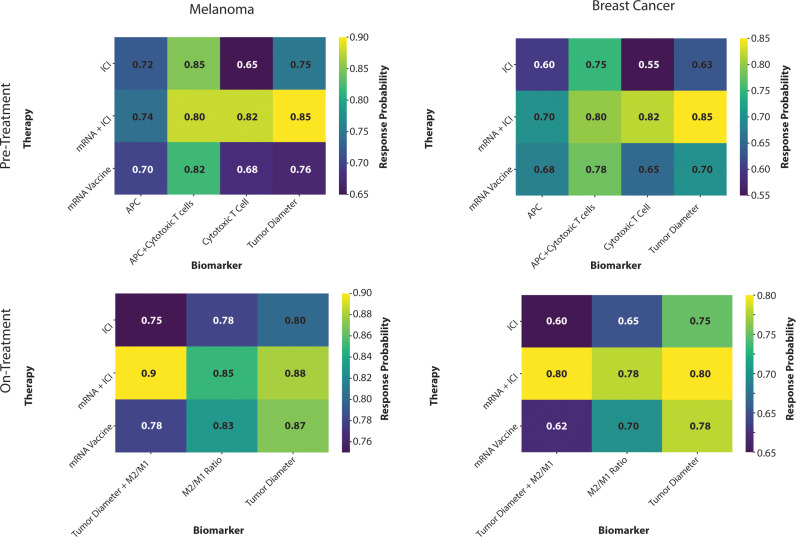
Pre-Treatment and On-Treatment Biomarker Response Probabilities for Different Therapies in Melanoma and Breast Cancer. Heatmap showing the response probabilities of pre-treatment and on-treatment biomarkers in melanoma and breast cancer for three different therapies: mRNA vaccine, ICI, and mRNA + ICI combination. Melanoma Pre-Treatment: The response probabilities are shown for four biomarkers: APC Density, Cytotoxic T Cell Fraction, Tumor Diameter, and Combined (APC + Cytotoxic T Cells). Higher response probabilities are observed for combination therapy across most biomarkers, indicating its superior efficacy in priming the immune system. Breast Pre-Treatment: The combination of mRNA Vaccine with ICI shows the highest efficacy across all biomarkers, particularly in enhancing APC Density and reducing Tumor Diameter. Melanoma On-Treatment: The biomarkers analyzed include Relative Change in Tumor Diameter, M2/M1 Macrophage Ratio, and Combined (Tumor Diameter + M2/M1). The combination therapy shows the most significant effects across all biomarkers, suggesting a potent immune response and tumor suppression. Breast On-Treatment: The combination therapy consistently demonstrates higher response probabilities, particularly in modulating the TME and enhancing immune response, compared to single therapies.

Pre-Treatment Biomarkers: For melanoma (using parameter values for melanoma B16-F10 from Table 1), the heatmap of pre-treatment biomarkers (Fig 4) shows that the combination of mRNA Vaccine and ICI (mRNA + ICI) consistently yielded the highest response probabilities across all biomarkers, particularly for the combined metric of APC Density in tumor and Cytotoxic T Cell fraction in the tumor. The mRNA vaccine treatment alone demonstrated moderate efficacy, particularly in increasing the densities of APC and Cytotoxic T Cells. In contrast, the ICI alone showed lower response probabilities, especially in tumor diameter reduction. In breast cancer (using parameter for 4T1 breast cancer from Table 1) (Fig 4), a similar trend was observed, where the mRNA + ICI combination therapy outperformed other treatments in enhancing the pre-treatment biomarker response, with significant improvements in both the combined APC + Cytotoxic T Cell fraction and tumor diameter metrics. The ICI alone showed the lowest response probability, indicating a less effective modulation of pre-treatment biomarkers compared to combination therapy or mRNA Vaccine alone.

On-Treatment Biomarkers: Analysis of on-treatment biomarkers revealed distinct patterns for both tumor types. In melanoma (Fig 4), the combination therapy (mRNA + ICI) resulted in the highest response probabilities for all on-treatment biomarkers, including relative change in tumor diameter, M2/M1 macrophage ratio, and the combined metric (Tumor Diameter + M2/M1). This suggests a robust immune activation and tumor suppression when combining mRNA vaccines with ICI. The mRNA vaccine alone also showed effective modulation of on-treatment biomarkers, particularly in reducing the M2/M1 macrophage ratio, while ICI alone demonstrated moderate effects. For breast cancer (Fig 4), a consistent pattern was observed where the mRNA + ICI therapy exhibited superior efficacy in modulating on-treatment biomarkers, as evidenced by high response probabilities across all metrics. The mRNA vaccine alone showed a moderate effect, particularly in affecting the relative change in tumor diameter, whereas the ICI alone had the least impact on on-treatment biomarkers, suggesting limited effectiveness. These findings highlight the significant potential of combination therapies in enhancing biomarker response, thereby improving clinical outcomes in cancer treatment.

### Impact of TME characteristics on vaccine efficacy

To evaluate how TME influences the efficacy of various cancer immunotherapy strategies, we analyzed response probabilities across different levels of ECM density and immune cell activity for two tumor types: melanoma and breast cancer. Virtual patient cohorts were generated using Latin hypercube sampling, ensuring diverse parameter distributions that reflect variations in ECM properties, immune dynamics, and therapeutic responses. Simulations were run for 30 days, with treatments initiated on day 7. The therapies assessed included mRNA Vaccine, ICI, and a combination of mRNA Vaccine with ICI (mRNA + ICI). For mRNA vaccines, administration was modeled as a single dose delivered on day 7, while four cycles of ICIs were modeled on days 8, 11, 14 and 17. The response probabilities were calculated using RECIST criteria, which evaluate tumor volume changes to quantify treatment efficacy.

In this model, ECM density variations were implemented by modifying tumor stiffness and hydraulic conductivity. High ECM density corresponded to a tenfold increase in stiffness and a tenfold decrease in hydraulic conductivity from baseline values, while low ECM density represented the inverse adjustments. Immune cell activity was simulated by altering the conversion rate of Naïve T and B cells to activated cells, reflecting enhanced immune responses under conditions of higher activity.

**Fig 5:**
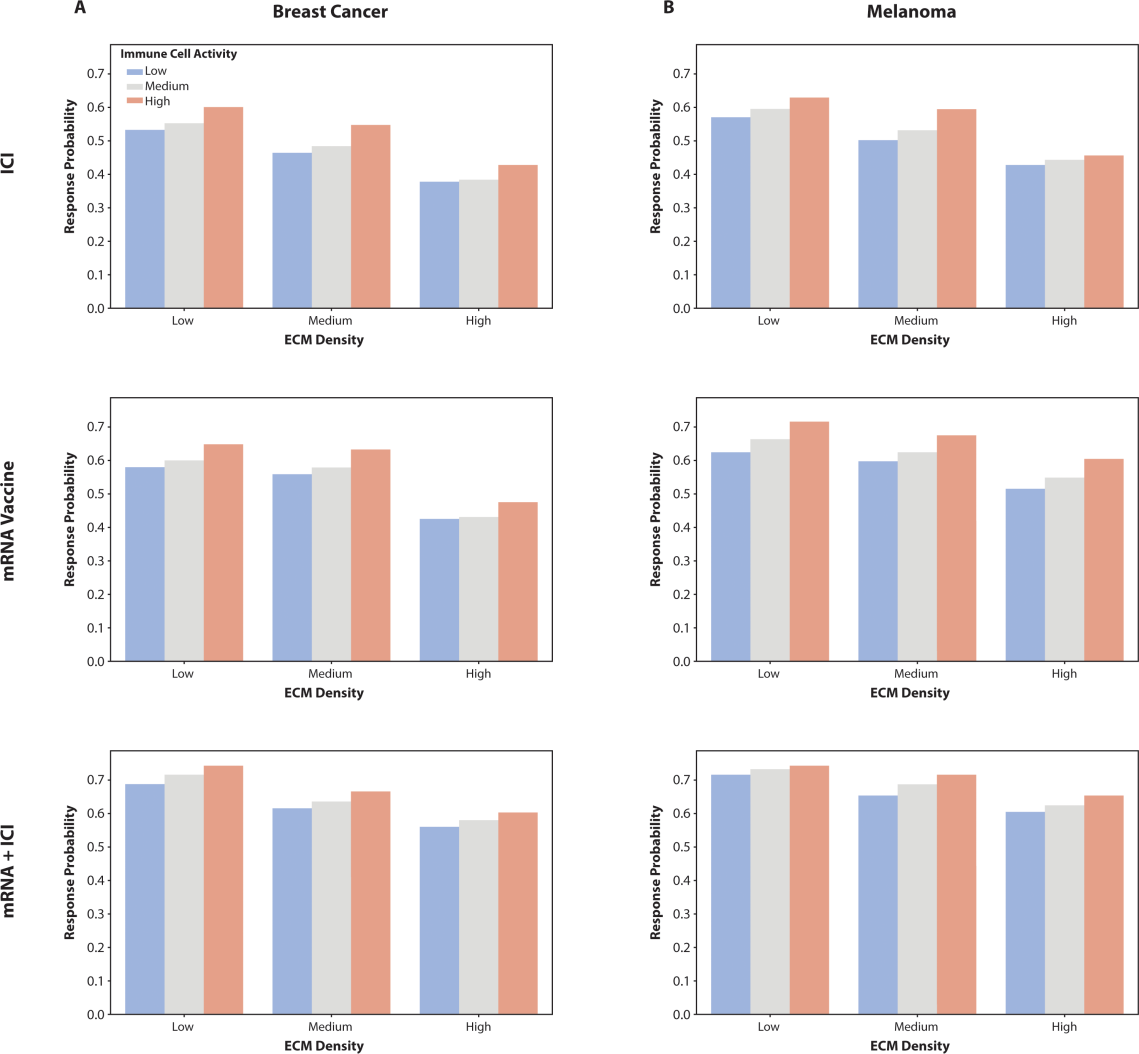
Impact of TME Factors on Vaccine Efficacy in Melanoma and Breast Cancer. (A) Bar charts showing the response probabilities of breast cancer for three different therapies (mRNA Vaccine, ICI, mRNA + Checkpoint) across varying levels of ECM density and immune cell activity. Higher response probabilities are observed with increased ECM density and immune cell activity, with the mRNA + ICI combination therapy demonstrating superior efficacy across all conditions. (B) Bar chart illustrating the response probabilities for melanoma under the same three therapies. The mRNA + ICI combination therapy consistently shows the highest response probabilities, particularly at higher levels of ECM density and immune cell activity, indicating its effectiveness in enhancing immune response in the tumor microenvironment.

Fig 5 illustrates the combined effects of ECM density and immune cell activity on response probabilities for melanoma and breast cancer under the three therapies. Across both tumor types, mRNA + ICI combination therapy consistently demonstrated the highest response probabilities under all conditions, particularly at low ECM density and high immune cell activity levels. This highlights the synergistic potential of combining mRNA vaccines with ICIs. The mRNA Vaccine therapy showed intermediate efficacy, with response probabilities increasing at low ECM density and medium immune cell activity levels, emphasizing the role of reduced ECM stiffness and moderate immune stimulation in enhancing vaccine efficacy. In contrast, ICI therapy alone produced the lowest response probabilities, suggesting its limited capacity to modify the TME compared to the other therapies. Specifically, we observed that low ECM density facilitates greater immune cell infiltration, particularly of CD8 + T cells, due to reduced physical barriers. This is consistent with experimental evidence showing that a dense ECM can impede T cell migration, thereby limiting immune surveillance and cytotoxic activity within tumors [44,45].

These results underscore the importance of optimizing the TME by modulating ECM density and immune cell activity to improve the therapeutic efficacy of mRNA-based and ICI therapies. Presenting these findings for melanoma and breast cancer together reveals consistent trends, demonstrating the generalizability of these observations across tumor types while avoiding unnecessary repetition.

### Cytokine production and its impact on vaccine efficacy across different ECM densities

To further understand the role of cytokine production in modulating vaccine efficacy, we analyzed the response probabilities for melanoma and breast cancer across different levels of ECM density and cytokine production (baseline x10 for high cytokine production and/10 for low cytokine production), focusing specifically on low ECM density and medium (baseline values) cytokine production conditions.

**Fig 6:**
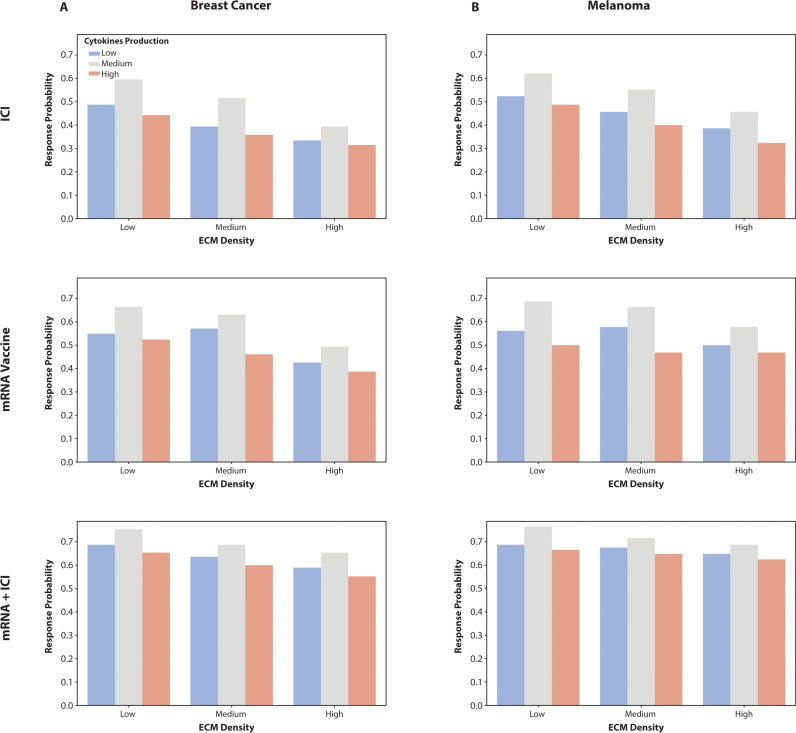
Influence of ECM Density and Cytokine Production on Vaccine Efficacy in Melanoma and Breast Cancer. (A) Bar charts showing the response probabilities for breast cancer under three different therapies (mRNA Vaccine, ICI, mRNA + ICI) across varying levels of ECM density and cytokine production. The results highlight the enhanced efficacy of all therapies, particularly the mRNA + ICI combination, under low ECM density and medium cytokine production conditions, indicating an optimal environment for immune activation. (B) Bar chart illustrating the response probabilities for melanoma under the same therapeutic conditions. The figure shows that medium cytokine production in a low ECM density setting improves response probabilities across all therapies, with the mRNA + ICI combination therapy achieving the highest efficacy. This suggests that a balanced cytokine milieu enhances the immune response in the tumor microenvironment, particularly for mRNA-based therapies.

Effect of Cytokine Production on Vaccine Efficacy in Low ECM Density: Under conditions of low ECM density, medium levels of cytokine production in the tumor were found to significantly enhance vaccine efficacy across all therapies for both melanoma and breast cancer (Fig 6). In melanoma, the mRNA Vaccine therapy showed improved response probabilities when cytokine production was at medium levels, suggesting an optimal environment for immune activation. This effect was more pronounced in the mRNA + ICI combination therapy, which demonstrated the highest response probabilities, highlighting its ability to potentiate immune response even under less favorable ECM conditions. The ICI alone, while showing lower overall response probabilities, also benefited from medium cytokine production, indicating that a balanced cytokine milieu can enhance the effectiveness of this therapy. In breast cancer, a similar trend was observed where medium cytokine production under low ECM density conditions led to increased response probabilities for all therapies (Fig 6). The mRNA Vaccine and mRNA + ICI combination therapies were particularly effective, showing the highest response probabilities in this setting. This suggests that medium cytokine production creates an optimal inflammatory environment that supports effective antigen presentation and T cell activation, crucial for the success of these therapies. While inflammation is generally desirable for immunotherapy due to its role in activating immune responses, excessive cytokine levels can lead to uncontrolled inflammation, which may promote immunosuppressive mechanisms in the tumor microenvironment and contribute to immune cell exhaustion, ultimately diminishing therapeutic efficacy. The ICI, although less effective overall, still demonstrated a notable increase in efficacy under medium cytokine production conditions, reinforcing the importance of a balanced cytokine environment in enhancing immune checkpoint blockade. Furthermore, cytokine dynamics were shown to play a pivotal role in modulating the immune response. Our model demonstrated that medium levels of pro-inflammatory cytokines, such as IFN-γ and TNF-α, under conditions of low ECM density, create an optimal inflammatory environment that enhances T cell activation and effector functions. In contrast, excessively high cytokine levels can lead to immune exhaustion, while low cytokine levels fail to adequately stimulate immune responses. Medium cytokine levels refer to IL-2 and TNF-α concentrations within the range of 10–50 pg/ml, which were found to optimize immune activation without triggering immune exhaustion.

These findings underscore the critical role of cytokine production in shaping the TME and its impact on the efficacy of different immunotherapies. The results suggest that maintaining a medium level of cytokine production, particularly under low ECM density conditions, can optimize the immune response to mRNA-based vaccines and combination therapies, ultimately improving clinical outcomes in cancer treatment.

### Impact of vaccine uptake on efficacy in different ECM density environments

To assess how vaccine uptake influences the effectiveness of cancer immunotherapies, we analyzed response probabilities in melanoma and breast cancer under conditions of low, medium, high ECM density and low, medium, high vaccine uptake (baseline x10 for high vaccine uptake and/10 for low vaccine uptake). This analysis focused on how enhanced vaccine uptake can potentially overcome the barriers posed by a less dense ECM to improve treatment outcomes.

**Fig 7:**
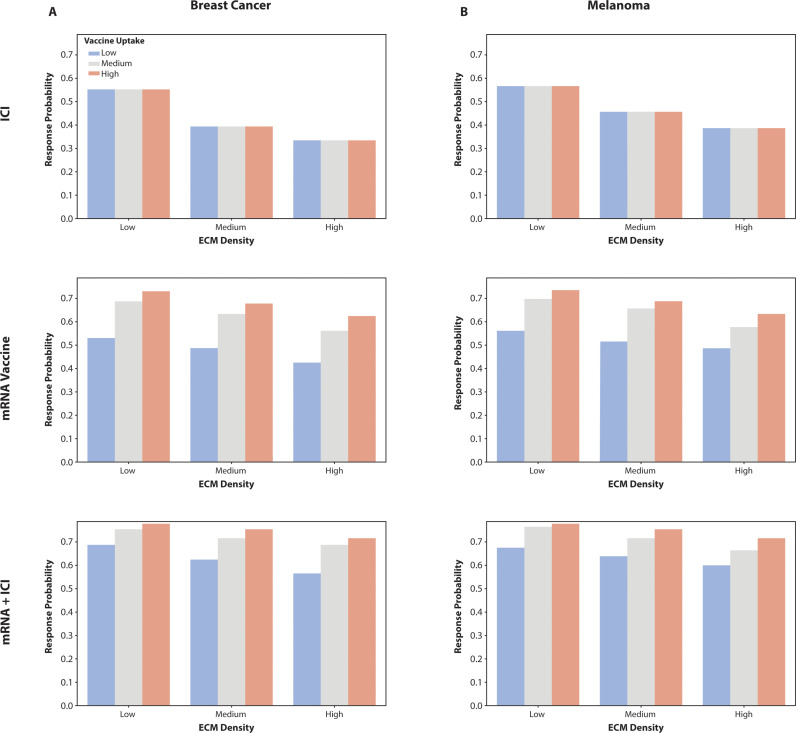
Impact of Vaccine Uptake on Efficacy Across Different Therapies in Low ECM Density Environments. (A) Bar chart showing the response probabilities for breast cancer under low ECM density conditions with high vaccine uptake across three therapies: mRNA Vaccine, ICI, and mRNA + ICI combination. The results indicate that high vaccine uptake significantly enhances treatment efficacy, with the mRNA + ICI combination therapy demonstrating the highest response probabilities, suggesting superior immune activation and tumor suppression. (B) Bar chart illustrating the response probabilities for melanoma under similar conditions. High vaccine uptake leads to improved response probabilities across all therapies, with the mRNA + ICI combination therapy showing the most pronounced effect, followed by the mRNA Vaccine alone. These findings underscore the importance of maximizing vaccine uptake to optimize therapeutic outcomes in low ECM density environments.

High vaccine uptake was associated with significantly increased response probabilities across all therapies, including mRNA therapy and the mRNA + ICI combination therapy, for both melanoma and breast cancer (Fig 7). In both tumor types, the mRNA Vaccine alone demonstrated a marked improvement in efficacy when vaccine uptake was high, indicating that efficient delivery and uptake of the vaccine are crucial for mounting a robust immune response. This effect was further amplified in the mRNA + ICI combination therapy, which consistently exhibited the highest response probabilities. The dual mechanisms of immune activation through mRNA vaccines and inhibition of immune checkpoints by ICIs effectively modified the TME to enhance anti-tumor immunity, even under conditions of high ECM density. The trend was consistent across both melanoma and breast cancer, with high vaccine uptake leading to superior response probabilities compared to conditions of medium or low uptake. The mRNA Vaccine showed significant benefits from high vaccine uptake in both tumor types, resulting in a more robust immune response. The mRNA + ICI combination therapy leveraged the synergistic effects of vaccine uptake and immune checkpoint inhibition, achieving the highest therapeutic efficacy among all therapies. Although ICI therapy alone demonstrated lower overall efficacy, it also showed improved outcomes with high vaccine uptake, underscoring the importance of efficient vaccine delivery in enhancing treatment responses. These findings emphasize that high vaccine uptake is a critical determinant of cancer immunotherapy efficacy, particularly in the context of dense ECM environments. Strategies to improve vaccine uptake could significantly enhance therapeutic outcomes, especially when combined with immune checkpoint inhibitors. These results highlight the potential for optimizing vaccine delivery to boost the effectiveness of standalone and combination immunotherapy regimens in cancer treatment.

### Mechanistic insights into immune cell infiltration with extra variation

To gain a deeper understanding of how immune cell dynamics affect treatment response, we analyzed the predicted by the model densities of cytotoxic T cells and M2 macrophages, introducing additional variability to reflect the diversity in immune cell infiltration patterns across different patient responses. The added variation was introduced by altering the production rate of macrophages by pro-inflammatory cytokines. Specifically, the baseline production rates were varied by ±10%, simulating the natural heterogeneity in how patients’ immune systems respond to inflammatory signals within the tumor microenvironment. This variability captures differences in macrophage recruitment, activation, and polarization into either M1 or M2 phenotypes, which can significantly impact immune response and treatment outcomes. Responders and non-responders were defined based on tumor volume reduction using the RECIST (Response Evaluation Criteria in Solid Tumors) criteria. Responders were classified as patients whose tumor volume decreased by more than 30% following treatment, while non-responders were those whose tumor volume did not meet this threshold for reduction, or in cases where tumor progression occurred. This classification allows for a clear distinction between effective and ineffective immune responses to the mRNA vaccine or combination therapy. The scatter plot (Fig C in S1 Text) visualizes the relationship between cytotoxic T cell density and M2 macrophage density in both responders and non-responders. The data indicate that high cytotoxic T cell density is predominantly associated with responders, as shown by the clustering of data points in the upper region of the scatter plot. Conversely, non-responders typically exhibit lower cytotoxic T cell densities, as evidenced by the concentration of points in the lower density range. Additionally, responders tend to have lower M2 macrophage densities, while non-responders display higher M2 macrophage densities. These observations suggest that a favorable immune response is characterized by a high density of cytotoxic T cells and a low density of immunosuppressive M2 macrophages. The added variation in the data highlights the diverse immune cell infiltration patterns that can exist within each response category, reinforcing the complexity of the tumor-immune interactions and the importance of immune cell composition in determining treatment outcomes.

### Subgroup analysis by tumor type

To further explore the differential responses to treatment across various tumor types, we conducted a subgroup analysis comparing response probabilities between two groups: melanoma and breast cancer for the purpose of this analysis. The box plot (Fig C in S1 Text) presents the response probabilities for each subgroup.

The results show that the melanoma group exhibits a higher median response probability compared to breast cancer, with a narrower interquartile range, suggesting more consistent treatment responses in melanoma. In contrast, breast cancer has a slightly lower median response probability and a broader interquartile range, indicating greater variability in treatment outcomes. These findings suggest that tumor type plays a significant role in determining response to treatment, with melanoma patients generally showing a higher and more uniform probability of response. Overall, these analyses provide valuable mechanistic insights into the factors influencing treatment efficacy, highlighting the critical role of immune cell densities and the impact of tumor type on clinical outcomes. The observed variability within and between groups underscores the need for personalized treatment strategies tailored to individual tumor and immune profiles.

## Discussion

Our comprehensive mechanistic math model reveals the potential of mRNA vaccines in complex tumor microenvironments. The model predictions were validated using experimental data from two independent studies [36,37] providing further support for our model.

Comparisons with the data Li et al. [ref 35] showed that mRNA LNPs encoding the N-terminus of gasdermin (GSDMBNT) could induce pyroptosis and convert immunologically cold tumors into hot ones, enhancing response to anti-PD-1 immunotherapy. Our model similarly predicted that mRNA vaccines could effectively prime the immune system in various TMEs, aligning with these experimental outcomes. Elana Ben et al. [36] further demonstrated that combining mRNA vaccines with ICI enhances antigen presentation and T cell activation. Our model predicted tumor regression with such combinations, validating its predictive power. To further examine the mechanisms underlying these predictions, we explored the impact of TME characteristics, specifically hydraulic conductivity and stiffness. Altering these parameters led to differential outcomes within the same treatment groups. For instance, increasing hydraulic conductivity (baseline x10) and reducing stiffness (baseline/10) significantly improved treatment responses by facilitating immune cell infiltration and enhancing mRNA vaccine uptake. We found that lowering TME resistance through modifications in hydraulic conductivity and stiffness may be a viable approach to improve treatment efficacy. By adjusting these TME properties, our model indicates that targeted strategies to reduce tumor stiffness and/or enhance hydraulic conductivity could support and amplify mRNA vaccine effectiveness. PCA further revealed that the timing of vaccination significantly impacts immune responses. Specific initiation days showed closer proximity to baseline immune activity, indicating an optimal response. NMF identified distinct biological programs, showing how factors like cytotoxic T cell and M2 macrophage densities influence treatment outcomes. These analyses highlight the importance of optimizing vaccination timing and understanding underlying immune dynamics.

Our model offers a number of guidelines for improving mRNA vaccine-based therapies. First, ECM density and immune cell activity are crucial: low ECM density and high immune cell activity correlated with enhanced response probabilities, especially for combination therapies. Second, optimizing cytokine production and maximizing vaccine uptake under low ECM density conditions were associated with improved vaccine efficacy, suggesting that fine-tuning these parameters could improve outcomes. These insights provide preliminary guidelines for refining mRNA vaccine approaches in oncology, suggesting that reprogramming the TME, selecting optimal vaccination times, and modifying cytokine production should enhance therapeutic outcomes. Subgroup analysis highlighted that melanoma patients generally show higher and more consistent response probabilities than breast tumor types, emphasizing the need for tumor-specific treatment strategies.

In addition to expected findings, our model revealed that medium cytokine production, rather than high, was associated with reduced immune cell exhaustion, enhancing vaccine efficacy. Similarly, hydraulic conductivity is increased by tenfold amplified immune cell infiltration, but only in combination with reduced ECM stiffness. These findings suggest that fine-tuning TME properties can optimize treatment outcomes and highlight the need for tailored therapeutic interventions in distinct tumor contexts. A tenfold increase in hydraulic conductivity was used to simulate improved vascular perfusion and ECM remodeling, as observed experimentally with anti-VEGF therapies and collagenase treatments [14,18,46–48]. This level of enhancement is within the range reported in preclinical tumor models.

While our mechanistic model provides valuable insights into mRNA vaccine efficacy and optimization in oncology, it has several limitations. First, the model is based on a set of assumptions regarding TME characteristics, immune cell interactions, and vaccine pharmacodynamics, which may not fully capture the complexities present in diverse patient populations. For example, the model does not account for the full heterogeneity in patient immune profiles, such as differences in baseline immune status or prior treatments, which could significantly impact vaccine response. Additionally, certain parameters, such as hydraulic conductivity and stiffness, were varied to simulate TME conditions, but these adjustments may oversimplify the dynamic and adaptive nature of the TME in vivo. Another limitation is the reliance on pre-clinical data and virtual cohorts, which, although informative, may not fully translate to human clinical settings. Finally, while the model includes multiple immune cell types, it does not capture the full spectrum of immune regulatory networks or potential off-target effects, which could influence the therapeutic outcomes of mRNA vaccines in more complex biological systems. Furthermore, we have excluded dynamic tumor clonal evolution and adaptive immune escape mechanisms from our simulations. These processes play critical roles in cancer progression and treatment resistance [49,50]. Dynamic clonal evolution can lead to the emergence of resistant tumor subclones [49,50], while adaptive immune escape mechanisms enable tumors to modulate antigen expression or immune checkpoint pathways, thereby evading immune surveillance [49,50]. The absence of these factors may limit the model’s ability to fully capture the complexity of tumor-immune dynamics over prolonged treatment durations. Future models could incorporate these mechanisms to enhance predictive accuracy, particularly in scenarios involving immunotherapy resistance or tumor relapse [33,34]. Nonetheless, our current model focuses on key TME characteristics that significantly influence early vaccine response, providing a foundational framework for further refinement. Moreover, another limitation of our model is the absence of cell-state switching dynamics among tumor and immune cells. While this simplification allows us to focus on the key interactions between the vaccine and the immune system, it might overlook the complexity introduced by phenotypic plasticity, which can significantly influence tumor progression and immune responses [11,14]. Future model iterations could incorporate cell-state transitions to provide a more comprehensive representation of tumor-immune dynamics, as demonstrated in models exploring phenotypic switching and immune evasion mechanisms [49,50]. The model also excludes other immunosuppressive populations such as regulatory B cells and myeloid-derived suppressor cells (MDSCs), which may impact vaccine efficacy and immune response. The model does not currently account for spatial gradients of oxygen, cytokines, or immune cells, which are known to significantly influence tumor-immune dynamics. Future studies that incorporate spatial modeling may reveal how TME heterogeneity influences tumor-immune dynamics.

Our study demonstrates the potential of mechanistic modeling to advance mRNA vaccine-based cancer immunotherapy by providing a detailed understanding of the complex interactions between the TME, immune dynamics, and therapeutic strategies. The validation of our model with experimental data, combined with insights from advanced analyses, offers a robust framework for optimizing vaccination schedules and developing more effective, personalized cancer treatments. Future research should focus on integrating data from diverse patient populations and exploring combination strategies with other therapeutic modalities to further refine and enhance cancer immunotherapy approaches.

## Supporting information

S1 Text **Fig A.** Flowchart of the Simulation Workflow. This flowchart illustrates the step-by-step process of our simulation framework, starting from initial parameter selection and model setup, followed by execution of simulations, data analysis, and validation with experimental data. Each step ensures a systematic and reproducible methodology for assessing the efficacy of mRNA vaccines and immune checkpoint inhibitors in a computational framework. **Fig B.** Comparison of Mechanistic Model Predictions with Experimental CD8 + T Cell Data. Comparison of model-predicted CD8 + T cell percentages in tumors with experimental data from two independent studies. This comparison highlights the model’s accuracy in replicating immune response patterns observed in pre-clinical settings. **Fig C.** (A) Immune Cell Infiltration Patterns in Responders vs. Non-Responders. Scatter plot showing the relationship between cytotoxic T cell and M2 macrophage densities in virtual patients. High T cell and low M2 macrophage densities are linked to favorable vaccine response. (B) Tumor-Type-Specific Response Probabilities. Box plot comparing melanoma and breast cancer subgroups. Melanoma shows higher and more consistent response probabilities, emphasizing tumor-type impact on treatment efficacy. **Table A.** Model Parameter Values. This table provides a comprehensive list of all model parameters, their descriptions, units, and literature references. Parameters include biological rates, cytokine production, immune cell dynamics, vaccine uptake metrics, and tumor microenvironment properties. **Table B.** Contribution Scores.
(DOCX)

## References

[1] Volkman HR, Nguyen JL, Mustapha MM, Yang J, Jodar L, McLaughlin JM. Effectiveness of a single COVID-19 mRNA vaccine dose in individuals with prior SARS-CoV-2 infection: a systematic review. Commun Med (Lond). 2025;5(1):151. doi: 10.1038/s43856-025-00882-y 40319136 PMC12049417

[2] Wong BK-F, Mabbott NA. Systematic review and meta-analysis of COVID-19 mRNA vaccine effectiveness against hospitalizations in adults. Immunother Adv. 2024;4(1):ltae011. doi: 10.1093/immadv/ltae011 39703784 PMC11655844

[3] Liu C, Shi Q, Huang X, Koo S, Kong N, Tao W. mRNA-based cancer therapeutics. Nat Rev Cancer. 2023;23(8):526–43. doi: 10.1038/s41568-023-00586-2 37311817

[4] Swetha K, Kotla NG, Tunki L, Jayaraj A, Bhargava SK, Hu H, et al. Recent Advances in the Lipid Nanoparticle-Mediated Delivery of mRNA Vaccines. Vaccines (Basel). 2023;11(3):658. doi: 10.3390/vaccines11030658 36992242 PMC10059764

[5] Wu L, Li X, Qian X, Wang S, Liu J, Yan J. Lipid Nanoparticle (LNP) Delivery Carrier-Assisted Targeted Controlled Release mRNA Vaccines in Tumor Immunity. Vaccines (Basel). 2024;12(2):186. doi: 10.3390/vaccines12020186 38400169 PMC10891594

[6] Hou X, Zaks T, Langer R, Dong Y. Lipid nanoparticles for mRNA delivery. Nat Rev Mater. 2021;6(12):1078–94. doi: 10.1038/s41578-021-00358-0 34394960 PMC8353930

[7] Ramachandran S, Satapathy SR, Dutta T. Delivery Strategies for mRNA Vaccines. Pharmaceut Med. 2022;36(1):11–20. doi: 10.1007/s40290-021-00417-5 35094366 PMC8801198

[8] Mellman I, Chen DS, Powles T, Turley SJ. The cancer-immunity cycle: Indication, genotype, and immunotype. Immunity. 2023;56(10):2188–205. doi: 10.1016/j.immuni.2023.09.011 37820582

[9] Barbier AJ, Jiang AY, Zhang P, Wooster R, Anderson DG. The clinical progress of mRNA vaccines and immunotherapies. Nat Biotechnol. 2022;40(6):840–54. doi: 10.1038/s41587-022-01294-2 35534554

[10] Wang Q, Shao X, Zhang Y, Zhu M, Wang FXC, Mu J, et al. Role of tumor microenvironment in cancer progression and therapeutic strategy. Cancer Med. 2023;12(10):11149–65. doi: 10.1002/cam4.5698 36807772 PMC10242329

[11] Pandey PR, Young KH, Kumar D, Jain N. RNA-mediated immunotherapy regulating tumor immune microenvironment: next wave of cancer therapeutics. Mol Cancer. 2022;21(1):58. doi: 10.1186/s12943-022-01528-6 35189921 PMC8860277

[12] He Q, Gao H, Tan D, Zhang H, Wang J-Z. mRNA cancer vaccines: Advances, trends and challenges. Acta Pharm Sin B. 2022;12(7):2969–89. doi: 10.1016/j.apsb.2022.03.011 35345451 PMC8942458

[13] Sayour EJ, Boczkowski D, Mitchell DA, Nair SK. Cancer mRNA vaccines: clinical advances and future opportunities. Nat Rev Clin Oncol. 2024;21(7):489–500. doi: 10.1038/s41571-024-00902-1 38760500

[14] Fukumura D, Kloepper J, Amoozgar Z, Duda DG, Jain RK. Enhancing cancer immunotherapy using antiangiogenics: opportunities and challenges. Nat Rev Clin Oncol. 2018;15(5):325–40. doi: 10.1038/nrclinonc.2018.29 29508855 PMC5921900

[15] Luo Z, Yao X, Li M, Fang D, Fei Y, Cheng Z, et al. Modulating tumor physical microenvironment for fueling CAR-T cell therapy. Adv Drug Deliv Rev. 2022;185:114301. doi: 10.1016/j.addr.2022.114301 35439570

[16] Martin JD, Seano G, Jain RK. Normalizing Function of Tumor Vessels: Progress, Opportunities, and Challenges. Annu Rev Physiol. 2019;81:505–34. doi: 10.1146/annurev-physiol-020518-114700 30742782 PMC6571025

[17] Pal K, Sheth RA. Engineering the Tumor Immune Microenvironment through Minimally Invasive Interventions. Cancers (Basel). 2022;15(1):196. doi: 10.3390/cancers15010196 36612192 PMC9818918

[18] Henke E, Nandigama R, Ergün S. Extracellular Matrix in the Tumor Microenvironment and Its Impact on Cancer Therapy. Front Mol Biosci. 2020;6:160. doi: 10.3389/fmolb.2019.00160 32118030 PMC7025524

[19] Mpekris F, Panagi M, Charalambous A, Voutouri C, Stylianopoulos T. Modulating cancer mechanopathology to restore vascular function and enhance immunotherapy. Cell Reports Medicine. 2024. 10.1016/j.xcrm.2024.101626 PMC11293360 38944037

[20] Xie Y-J, Liu W-Q, Li D, Hou J-C, Coghi PS, Fan X-X. Overcoming Suppressive Tumor Microenvironment by Vaccines in Solid Tumor. Vaccines (Basel). 2023;11(2):394. doi: 10.3390/vaccines11020394 36851271 PMC9964970

[21] Li X, Ma S, Gao T, Mai Y, Song Z, Yang J. The main battlefield of mRNA vaccine - Tumor immune microenvironment. Int Immunopharmacol. 2022;113(Pt A):109367. doi: 10.1016/j.intimp.2022.109367 36327875

[22] Rojas LA, Sethna Z, Soares KC, Olcese C, Pang N, Patterson E, et al. Personalized RNA neoantigen vaccines stimulate T cells in pancreatic cancer. Nature. 2023;618(7963):144–50. doi: 10.1038/s41586-023-06063-y 37165196 PMC10171177

[23] Harkos C, Hadjigeorgiou AG, Voutouri C, Kumar AS, Stylianopoulos T, Jain RK. Using mathematical modelling and AI to improve delivery and efficacy of therapies in cancer. Nat Rev Cancer. 2025;25(5):324–40. doi: 10.1038/s41568-025-00796-w 39972158 PMC12892427

[24] Zhao C, Heuslein JL, Zhang Y, Annex BH, Popel AS. Dynamic Multiscale Regulation of Perfusion Recovery in Experimental Peripheral Arterial Disease: A Mechanistic Computational Model. JACC Basic Transl Sci. 2022;7(1):28–50. doi: 10.1016/j.jacbts.2021.10.014 35128207 PMC8807862

[25] Popel AS. Immunoactivating the tumor microenvironment enhances immunotherapy as predicted by integrative computational model. Proc Natl Acad Sci U S A. 2020;117(9):4447–9. doi: 10.1073/pnas.2001050117 32102915 PMC7060734

[26] Zhao C, Zhang Y, Popel AS. Mechanistic Computational Models of MicroRNA-Mediated Signaling Networks in Human Diseases. Int J Mol Sci. 2019;20(2):421. doi: 10.3390/ijms20020421 30669429 PMC6358731

[27] Peskov K, Azarov I, Chu L, Voronova V, Kosinsky Y, Helmlinger G. Quantitative Mechanistic Modeling in Support of Pharmacological Therapeutics Development in Immuno-Oncology. Front Immunol. 2019;10:924. doi: 10.3389/fimmu.2019.00924 31134058 PMC6524731

[28] Voutouri C, Hardin CC, Naranbhai V, Nikmaneshi MR, Khandekar MJ, Gainor JF, et al. Mechanistic model for booster doses effectiveness in healthy, cancer, and immunosuppressed patients infected with SARS-CoV-2. Proc Natl Acad Sci U S A. 2023;120(3):e2211132120. doi: 10.1073/pnas.2211132120 36623200 PMC9934028

[29] Desikan R, Padmanabhan P, Kierzek AM, van der Graaf PH. Mechanistic Models of COVID-19: Insights into Disease Progression, Vaccines, and Therapeutics. Int J Antimicrob Agents. 2022;60(1):106606. doi: 10.1016/j.ijantimicag.2022.106606 35588969 PMC9110059

[30] Voutouri C, Nikmaneshi MR, Hardin CC, Patel AB, Verma A, Khandekar MJ, et al. In silico dynamics of COVID-19 phenotypes for optimizing clinical management. Proc Natl Acad Sci U S A. 2021;118(3):e2021642118. doi: 10.1073/pnas.2021642118 33402434 PMC7826337

[31] Voutouri C, Hardin CC, Naranbhai V, Nikmaneshi MR, Khandekar MJ, Gainor JF, et al. In silico clinical studies for optimal COVID-19 vaccination schedules in patients with cancer. Cell Rep Med. 2024;5(3):101436. doi: 10.1016/j.xcrm.2024.101436 38508146 PMC10982978

[32] Voutouri C, Hardin CC, Naranbhai V, Nikmaneshi MR, Khandekar MJ, Gainor JF, et al. Dynamic heterogeneity in COVID-19: Insights from a mathematical model. PLoS One. 2024;19(5):e0301780. doi: 10.1371/journal.pone.0301780 38820409 PMC11142552

[33] Harkos C, Stylianopoulos T, Jain RK. Mathematical modeling of intratumoral immunotherapy yields strategies to improve the treatment outcomes. PLoS Comput Biol. 2023;19(12):e1011740. doi: 10.1371/journal.pcbi.1011740 38113269 PMC10763956

[34] Harkos C, Stylianopoulos T. Investigating the synergistic effects of immunotherapy and normalization treatment in modulating tumor microenvironment and enhancing treatment efficacy. J Theor Biol. 2024;583:111768. doi: 10.1016/j.jtbi.2024.111768 38401748

[35] Vavourakis V, Wijeratne PA, Shipley R, Loizidou M, Stylianopoulos T, Hawkes DJ. A Validated Multiscale In-Silico Model for Mechano-sensitive Tumour Angiogenesis and Growth. PLoS Comput Biol. 2017;13(1):e1005259. doi: 10.1371/journal.pcbi.1005259 28125582 PMC5268362

[36] Ben-Akiva E, Karlsson J, Hemmati S, Yu H, Tzeng SY, Pardoll DM, et al. Biodegradable lipophilic polymeric mRNA nanoparticles for ligand-free targeting of splenic dendritic cells for cancer vaccination. Proc Natl Acad Sci U S A. 2023;120(26):e2301606120. doi: 10.1073/pnas.2301606120 37339211 PMC10293809

[37] Li F, Zhang X-Q, Ho W, Tang M, Li Z, Bu L, et al. mRNA lipid nanoparticle-mediated pyroptosis sensitizes immunologically cold tumors to checkpoint immunotherapy. Nat Commun. 2023;14(1):4223. doi: 10.1038/s41467-023-39938-9 37454146 PMC10349854

[38] Malone MK, Smrekar K, Park S, Blakely B, Walter A, Nasta N, et al. Cytokines secreted by stromal cells in TNBC microenvironment as potential targets for cancer therapy. Cancer Biol Ther. 2020;21(6):560–9. doi: 10.1080/15384047.2020.1739484 32213106 PMC7515526

[39] Wang H, Milberg O, Bartelink IH, Vicini P, Wang B, Narwal R, et al. In silico simulation of a clinical trial with anti-CTLA-4 and anti-PD-L1 immunotherapies in metastatic breast cancer using a systems pharmacology model. R Soc Open Sci. 2019;6(5):190366. doi: 10.1098/rsos.190366 31218069 PMC6549962

[40] Eisenhauer EA, Therasse P, Bogaerts J, Schwartz LH, Sargent D, Ford R, et al. New response evaluation criteria in solid tumours: revised RECIST guideline (version 1.1). Eur J Cancer. 2009;45(2):228–47. doi: 10.1016/j.ejca.2008.10.026 19097774

[41] Subudhi S, Voutouri C, Hardin CC, Nikmaneshi MR, Patel AB, Verma A, et al. Strategies to minimize heterogeneity and optimize clinical trials in Acute Respiratory Distress Syndrome (ARDS): Insights from mathematical modelling. EBioMedicine. 2022;75:103809. doi: 10.1016/j.ebiom.2021.103809 35033853 PMC8757652

[42] Gu Z. Complex heatmap visualization. Imeta. 2022;1(3):e43. doi: 10.1002/imt2.43 38868715 PMC10989952

[43] Yagi S, Tsuchikawa K, Tsuji K, editors. A Heatmap-Based Visualization Technique for Finding Operational Problems. 2019 23rd International Conference in Information Visualization–Part II; 2019: IEEE.

[44] Cheng G, Tse J, Jain RK, Munn LL. Micro-environmental mechanical stress controls tumor spheroid size and morphology by suppressing proliferation and inducing apoptosis in cancer cells. PLoS One. 2009;4(2):e4632. doi: 10.1371/journal.pone.0004632 19247489 PMC2645686

[45] Demou ZN. Gene expression profiles in 3D tumor analogs indicate compressive strain differentially enhances metastatic potential. Ann Biomed Eng. 2010;38(11):3509–20. doi: 10.1007/s10439-010-0097-0 20559731

[46] McKee TD, Grandi P, Mok W, Alexandrakis G, Insin N, Zimmer JP, et al. Degradation of fibrillar collagen in a human melanoma xenograft improves the efficacy of an oncolytic herpes simplex virus vector. Cancer Res. 2006;66(5):2509–13. doi: 10.1158/0008-5472.CAN-05-2242 16510565

[47] Nia HT, Liu H, Seano G, Datta M, Jones D, Rahbari N, et al. Solid stress and elastic energy as measures of tumour mechanopathology. Nat Biomed Eng. 2016;1:0004. doi: 10.1038/s41551-016-0004 28966873 PMC5621647

[48] Angeli S, Emblem KE, Due-Tonnessen P, Stylianopoulos T. Towards patient-specific modeling of brain tumor growth and formation of secondary nodes guided by DTI-MRI. Neuroimage Clin. 2018;20:664–73. doi: 10.1016/j.nicl.2018.08.032 30211003 PMC6134360

[49] Li X, Jolly MK, George JT, Pienta KJ, Levine H. Computational Modeling of the Crosstalk Between Macrophage Polarization and Tumor Cell Plasticity in the Tumor Microenvironment. Front Oncol. 2019;9:10. doi: 10.3389/fonc.2019.00010 30729096 PMC6351454

[50] Moffett AS, Deng Y, Levine H. Modeling the Role of Immune Cell Conversion in the Tumor-Immune Microenvironment. Bull Math Biol. 2023;85(10):93. doi: 10.1007/s11538-023-01201-z 37658264 PMC10474003

