# Supplementary material for: Biomarkers of mRNA vaccine efficacy derived from mechanistic modeling of tumor-immune interactions
Source: PLoS Comput Biol. 2025 Jun 12;21(6):e1013163. doi: 10.1371/journal.pcbi.1013163 (PMC12201673; doi:10.1371/journal.pcbi.1013163)
Supplement: S1 Text — Fig A. Flowchart of the Simulation Workflow. This flowchart illustrates the step-by-step process of our simulation framework, starting from initial parameter selection and model setup, followed by execution of simulations, data analysis, and validation with experimental data. Each step ensures a systematic and reproducible methodology for assessing the efficacy of mRNA vaccines and immune checkpoint inhibitors in a computational framework. Fig B. Comparison of Mechanistic Model Predictions with Experimental CD8 + T Cell Data. Comparison of model-predicted CD8 + T cell percentages in tumors with experimental data from two independent studies. This comparison highlights the model’s accuracy in replicating immune response patterns observed in pre-clinical settings. Fig C. (A) Immune Cell Infiltration Patterns in Responders vs. Non-Responders. Scatter plot showing the relationship between cytotoxic T cell and M2 macrophage densities in virtual patients. High T cell and low M2 macrophage densities are linked to favorable vaccine response. (B) Tumor-Type-Specific Response Probabilities. Box plot comparing melanoma and breast cancer subgroups. Melanoma shows higher and more consistent response probabilities, emphasizing tumor-type impact on treatment efficacy. Table A. Model Parameter Values. This table provides a comprehensive list of all model parameters, their descriptions, units, and literature references. Parameters include biological rates, cytokine production, immune cell dynamics, vaccine uptake metrics, and tumor microenvironment properties. Table B. Contribution Scores. (DOCX) [file pcbi.1013163.s001.docx]

**Fig A:** Flowchart of the Simulation Workflow. This flowchart illustrates the step-by-step process of our simulation framework, starting from initial parameter selection and model setup, followed by execution of simulations, data analysis, and validation with experimental data. Each step ensures a systematic and reproducible methodology for assessing the efficacy of mRNA vaccines and immune checkpoint inhibitors in a computational framework.

**
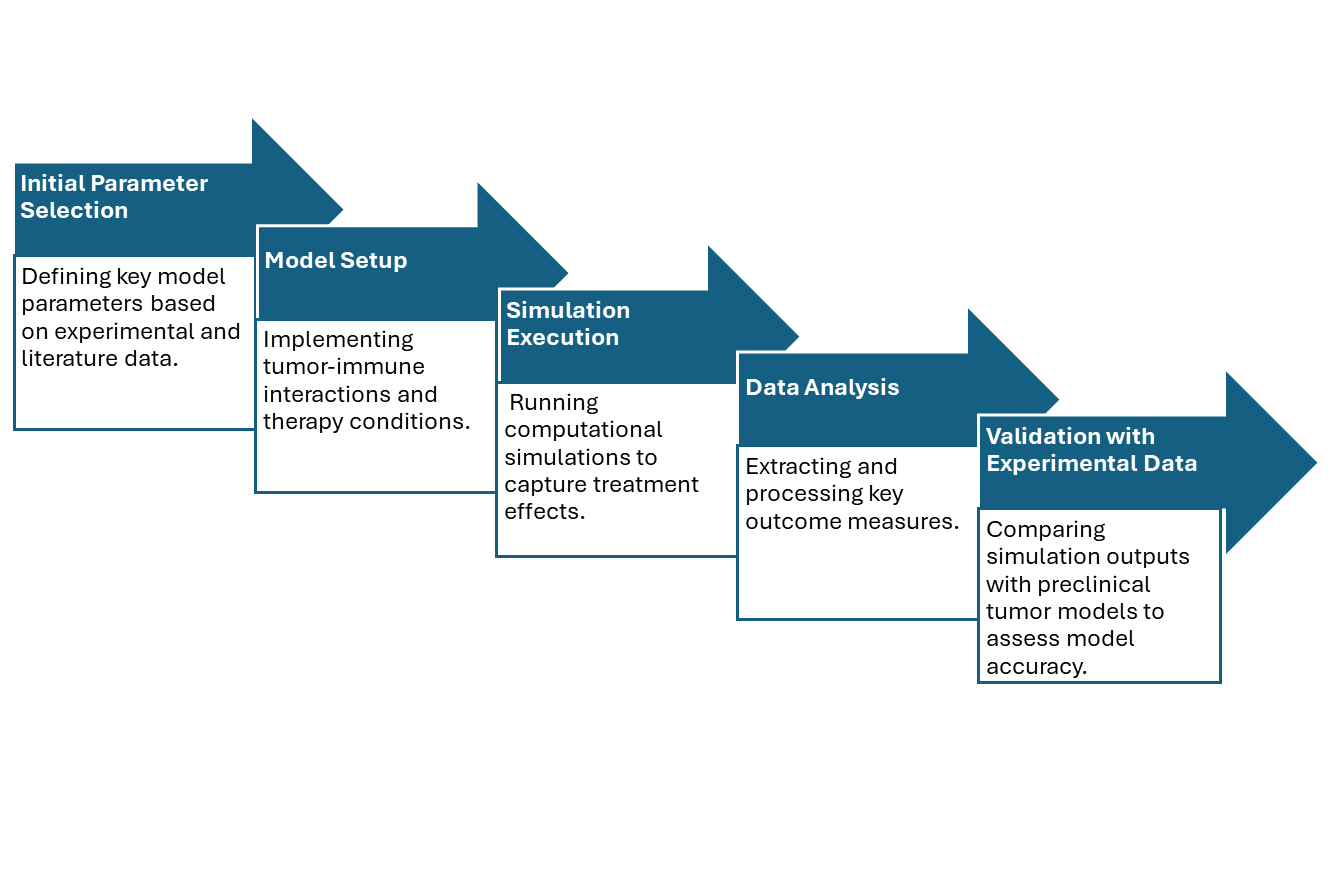
**

**Fig B:** Comparison of mechanistic model predictions of percentage population of CD8+ T cells in tumor with experimental data from two independent studies.


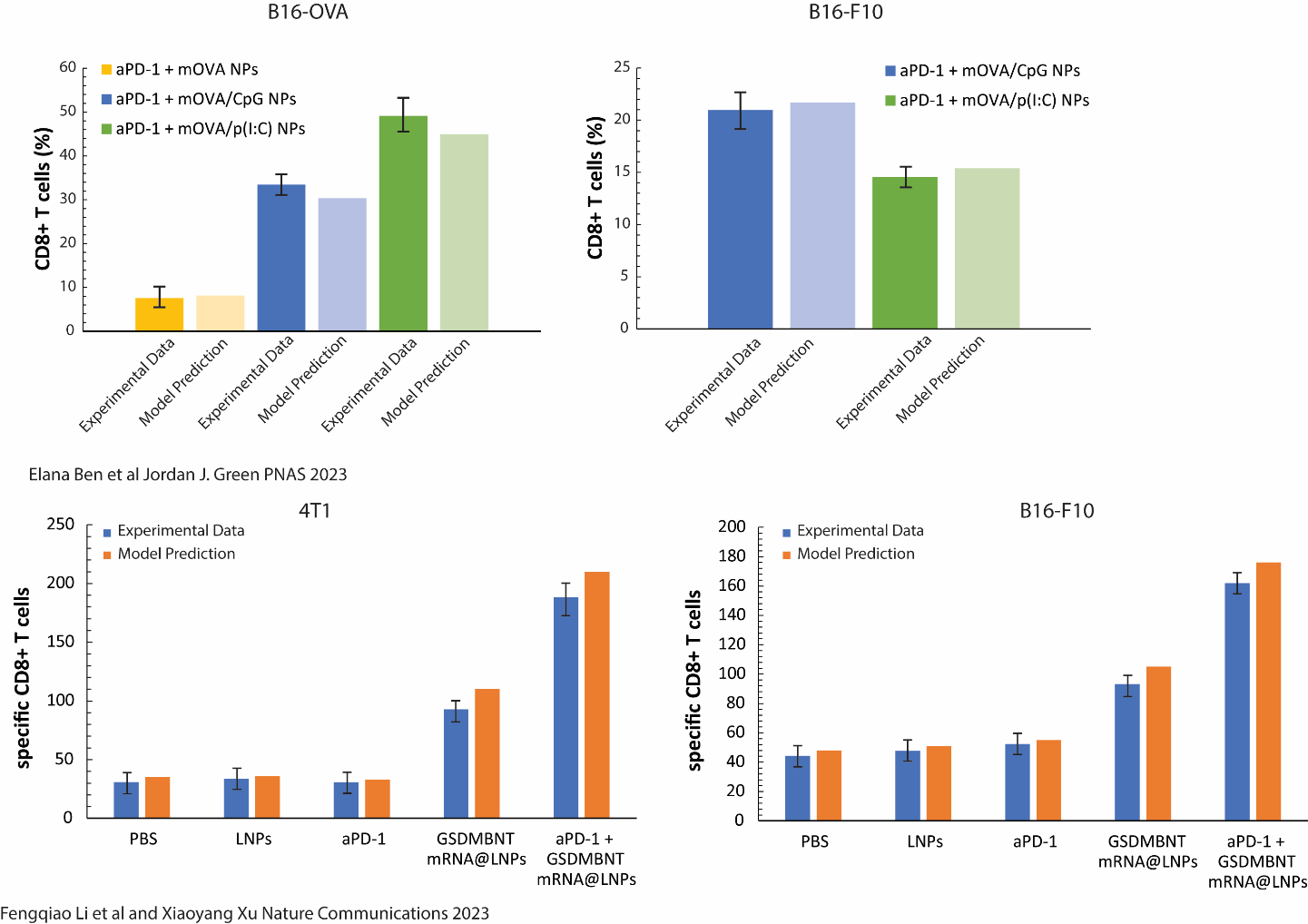


**Fig C:** (A) **Mechanistic Insights into Immune Cell Infiltration with Extra Variation.** Scatter plot showing the relationship between cytotoxic T cell density and M2 macrophage density in responders (blue) and non-responders (red). High cytotoxic T cell density and low M2 macrophage density are associated with favorable responses, while the opposite pattern is observed in non-responders. The plot includes added variation to illustrate the diversity in immune cell infiltration patterns among different patient responses. (B) **Subgroup Analysis by Tumor Type.** Box plot comparing the response probabilities for two tumor types: melanoma and a breast cancer. The melanoma shows a higher median response probability and a narrower interquartile range, indicating more consistent treatment outcomes compared to the breast cancer, which displays lower response probabilities and greater variability. This analysis highlights the influence of tumor type on treatment efficacy.


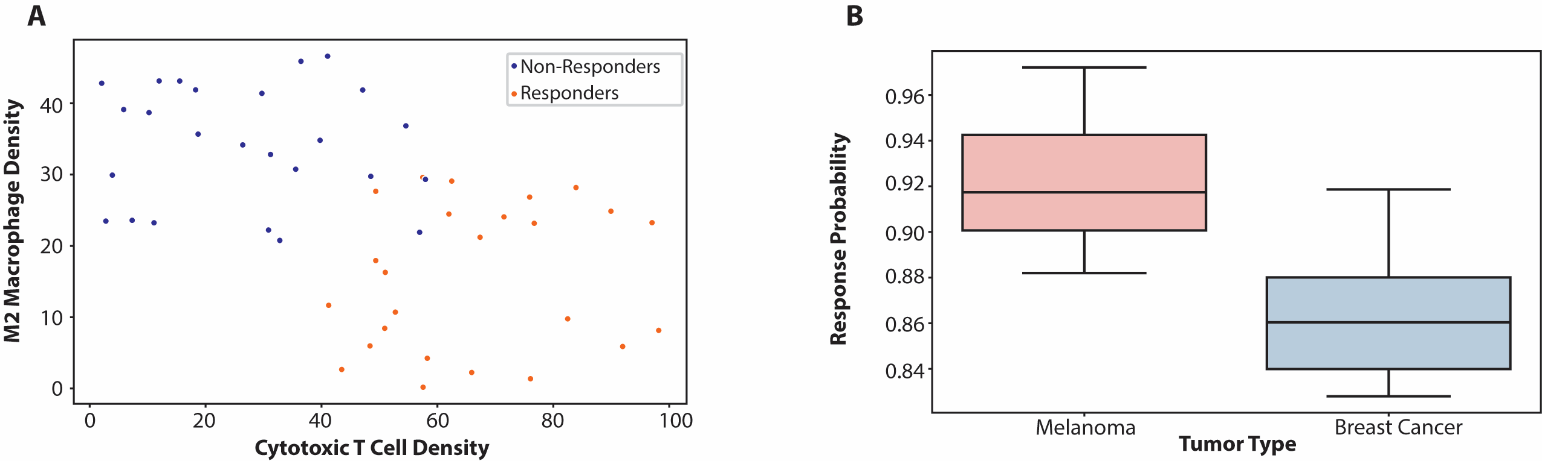


**Equations of the Mathematical Modeling Framework**

1. Host Tissues and Tumor
   1. Host cells

The concentration of cancer cells

| $\frac{\partial\text{ }H_{Hpart}}{\partial t}+\nabla\cdot\left[ -D_{c}*\nabla H_{Hpart}+us*H_{Hpart} \right]=\left[ k_{H}\text{ }H_{Hpart}\text{-}d_{H}\text{ }H_{Hpart}\text{ } \right]\text{ }$ | (1) |
| --- | --- |

Where the first right-hand side term describes the proliferation of the total host cells and the following term describes the degradation of host cells

- 1. Antibody

| $\frac{\partial\text{ }A}{\partial t}+\nabla\cdot\left[ -D_{c}*\nabla A+uf*A \right]=Q_{b-T}A-Q_{T-Tl}\text{ }A\text{ }\text{+(}\pi_{AS}\text{ }P^{S}+\pi_{AL}\text{ }P^{L}-k_{a}\text{ }A\text{ }\text{) }$ | (2) |
| --- | --- |

Where the first right-hand side term describes the production of the antibody by short-lived plasma and the second term describes the production of the antibody by long-lived plasma and the last term describes the degradation of antibody.

- 1. Antigen

| $\frac{\partial\text{ }{Ag}}{\partial t}+\nabla\cdot\left[ -D_{c}*\nabla{Ag}+uf*{Ag} \right]=\text{ }Q_{b-T}Ag-Q_{T-Tl}\text{ }Ag\text{ }\text{+}\left( \begin{aligned} \text{ } \\ \text{ }k_{Ag,NK}\text{ }(NK)\text{ }+k_{Ag,M1}\text{ }(M_{1})\text{ } \\ +\text{ }k_{Ag,Th}\text{ }(Th^{E})\text{ }+\text{ }k_{Ag,N}\text{ }(N)\text{ } \\ -\text{ }k_{Ag,DC}\text{ }(DC^{*}){Ag}\text{+}k_{Ag,Vaprotein}Vprotein\text{ } \\ -\text{ }k_{Ag}\text{ }{Ag}\text{ } \end{aligned} \right)$ | (3) |
| --- | --- |

Where the first right-hand side term describes the production of the antigen by natural killing cells, the second term describes the production of the antigen by macrophages, the third term describes the production of the antigen by neutrophils, the fourth term describes the degradation term of antigen by APC cells, the following term describes the production of the antigen by protein after mRNA transcription after vaccination and the last term describes the degradation of the antigen.

- 1. Cytokine

| $\frac{\partial c}{\partial t}+\nabla\cdot\left[ -D_{c}*\nabla\text{ }c+uf*c \right]=\text{ }Q_{b-T}c-Q_{T-Tl}\text{ }c\text{ }\text{+}k_{c,NK}\text{ }\left( NK \right)\text{ }+\text{ }k_{c,DC}\text{ }\left( DC^{*} \right)\text{ }+\text{ }k_{c,Th}\text{ }\left( Th^{E} \right)\text{ }+\text{ }k_{c,N}\text{ }\left( N \right)\text{ }+\text{ }k_{c,M1}\text{ }\left( M_{1} \right)\text{ }+K_{c-IL6}\text{ }\left[ IL6 \right]\left( K_{c-EC}EC_{Hpart} \right)-k_{c,Treg}\text{ }(T_{reg})\text{ }-d_{s}\text{ }c_{Hpart}\text{ })$ | (4) |
| --- | --- |

Where the first right-hand side term describes the production of the cytokine by natural killing cells, the second term describes the production of the cytokine by APC cells, the third term describes the production of the cytokine by effector T-helper cells (CD4+), the third term describes the production of the cytokine by neutrophils, the fourth term describes the production of the cytokine by macrophages, the following term describes the production of the cytokine by endothelial cells after IL6 bound to the IL6 receptor, the following term describes the degradation of the cytokine by Treg cells, and the last term describes the degradation of the cytokine.

- 1. Endothelial cells of normal part vessels

| $\frac{\partial\text{ }EC_{Hpart}}{\partial t}+\nabla\cdot\left[ -D_{c}*\nabla EC_{Hpart}+us*\text{ }EC_{Hpart} \right]=\left[ k_{ec}\text{ }EC_{Hpart}\text{ }-d_{ec}\text{ }EC_{Hpart} \right]$ | (5) |
| --- | --- |

Where the first right-hand side term describes the proliferation of the total endothelial cells and the following term describes the degradation of endothelial cells

- 1. The reaction rate of the IL-6 reads

| $\frac{\partial\text{ }\left[ IL6 \right]}{\partial t}+\nabla\cdot\left[ -D_{c}*\nabla\left[ IL6 \right]+uf*\text{ }\left[ IL6 \right] \right]=Q_{b-T}\left[ IL6 \right]-Q_{T-Tl}\text{ }\left[ IL6 \right]\text{ }\text{+}\left[ K_{IL6-Nk}Nk+K_{IL6-DC}{DC}^{*}+K_{IL6-TE}\left( {Th}^{E}+T^{E} \right)+K_{IL6-M1}M_{1}-\gamma_{IL6}\left[ IL6 \right] \right]$ | (6) |
| --- | --- |

where the first four terms are the production of IL-6 by the natural killer, activated dendritic, and effector T cells, and the macrophages, respectively. The fifth term is the degradation of the IL-6.

- 1. The reaction rate of the IL-2 reads

| $\frac{\partial\text{ }\left[ IL2 \right]}{\partial t}+\nabla\cdot\left[ -D_{c}*\nabla\left[ IL2 \right]+uf*\text{ }\left[ IL2 \right]*x^{2} \right]=Q_{b-T}\left[ IL2 \right]-Q_{T-Tl}\text{ }\left[ IL2 \right]+\left[ K_{IL2-DC}{DC}^{*}+K_{IL2-TE}\left( {Th}^{E}+T^{E} \right)+K_{IL2-M1}M_{1}-\gamma_{IL2}\left[ IL2 \right] \right]$ | (7) |
| --- | --- |

where the first three terms are the production of IL-2 by the activated dendritic, effector T cells, and the macrophages, respectively. The fourth term is the degradation of the IL-2. T

- 1. The reaction rate of the IL-12 reads

| $\frac{\partial\text{ }\left[ IL12 \right]}{\partial t}+\nabla\cdot\left[ -D_{c}*\nabla\left[ IL12 \right]+uf*\text{ }\left[ IL12 \right] \right]=Q_{b-T}\left[ IL12 \right]-Q_{T-Tl}\text{ }\left[ IL12 \right]+\left[ K_{IL12-N}N+K_{IL12-DC*}{DC}^{*}+K_{IL12-Ma}M_{a}-\gamma_{IL12}\left[ IL12 \right] \right]$ | (8) |
| --- | --- |

where the first three terms are the production of IL-12 by the activated dendritic, effector T cells, and the macrophages, respectively. The fourth term is the degradation of the IL-12.

- 1. The mass balance for VEGF

| $\frac{\partial\left[ VEGF \right]}{\partial t}+\nabla\cdot\left[ -D_{c}*\nabla\left[ VEGF \right]+uf*\text{ }\left[ VEGF \right] \right]=Q_{b-T}\left[ VEGF \right]-Q_{T-Tl}\text{ }\left[ VEGF \right]+\left[ K_{T}G_{a}\left( c_{ox} \right)T+K_{T_{reg}}G_{a}\left( c_{ox} \right)T_{reg}+K_{VEGF}\left[ sIL6R-IL6 \right]-d_{VEGF}\left[ VEGF \right]+\gamma_{VEGF}\left( 100-SPO2 \right) \right]$ | (9) |
| --- | --- |

VEGF is assumed to be produced by cancer cells and *T_regs_* and its production is enhanced under hypoxic conditions as described by the oxygen tension term *G_a._* The following term describes the production of VEGF by IL-6 bound on the soluble IL-6 receptor, the following term is the degradation rate of the VEGF and the last term describes the production of VEGF by hypoxia.

| *G_a_(*$\hat{c_{ox}}$*)=* | *3*$\hat{c_{ox}}$ *for 0<*$\hat{c_{ox}}$*<0.5 (hypoxia)*  *2 -*$\hat{c_{ox}}$ *for 0.5<*$\hat{c_{ox}}$*<1 (normoxia)*  $\hat{c_{ox}}$ *for 1<*$\hat{c_{ox}}$ *(hyperoxia)* |  |
| --- | --- | --- |
|  |  |  |

- 1. Interferon gamma ($\boldsymbol{IF}\boldsymbol{N}_{\boldsymbol{\gamma}}$)

| $\frac{\partial{IFN}_{\gamma}}{\partial t}+\nabla\cdot\left[ -D_{c}*\nabla{IFN}_{\gamma}+uf* {IFN}_{\gamma} \right]=Q_{b-T}{IFN}_{\gamma}-Q_{T-Tl}\text{ }{IFN}_{\gamma}+k_{IFN_{\gamma} NK}NK+k_{IFN_{\gamma} {Th}^{E}}{Th}^{E}+k_{IFN_{\gamma} T^{E}}T^{E}+k_{IFN_{\gamma}}- \varepsilon_{IF} {IFN}_{\gamma}$ | (10) |
| --- | --- |

where the first three terms describe the production rate of the interferon gamma (IFNγ) by the natural killer, effector CD4^+^ T, effector CD8^+^ T, respectively. The last term describes the degradation rate of interferon gamma.

- 1. Interferon gamma ($\boldsymbol{TNFa}$)

| $\frac{\partial{TNF}_{a}}{\partial t}+\nabla\cdot\left[ -D_{c}*\nabla{TNF}_{a}+uf* {TNF}_{a} \right]=Q_{b-T}{TNF}_{a}-Q_{T-Tl}\text{ }{TNF}_{a}+k_{TNF_{a} NK}NK+k_{TNF_{a} {Th}^{E}}{Th}^{E}+k_{TNF_{a} T^{E}}T^{E}+k_{TNF_{a} Ma}Ma+k_{TNF_{a}}- \varepsilon_{IF} {TNF}_{a}$ | (11) |
| --- | --- |

where the first four terms describe the production rate of the TNFa by the natural killer, effector CD4^+^ T, effector CD8^+^ T and macrophages, respectively. The last term describes the degradation rate of interferon gamma.

- 1. Anti-inflammatory cytokines

| $\frac{\partial a}{\partial t}+\nabla\cdot\left[ -D_{c}*\nabla a+uf* a \right]=Q_{b-T}a-Q_{T-Tl}\text{ }a+k_{a-N}N*\left( M_{a}+DC \right)+K_{ant-T_{reg}}T_{reg}+K_{ant-Ma}M_{a}-\gamma_{a}a$ | (12) |
| --- | --- |

where the first term describes the production of anti-inflammatory cytokines by macrophages and dendritic cells after phagocytosis of apoptotic neutrophils. The other tow terms are the production by regulatory T cells and macrophages respectively. The last term is the degradation rate of anti-inflammatory cytokines

- 1. Reactions of PDL-1, PD-1, and anti-PD-1
     1. The reaction of the PDL-1 reads

| $\frac{d[PDL1]}{dt}=\left[ \left( \lambda_{EC}-d_{EC} \right)\left[ EC \right]+\left( \lambda_{T}-d_{T} \right)\hat{T} \right]\frac{\left[ PDL1 \right]}{H+\left[ EC \right]+\hat{T}}+h_{PDL1}T_{E}\left( 1+\frac{{TNF}_{a}}{K_{TNFa}+{TNF}_{a}} \right)+h_{M2}M_{a}$ | (13) |
| --- | --- |

where the first term describes the production(*λ*_i_)/degradation(*d*_i_) of the PD1 ligand by healthy epithelial, healthy endothelial cells and tumor cells, the last two terms describe the production of the PD1 ligand by Effector (Activated) T cells macrophages.

- - 1. The reaction of the PD-1 reads

| $\frac{d\left[ PD1 \right]}{dt}=\left[ \left( \lambda_{TE}-d_{TE} \right)T_{E}+\left( \lambda_{TN}-d_{TN} \right)T_{N}+\left( \lambda_{N}-d_{N} \right)N+\left( \lambda_{NK}-d_{NK} \right)NK+\left( \lambda_{M2}-d_{M2} \right)M_{a} \right]\frac{\left[ PD1 \right]}{T_{E}+T_{N}+N+NK+M_{a}}-\mu_{PD1-aPD1}\left[ PD1 \right]\left[ anti-PD1 \right]$ | (14) |
| --- | --- |

where the first describes the production(λi)/degradation(di) of the PD1 by Effector (Activated) T cells, Naïve T cells, neutrophils, natural killer cells and macrophages. The last term describes the binding of the anti-PD1 to PD1.

- - 1. The reaction of the PD-1 – PDL-1 complex reads

| $\frac{d\left[ PD1-PDL1 \right]}{dt}=a_{PL}\left[ PD1 \right]\left[ PDL1 \right]-d_{Q}\left[ PD1-PDL1 \right]$ | (15) |
| --- | --- |

where the first term describes the binding of PD1 to the PD1 ligand and the last term describes its degradation rate.

- - 1. The reaction of the anti-PD-1 reads

| $\frac{d\left[ anti-PD1 \right]}{dt}=\gamma_{A}-\mu_{PD1-aPD1}\left[ PD1 \right]\left[ anti-PD1 \right]-d_{A}\left[ anti-PD1 \right]$ | (16) |
| --- | --- |
|  |  |

where the first term represents the source term of the anti-PD1, the second term describes the production of the anti-PD1 - PD1 complex and the last term describes its degradation rate.

- 1. Cells of immune system
     1. Neutrophils

The reaction rate of the neutrophils reads

| $\frac{\partial N}{\partial t}+\nabla\cdot\left[ -D_{c}*\nabla N+uf*N \right]=Q_{b-T}N-Q_{T-Tl}\text{ }N+\frac{\chi_{N} c}{1+a}+\chi_{N-IL6}\left[ IL6 \right]-\gamma_{n}N$ | (17) |
| --- | --- |

Where the production rate of the neutrophils depends on cytokines (pro/anti-inflammatory) and IL-6 - IL-6R complex, and the last term describes their death rate.

- - 1. Neutrophils Extracellular Traps (NETs)

$$\frac{\partial[NETs]}{\partial t}+\nabla\cdot\left[ -D_{c}*\nabla[NETs]+uf*[NETs] \right]=Q_{b-T}[NETs]-Q_{T-Tl}[NETs]+\gamma nN*-\gamma_{NETs}[NETs]$$

(18)

where the first term describes the production Neutrophil extracellular traps and the last term is their degradation rate.

- - 1. Immature dendritic cells (DC)

The reaction rate of the immature dendritic cells reads

| $\frac{\partial DC}{\partial t}+\nabla\cdot\left[ -D_{c}*\nabla DC+uf*DC \right]=Q_{b-T}DC-Q_{T-Tl}DC+S_{DC}-d_{DC}DC-\left[ \frac{\chi_{DC} c}{1+a} DC T+\text{ }k_{AgTpart,DC}\text{ }(DC){Ag}_{Tpart}\text{ }V_{Tpart} \right]- k_{Treg-DC} T_{reg} DC$ | (19) |
| --- | --- |

where the first term describes the source term of DC and the second term is the death rate of DC, the rest term describes the conversion of dendritic cells to antigen presenting cells and the last term describes the degradation rate of DC by regulatory T cells.

- - 1. Antigen presenting cells APCs (*DC^*^*)

The reaction rate of the APCs reads

| $\frac{\partial DC^{*}}{\partial t}+\nabla\cdot\left[ -D_{c}*\nabla DC^{*}+uf*DC^{*} \right]=Q_{b-T}DC^{*}-Q_{T-Tl}DC^{*}+\left[ \frac{\chi_{DC} c}{1+a} DC T+\text{ }k_{AgTpart,DC}\text{ }(DC){Ag}_{Tpart}\text{ }V_{Tpart} \right]-k_{Treg-DC^{*}}T_{reg}DC^{*}-\delta_{DC*}{DC}^{*}$ | (20) |
| --- | --- |

where the first two terms describe the conversion rates of dendritic cells to APCs, the rest term describes the degradation rate of APCs by regulatory T cells and the last term describes the death rate of APCs.

- - 1. Naïve CD4^+^ T cells (*Th^N^*)

The reaction rate of the Naive CD4^+^ T cells reads

| $\frac{\partial{Th}^{N}}{\partial t}+\nabla\cdot\left[ -D_{c}*\nabla{Th}^{N}+uf*{Th}^{N} \right]=Q_{b-T}{Th}^{N}-Q_{T-Tl}{Th}^{N}+S_{ThN}-h_{Th}{DC}^{*}{Th}^{N}\left[ w_{ag-CD4}^{i=1-34}\left( \frac{{Ag}_{Tpart}}{1+{Ag}_{Tpart}} \right) \right]\left( \frac{c}{1+c} \right)\left( \frac{{IFN}_{\gamma}}{K_{IF}+{IFN}_{\gamma}} \right)\left( \frac{{[IL12]}}{K_{IL12}+[IL12]} \right) \left( \frac{{[IL2]}}{K_{IL12}+[IL2]} \right)\frac{K_{T}}{K_{T}+\left[ PD1-PDL1 \right]}-d_{TN}{Th}^{N}-\frac{h_{Treg}DC {Th}^{N}a}{\left( K_{Th^{N} DC}+DC \right)\left( K_{DC Th^{N}}+{Th}^{N} \right)\left( K_{T_{reg} a}+a \right)}$ | (21) |
| --- | --- |

where the first term describes the source term of Naïve CD4^+^ T cells, the rest term describes the activation of naive CD4^+^ T cells, the following term describes the death rate of naïve CD4^+^ T cells and the last term describes its transformation to regulatory T cells.

- - 1. Activated CD4^+^ T cells (*Th^E^*)

The reaction rate of the activated CD4^+^ T cells reads

| $\frac{\partial{Th}^{E}}{\partial t}+\nabla\cdot\left[ -D_{c}*\nabla{Th}^{E}+uf*{Th}^{E} \right]=Q_{b-T}{Th}^{E}-Q_{T-Tl}{Th}^{E}+h_{Th} {DC}^{*}{Th}^{N}\left[ w_{ag-CD4}^{i=1-34}\left( \frac{{Ag}_{Tpart}}{1+{Ag}_{Tpart}} \right) \right]\left( \frac{c}{1+c} \right)\left( \frac{{IFN}_{\gamma}}{K_{IF}+{IF}_{\gamma}} \right)\left( \frac{{[IL12]}}{K_{IL12}+[IL12]} \right) \left( \frac{{[IL2]}}{K_{IL12}+[IL2]} \right)\frac{K_{T}}{K_{T}+\left[ PD1-PDL1 \right]}+\rho_{N}{Th}^{E}\left( \frac{As}{1+As} \right)\left( \frac{c}{1+c} \right)\left( \frac{{IFN}_{\gamma}}{K_{IF}+{IFN}_{\gamma}} \right) \frac{K_{T}}{K_{T}+\left[ PD1-PDL1 \right]}{Th}^{E}-\varepsilon{Th}^{E}\frac{\left[ PD1-PDL1 \right]}{AS}-\frac{k_{Treg_{1}}{Th}^{E}T_{reg}}{(1+{Th}^{E})(1+T_{reg})}$ | (22) |
| --- | --- |

where the first term describes the activation rate of naïve CD4^+^ T cells, the second term describes the proliferation rate of effector CD4^+^ T cells, the third term describes the conversion rate of effector CD4^+^ T cells to memory CD4^+^ T cells and the last two terms describe their death rates which depend on the PD1 – PDL1 complex and the regulatory T cells

- - 1. Regulatory T cells (*Treg*)

The reaction rate of the regulatory T cells reads

| $\frac{\partial T_{reg}}{\partial t}+\nabla\cdot\left[ -D_{c}*\nabla T_{reg}+uf*T_{reg} \right]=Q_{b-T}T_{reg}-Q_{T-Tl}T_{reg}+S_{Treg}+\rho_{Treg} T_{reg} \left( \frac{a}{1+c} \right)\left( \frac{{[IL2]}}{K_{IL12}+[IL2]} \right)+\rho_{Treg} T_{reg} TNF_{a}-\pi_{Treg}T_{reg}$ | (23) |
| --- | --- |

where the first term describes the source term. The second term is their proliferation rate due to cytokines (cytokines and anti -inflammatory), the third term describes the proliferation rate and the last term describes their death rate.

- - 1. Naïve CD8^+^ T cells (*T^N^*)

The reaction rate of the naïve CD8^+^ T cells reads

| $\frac{\partial T^{N}}{\partial t}+\nabla\cdot\left[ -D_{c}*\nabla T^{N}+uf*T^{N} \right]=Q_{b-T}T^{N}-Q_{T-Tl}T^{N}+S_{Treg}+S_{TN}-h_{T}{DC}^{*}T^{N}\left[ w_{ag-CD8}^{i=1-34}\left( \frac{{Ag}_{Tpart}}{1+{Ag}_{Tpart}} \right) \right]\left( \frac{c}{1+c} \right)\left( \frac{{IFN}_{\gamma}}{K_{IF}+{IFN}_{\gamma}} \right) \left( \frac{{[IL12]}}{K_{IL12}+[IL12]} \right)\left( \frac{{[IL2]}}{K_{IL12}+[IL2]} \right)\frac{K_{T}}{K_{T}+\left[ PD1-PDL1 \right]}-d_{TN}T^{N}$ | (24) |
| --- | --- |

where the first term describes the source term of Naïve CD8^+^ T cells, the second term describes the activation rate of naïve CD8^+^ T cells, and the last term describes the death rate of naïve CD8^+^ T cells.

- - 1. Activated CD8^+^ T cells (*T^E^*)

The reaction rate of the activated CD8+ T cells reads

| $\frac{\partial T^{E}}{\partial t}+\nabla\cdot\left[ -D_{c}*\nabla T^{E}+uf*T^{E} \right]=Q_{b-T}T^{E}-Q_{T-Tl}T^{E}+h_{T}{DC}^{*}T^{N}\left[ w_{ag-CD8}^{i=1-34}\left( \frac{{Ag}_{Tpart}}{1+{Ag}_{Tpart}} \right)\left( \frac{c}{1+c} \right)\left( \frac{{IFN}_{\gamma}}{K_{IF}+{IFN}_{\gamma}} \right)\left( \frac{\left[ IL12 \right]}{K_{IL12}+\left[ IL12 \right]} \right)\left( \frac{\left[ IL2 \right]}{K_{IL12}+\left[ IL2 \right]} \right) \right] \frac{K_{T}}{K_{T}+\left[ PD1-PDL1 \right]}+\rho_{T}T^{E}\left( \frac{As}{1+As} \right)\left( \frac{c}{1+c} \right)\left( \frac{{IFN}_{\gamma}}{K_{IF}+{IFN}_{\gamma}} \right) \frac{K_{T}}{K_{T}+\left[ PD1-PDL1 \right]}-\pi_{TM}T^{E}$  $-\varepsilon T^{E}\frac{\left[ PD1-PDL1 \right]}{AS}-\frac{k_{Treg_{2}}T^{E}T_{reg}}{\left( 1+T^{E} \right)\left( 1+T_{reg} \right)}-k_{T}T-d_{TE}T^{E}$ | (25) |
| --- | --- |

where the first term describes the activation rate of the naïve CD8^+^ T cells, the second term describes the proliferation rate of the effector CD8^+^ T cells, the third term describes the conversion rate of effector CD8^+^ T cells to memory CD8^+^ T cells and the last terms describe their death rates which depend on the PD1 – PDL1 complex, the regulatory T cells, the tumor cells and death degradation rate.

- - 1. Macrophages

| $\frac{\partial M_{a}}{\partial t}+\nabla\cdot\left[ -D_{c}*\nabla M_{a}+uf*M_{a} \right]=Q_{b-T}M_{a}-Q_{T-Tl}M_{a}+\chi mc+\chi_{Ma-IL6}[IL6]]-\frac{\chi_{DC} c}{1+a}M_{a}T-\gamma aM_{a}$ | (26) |
| --- | --- |

Macrophages are recruited by cytokines and IL-6 which is bound to IL-6R, die with a rate constant γa.

- - 1. Natural killer cells

The reaction rate of the natural killer cells reads

| $\frac{\partial NK}{\partial t}+\nabla\cdot\left[ -D_{c}*\nabla NK+uf*NK \right]=Q_{b-T}NK-Q_{T-Tl}NK+\frac{\chi_{NK} c}{1+a}\left( \frac{{[IL2]}}{K_{IL12}+[IL2]} \right)-\gamma_{NK}NK$ | (27) |
| --- | --- |

The first term is the production of Natural killer cells depending on the cytokines (pro/anti-inflammatory). The last term is their death rate.

- - 1. Naïve Β cells (*Β^N^*) (From Ref. [1])

(28)

$$\frac{\partial B^{N}}{\partial t}+\nabla\cdot\left[ -D_{c}*\nabla B^{N}+uf*B^{N} \right]=Q_{b-T}B^{N}-Q_{T-Tl}B^{N}+S_{B}-h_{T}B^{N}{DC}^{*}{Th}^{E}\left[ w_{ag-B}^{i=1-34}\left( \frac{{Ag}_{Tpart}}{1+{Ag}_{Tpart}} \right) \right]\left( \frac{c}{1+c} \right)\left( \frac{IF}{K_{IF}+IF} \right)\left( \frac{{[IL12]}}{K_{IL12}+[IL12]} \right) \frac{K_{T}}{K_{T}+\left[ PD1-PDL1 \right]} -d_{B}B^{N}$$

where the first term describes the source term of Naïve B cells, the second term describes the activation rate of naıve B cells, and the last term describes the death rate of naıve B cells

- - 1. Activated Β cells (*Β^Α^*) (From Ref. [1])

$$\frac{\partial B^{A}}{\partial t}+\nabla\cdot\left[ -D_{c}*\nabla B^{A}+uf*B^{A} \right]=Q_{b-T}B^{A}-Q_{T-Tl}B^{A}+h_{T}B^{N}{DC}^{*}{Th}^{E}\left[ w_{ag-B}^{i=1-34}\left( \frac{{Ag}_{Tpart}}{1+{Ag}_{Tpart}} \right)\left( \frac{c}{1+c} \right)\left( \frac{IF}{K_{IF}+IF} \right)\left( \frac{{[IL12]}}{K_{IL12}+[IL12]} \right) \right]\frac{K_{T}}{K_{T}+\left[ PD1-PDL1 \right]}+\rho_{B}{Th}^{E}B^{A}-\delta_{BA}B^{A}-\pi_{s}B^{A}-\pi_{L}({Th}^{E}+{Th}^{M})B^{A}$$

(29)

where the first term describes the activation rate of naıve B cells, the second term describes the proliferation rate of activated B cells, the following term describes the clearance rate of activated B cells, the next term describes the differentiation rate of activated B cells into short-lived antibody-secreting plasma cells, and the last term describes the differentiation rate of activated B cells into long-lived antibody-secreting plasma cells.

- - 1. Long-lived plasma (antibody-secreting) B cells (*P^L^*) From Ref. [1])

$$\frac{\partial P^{L}}{\partial t}+\nabla\cdot\left[ -D_{c}*\nabla P^{L}+uf*P^{L} \right]=Q_{b-T}P^{L}-Q_{T-Tl}P^{L}+\pi_{L}({Th}^{E}+{Th}^{M})B^{A}-\delta_{L}P^{L}$$

(30)

where the first term describes the differentiation rate of activated B cells into long-lived antibody-secreting plasma cells by cell-to-cell interactions between effector and memory CD4+ cells (Th^E^ & Th^M^) and activated B cells (B^A^), and the second term describes the death rate of long-lived antibody-secreting plasma cell

- - 1. Short-lived plasma (antibody-secreting) B cells (*P^S^*) (From Ref. [1])

$$\frac{\partial P^{S}}{\partial t}+\nabla\cdot\left[ -D_{c}*\nabla P^{S}+uf*P^{S} \right]=Q_{b-T}P^{S}-Q_{T-Tl}P^{S}+\pi_{S}B^{A}-\delta_{S}P^{S}$$

(31)

where the first term describes the differentiation rate of activated B cells into short-lived antibody-secreting plasma cells, and the last term describes the death rate of short-lived antibody-secreting plasma cells

- - 1. Memory CD4+ T cells (Th^M^)

$$\frac{\partial{Th}^{M}}{\partial t}+\nabla\cdot\left[ -D_{c}*\nabla{Th}^{M}+uf*{Th}^{M} \right]=Q_{b-T}{Th}^{M}-Q_{T-Tl}{Th}^{M}+\pi_{ThM}{Th}^{E}-\delta_{ThM}{Th}^{M}$$

(32)

where the first term describes the conversion rate of effector CD4+ cells to memory CD4+-cell, and the last term describes the clearance rate of memory CD4+ cells

- - 1. Memory CD8+ T cells (T^M^)

$$\frac{\partial T^{M}}{\partial t}+\nabla\cdot\left[ -D_{c}*\nabla T^{M}+uf*T^{M}* \right]=Q_{b-T}T^{M}-Q_{T-Tl}T^{M}+\pi_{TM}T^{E}-\delta_{TM}T^{M}$$

(33)

where the first term describes the conversion rate of effector CD8+ cells to memory CD8+-cell, and the last term describes the clearance rate of memory CD8+ cells

1. Cancer Cells

The mass balance of the tumor cell density is given by a convection-diffusion-reaction equation and it reads

| $\frac{\boldsymbol{\partial}\boldsymbol{T}}{\boldsymbol{\partial t}}+\boldsymbol{\nabla}\cdot\left[ -\boldsymbol{D}_{\boldsymbol{c}}*\boldsymbol{\nabla}\boldsymbol{T}+\boldsymbol{uf}*\boldsymbol{T} \right]=\frac{\boldsymbol{\lambda}_{\boldsymbol{c}}\boldsymbol{c}_{\boldsymbol{ox}}}{\boldsymbol{k}_{\boldsymbol{c}}+\boldsymbol{c}_{\boldsymbol{ox}}}\boldsymbol{T}-\left( \boldsymbol{n}_{\boldsymbol{T}} \boldsymbol{T}^{\boldsymbol{E}}\left( \frac{{[\boldsymbol{IL}\mathbf{2}-\boldsymbol{IL}\mathbf{2}\boldsymbol{R}]}}{\boldsymbol{K}_{\boldsymbol{IL}\mathbf{2}}+[\boldsymbol{IL}\mathbf{2}-\boldsymbol{IL}\mathbf{2}\boldsymbol{R}]} \right)\boldsymbol{T}+\boldsymbol{n}_{\boldsymbol{T}} \boldsymbol{Nk}\left( \frac{{[\boldsymbol{IL}\mathbf{2}-\boldsymbol{IL}\mathbf{2}\boldsymbol{R}]}}{\boldsymbol{K}_{\boldsymbol{IL}\mathbf{2}}+[\boldsymbol{IL}\mathbf{2}-\boldsymbol{IL}\mathbf{2}\boldsymbol{R}]} \right)\boldsymbol{T}+\boldsymbol{n}_{\boldsymbol{M}\mathbf{1}} \boldsymbol{M}_{\mathbf{1}}\boldsymbol{T}+\boldsymbol{n}_{\boldsymbol{T}} \boldsymbol{N}\boldsymbol{T} \right) \frac{\boldsymbol{T}}{\left[ \boldsymbol{PD}\mathbf{1}-\boldsymbol{PDL}\mathbf{1} \right]}-\boldsymbol{d}_{\boldsymbol{Tc}}\boldsymbol{T}$ | (34) |
| --- | --- |

Where the first right-hand side term describes the proliferation of the total cancer cells due to oxygen and the following term describes the killing of cancer cells by activated CD8^+^ cells, natural kiler cells, macrophages and neutrophils, and the last term describes the degradation rate.

- 1. Oxygen Concentration

| ,  (35) |
| --- |

where *c_ox_* is the oxygen concentration, *D_ox_* is the diffusion coefficient of oxygen in the interstitial space, *A_ox_* and *k_ox_* are oxygen uptake parameters, *P_er_* is the vascular permeability of oxygen that describes diffusion across the tumor vessel wall and *C_iox_* is the oxygen concentration in the vessels.

1. Vaccination-induced Immunity [2-5]
   - 1. Free vaccine [adenovirus (DNA) or lipid coat (mRNA)]

$$\frac{\partial Va}{\partial t}+\nabla\cdot\left[ -D_{c}*\nabla Va+uf*Va \right]=S^{Va}+\frac{Q_{Tpart}Va}{V_{Tpart}}-\frac{\left( Q_{Tpart}-L_{Tpart} \right)Va}{V_{Tpart}}-K_{cell-Vaccine}^{on}VaH+K_{cell-Vaccine}^{off}VaH-K_{dVa}Va$$

(36)

where the terms respectively describe convectional transport of free vaccine particles (adenovirus or lipid coat), the binding of the vaccine particles to cells (Host cells and Antigen presenting cells-APCs) membranes, the detachment of bound vaccine particles from cells membranes and the inactivation of the vaccine particles.

- - 1. Bound vaccine [adenovirus (DNA) or lipid coat (mRNA)]

$$\frac{\partial{Va}_{b}}{\partial t}+\nabla\cdot\left[ -D_{c}*\nabla{Va}_{b}+uf*{Va}_{b} \right]=K_{cell-Vaccine}^{on}VaH-K_{cell-Vaccine}^{off}VaH-Kint{Va}_{b}-K_{dVab}{Va}_{b}$$

(37)

where the terms respectively describe the binding/unbinding of the vaccine particles on the cells membrane, the internalization of the bound vaccine particles into the cells and the last term is the degradation of the bound vaccine particles.

- - 1. Internalized vaccine [adenovirus (DNA)]

$$\frac{\partial Vaint}{\partial t}+\nabla\cdot\left[ -D_{c}*\nabla Vaint+uf*Vaint \right]=K_{intVa}{Va}_{b}-d_{Vaint}$$

(38)9

where the first term describes the internalization of the bound vaccine particles and the second term describes the constant degradation of the internalized vaccine particles.

- - 1. DNA Transcription [adenovirus (DNA)]

(39)9

$$\frac{\partial VaTran}{\partial t}+\nabla\cdot\left[ -D_{c}*\nabla VaTran+uf*VaTran \right]=K_{Tran}{Va}_{int}-d_{VaTran}$$

where the first term describes the DNA transcription of the internalized vaccine particles and the second term describes the constant degradation of the translation of the DNA.

- - 1. Production proteins [adenovirus (DNA)]

(40)

$$\frac{\partial Vprotein}{\partial t}+\nabla\cdot\left[ -D_{c}*\nabla Vprotein+uf*Vprotein \right]=K_{protein}{Va}_{Tran}-d_{Vprotein}$$

where the first term describes the production of viral protein or viral antigen from the translation of the mRNA and the last describes the constant degradation of viral proteins.

- - 1. Internalized vaccine [lipid coat (mRNA)]

(41)

$$\frac{\partial Vaint}{\partial t}+\nabla\cdot\left[ -D_{c}*\nabla Vaint+uf*Vaint \right]=K_{intVa}{Va}_{b}-d_{Vaint}$$

where the first term describes the internalization of the bound vaccine particles, the second term describes the constant degradation of the internalized vaccine particles.

- - 1. Production proteins [lipid coat (mRNA)]

(42)

$$\frac{\partial Vprotein}{\partial t}+\nabla\cdot\left[ -D_{c}*\nabla Vprotein+uf*Vprotein \right]=K_{protein}{Va}_{int}-d_{Vprotein}$$

where the first term describes the production of viral protein or viral antigen from the translation of the mRNA and the last describes the constant degradation of viral proteins.

1. Biomechanical model of tumor growth

The growth stretch ratio, *λ_g_* is calculated as a function of the oxygen concentration in the tissue

|    | (43) |
| --- | --- |

where *S^s^* is the creation/degradation of the cancer cells. Where λc and kc are constant parameters derived from experimental data.

- - 1. Darcy's law

The fluid pressure *p* is calculated by taking the sum of the mass conservation equations, taking into account that the sum of the volume fractions of the two phases should equal unity and incorporating Darcy's law for the calculation of the fluid velocity. It yields:

| $-k^{is}\nabla^{2}p+\nabla\cdot\mathbf{v}^{s}=Q+S^{s}$ $Q=P_{erc}S_{v}\left(p_{v}-p\right)-L_{pc}S_{v}^{lv}\left(p-p_{l}\right)$   | (44) |
| --- | --- |

where *p_v_*, $P_{erc}$ and *S_v_* are the micro-vascular pressure, hydraulic conductivity and vascular density of the blood vessels, respectively, and *p_l_*, $L_{pc}$ and $S_{v}^{lv}$ are the corresponding quantities for the lymphatics.

1. Blood compartment
   1. Pro-inflammatory cytokines

| $\frac{dc_{b}}{dt}= \left(\begin{matrix} Q_{_{Gl-b}} c_{Gl}-Q_{b-T} c_{b} \\ + k_{c,NK} (NK) V_{b}+ k_{c,\mathrm{DC}} (\mathrm{DC}^{*}) V_{b} \\ + k_{c,\mathrm{Th}} (Th^{E}) V_{b}+ k_{c,N} (N) V_{b} \\ + k_{c,\mathrm{M}1} (M_{1}) V_{b}+ \\ +K_{c-IL6} [IL6](K_{c-EC}EC_{b}+K_{c-H}H_{b})V_{b} \\ - k_{c,\mathrm{Treg}} (T_{reg}) V_{b}-d_{s} c_{b} V_{b} \end{matrix}\right)/V_{b}$ | (45) |
| --- | --- |

- 1. Anti-inflammatory cytokines

| $\frac{da_{b}}{dt}= \left(\begin{matrix} Q_{_{Gl-b}} a_{Gl}-Q_{b-T} a_{b} \\ + k_{\mathrm{a},N} N(\mathrm{M}+\mathrm{DC}) V_{b}+ k_{\mathrm{a},Treg} (Treg) V_{b} \\ + k_{\mathrm{a},\mathrm{M}} (M) V_{b}-d_{a} a_{b} V_{b} \end{matrix}\right)/V_{b}$ | (46) |
| --- | --- |

- 1. Antibody

| $\frac{dA_{b}}{dt}= \left(\begin{matrix} Q_{_{Gl-b}} A_{Gl}-Q_{b-T} A_{b} \\ + \pi_{AS}P^{S}+ \pi_{AL}P^{L}-d_{a}A \end{matrix}\right)/V_{b}$ | (47) |
| --- | --- |

- 1. Antigen

| $\frac{dAg_{b}}{dt}= \left(\begin{matrix} Q_{_{Gl-b}} Ag_{Gl}-Q_{b-T} Ag_{b} \\ + k_{\mathrm{Ag},NK} (NK) V+ k_{\mathrm{Ag},\mathrm{M}} (M) V \\ + k_{\mathrm{Ag},\mathrm{Th}} (\mathrm{Th}^{E}) V+ k_{\mathrm{Ag},\mathrm{N}} (N) V \\ + k_{\mathrm{Ag},\mathrm{VaProtein}} Va_{protein} V \\ - k_{\mathrm{Ag},\mathrm{DC}} (DC^{*})\mathrm{Ag} V- k_{\mathrm{Ag}} Ag V \end{matrix}\right)/V_{b}$ | (48) |
| --- | --- |

- 1. The reaction rate of the IL-6 reads

| $\frac{dIL6_{b}}{dt}= \left(\begin{matrix} Q_{_{Gl-b}} IL6_{Gl}-Q_{b-T} IL6_{b} \\ + k_{\mathrm{IL}6,NK} (NK) V_{b}+ k_{\mathrm{IL}6,\mathrm{M}} (M) V_{b} \\ + k_{\mathrm{IL}6,\mathrm{T}} (\mathrm{Th}^{E}+\mathrm{T}^{E}) V+ k_{\mathrm{IL}6,DC} (DC^{*}) V_{b} \\ - k_{IL6} IL6 _{b}V_{b} \end{matrix}\right)/V_{b}$ | (49) |
| --- | --- |

- 1. The reaction rate of the IL-2 reads

| $\frac{dIL2_{b}}{dt}= \left(\begin{matrix} Q_{_{Gl-b}} IL2_{Gl}-Q_{b-T} IL2_{b} \\ + k_{\mathrm{IL}2,\mathrm{M}} (M) V_{b} \\ + k_{\mathrm{IL}2,\mathrm{T}} (\mathrm{Th}^{E}+\mathrm{T}^{E}) V+ k_{\mathrm{IL}2,DC} (DC^{*}) V_{b} \\ - k_{IL2} IL2 _{b}V_{b} \end{matrix}\right)/V_{b}$ | (50) |
| --- | --- |

- 1. The reaction rate of the IL-12 reads

| $\frac{dIL12_{b}}{dt}= \left(\begin{matrix} Q_{_{Gl-b}} IL12_{Gl}-Q_{b-T} IL12_{b} \\ + k_{\mathrm{IL}12,\mathrm{N}} (N) V_{b} \\ + k_{\mathrm{IL}12,M} (M) V+ k_{\mathrm{IL}12,DC} (DC^{*}) V_{b} \\ - k_{IL12} IL12 _{b}V_{b} \end{matrix}\right)/V_{b}$ | (51) |
| --- | --- |

- 1. The reaction rate of the VEGF reads

| $\frac{dVEGF_{b}}{dt}= \left(\begin{matrix} Q_{_{Gl-b}} VEGF_{Gl}-Q_{b-T} VEGF_{b} \\ + k_{\mathrm{VEGF},\mathrm{Treg}} (T_{reg}) V_{b} \\ - k_{VEGF} VEGF_{b}V_{b} \end{matrix}\right)/V_{b}$ | (52) |
| --- | --- |

- 1. Interferon gamma ($\boldsymbol{IF}\boldsymbol{N}_{\boldsymbol{\gamma}}$)

| $\frac{dIFN\gamma_{b}}{dt}= \left(\begin{matrix} Q_{_{Gl-b}} IFN\gamma_{Gl}-Q_{b-T} IFN\gamma_{b} \\ + k_{IFN\gamma,NK} (NK) V_{b}+ k_{IFN\gamma,\mathrm{Th}} (\mathrm{Th}^{\mathrm{E}}) V_{b} \\ + k_{IFN\gamma,\mathrm{T}} (T^{E}) V_{b}- k_{IFN\gamma} IFN\gamma_{b}V_{b} \end{matrix}\right)/V_{b}$ | (53) |
| --- | --- |

- 1. Interferon gamma ($\boldsymbol{TNF}_{\boldsymbol{a}}$)

| $\frac{dTNFa_{b}}{dt}= \left(\begin{matrix} Q_{_{Gl-b}} TNFa_{Gl}-Q_{b-T} TNFa_{b} \\ + k_{TNFa,NK} (NK) V_{b}+ k_{TNFa,\mathrm{Th}} (\mathrm{Th}^{\mathrm{E}}) V_{b} \\ + k_{TNFa,\mathrm{T}} (T^{E}) V_{b}+ k_{TNFa,\mathrm{M}} (M) V_{b} \\ - k_{TNFa} TNFa_{b}V_{b} \end{matrix}\right)/V_{b}$ | (54) |
| --- | --- |

- 1. Neutrophils

| $\frac{dN_{b}}{dt}= \left(\begin{matrix} Q_{_{Gl-b}} N_{Gl}-Q_{b-T} N_{b} \\ - k_{N} N_{b}V_{b} \end{matrix}\right)/V_{b}$ | (55) |
| --- | --- |

- 1. Neutrophils Extracellular Traps (NETs)

| $\frac{dNETs_{b}}{dt}= \left(\begin{matrix} Q_{_{Gl-b}} NETs_{Gl}-Q_{b-T} NETs_{b} \\ -d_{NETs}NETsV_{b} \end{matrix}\right)/V_{b}$ | (56) |
| --- | --- |

- 1. Immature dendritic cells (DC)

| $\frac{dDC_{b}}{dt}= \left(\begin{matrix} Q_{_{Gl-b}} DC_{Gl}-Q_{b-T} DC_{b} \\ +S_{DC}-d_{DC}DC_{b}V_{b} \end{matrix}\right)/V_{b}$ | (57) |
| --- | --- |

- 1. Naïve CD4^+^ T cells (*Th^N^*)

| $\frac{dTh^{N}_{b}}{dt}= \left(\begin{matrix} Q_{_{Gl-b}} Th^{N}_{Gl}-Q_{b-T} Th^{N}_{b} \\ -d_{Th^{N}}Th^{N}_{b}V_{b} \end{matrix}\right)/V_{b}$ | (58) |
| --- | --- |

- 1. Effector CD4^+^ T cells (*Th^E^*)

| $\frac{dTh^{E}_{b}}{dt}= \left(\begin{matrix} Q_{_{Gl-b}} Th^{E}_{Gl}-Q_{b-T} Th^{E}_{b} \\ -d_{Th^{E}}Th^{E}_{b}V_{b} \end{matrix}\right)/V_{b}$ | (59) |
| --- | --- |

- 1. Memory CD4^+^ T cells (*Th^M^*)

| $\frac{dTh^{M}_{b}}{dt}= \left(\begin{matrix} Q_{_{Gl-b}} Th^{M}_{Gl}-Q_{b-T} Th^{M}_{b} \\ -d_{Th^{M}}Th^{M}_{b}V_{b} \end{matrix}\right)/V_{b}$ | (60) |
| --- | --- |

- 1. Naïve CD8^+^ T cells (*T^N^*)

| $\frac{dT^{N}_{b}}{dt}= \left(\begin{matrix} Q_{_{Gl-b}} T^{N}_{Gl}-Q_{b-T} T^{N}_{b} \\ -d_{T^{N}}T^{N}_{b}V_{b} \end{matrix}\right)/V_{b}$ | (61) |
| --- | --- |

- 1. Effector CD8^+^ T cells (*T^E^*)

| $\frac{dT^{E}_{b}}{dt}= \left(\begin{matrix} Q_{_{Gl-b}} T^{E}_{Gl}-Q_{b-T} T^{E}_{b} \\ -d_{T^{E}}T^{E}_{b}V_{b} \end{matrix}\right)/V_{b}$ | (62) |
| --- | --- |

- 1. Memory CD8^+^ T cells (*T^M^*)

| $\frac{dT^{M}_{b}}{dt}= \left(\begin{matrix} Q_{_{Gl-b}} T^{M}_{Gl}-Q_{b-T} T^{M}_{b} \\ -d_{T^{M}}T^{M}_{b}V_{b} \end{matrix}\right)/V_{b}$ | (63) |
| --- | --- |

- 1. Regulatory T cells (*Treg*)

| $\frac{dTreg_{b}}{dt}= \left(\begin{matrix} Q_{_{Gl-b}} Treg_{Gl}-Q_{b-T} Treg_{b} \\ -d_{Treg}Treg_{b}V_{b} \end{matrix}\right)/V_{b}$ | (64) |
| --- | --- |

- 1. Macrophages

| $\frac{dM_{b}}{dt}= \left(\begin{matrix} Q_{_{Gl-b}} M_{Gl}-Q_{b-T} M_{b} \\ -d_{M}M_{b}V_{b} \end{matrix}\right)/V_{b}$ | (65) |
| --- | --- |

- 1. Natural killer cells

| $\frac{dNK_{b}}{dt}= \left(\begin{matrix} Q_{_{Gl-b}} NK_{Gl}-Q_{b-T}NK_{b} \\ -d_{NK}NK_{b}V_{b} \end{matrix}\right)/V_{b}$ | (66) |
| --- | --- |

- 1. Naïve B cells (*B^N^*)

| $\frac{dB^{N}_{b}}{dt}= \left(\begin{matrix} Q_{_{Gl-b}} B^{N}_{Gl}-Q_{b-T}B^{N}_{b} \\ -d_{B^{N}}B^{N}_{b}V_{b} \end{matrix}\right)/V_{b}$ | (67) |
| --- | --- |

- 1. Activated B cells (*B^N^*)

| $\frac{dB^{A}_{b}}{dt}= \left(\begin{matrix} Q_{_{Gl-b}} B^{A}_{Gl}-Q_{b-T}B^{A}_{b} \\ -d_{B^{A}}B^{A}_{b}V_{b} \end{matrix}\right)/V_{b}$ | (68) |
| --- | --- |

- 1. Long-lived plasma (antibody-secreting) B cells (*P^L^*)

| $\frac{dP^{L}_{b}}{dt}= \left(\begin{matrix} Q_{_{Gl-b}}P^{L}_{Gl}-Q_{b-T}P^{L}_{b} \\ -d_{P^{L}}P^{L}_{b}V_{b} \end{matrix}\right)/V_{b}$ | (69) |
| --- | --- |

- 1. Short-lived plasma (antibody-secreting) B cells (*P^S^*)

| $\frac{dP^{S}_{b}}{dt}= \left(\begin{matrix} Q_{_{Gl-b}}P^{S}_{Gl}-Q_{b-T}P^{S}_{b} \\ -d_{P^{S}}P^{S}_{b}V_{b} \end{matrix}\right)/V_{b}$ | (70) |
| --- | --- |

1. General lymph nodes
   1. Pro-inflammatory cytokines

| $\frac{dc_{Gl}}{dt}= \left(\begin{matrix} -Q_{_{Gl-b}} c_{Gl}+Q_{Tl-Gl} c_{Tl} \\ + k_{c,NK} (NK) V_{Gl}+ k_{c,\mathrm{DC}} (\mathrm{DC}^{*}) V_{Gl} \\ + k_{c,\mathrm{Th}} (Th^{E}) V_{Gl}+ k_{c,N} (N) V_{Gl} \\ + k_{c,\mathrm{M}1} (M_{1}) V_{Gl}+ \\ +K_{c-IL6} [IL6](K_{c-EC}EC_{Gl}+K_{c-H}H_{Gl})V_{Gl} \\ - k_{c,\mathrm{Treg}} (T_{reg}) V_{Gl}-d_{s} c_{Gl} V_{Gl} \end{matrix}\right)/V_{b}$ | (71) |
| --- | --- |

- 1. Anti-inflammatory cytokines

| $\frac{da_{Gl}}{dt}= \left(\begin{matrix} -Q_{_{Gl-b}} a_{Gl}+Q_{Tl-Gl} a_{Tl} \\ + k_{\mathrm{a},N} N(\mathrm{M}+\mathrm{DC}) V_{Gl}+ k_{\mathrm{a},Treg} (Treg) V_{Gl} \\ + k_{\mathrm{a},\mathrm{M}} (M) V_{Gl}-d_{a} a_{Gl} V_{Gl} \end{matrix}\right)/V_{Gl}$ | (72) |
| --- | --- |

- 1. Antibody

| $\frac{dA_{Gl}}{dt}= \left(\begin{matrix} -Q_{_{Gl-b}} A_{Gl}+Q_{Tl-Gl} A_{Tl} \\ + \pi_{AS}P^{S}_{Gl}+ \pi_{AL}P^{L}_{Gl}-d_{a}A_{Gl} \end{matrix}\right)/V_{Gl}$ | (73) |
| --- | --- |

- 1. Antigen

|  | (74) |
| --- | --- |

- 1. The reaction rate of the IL-6 reads

| $\frac{dIL6_{Gl}}{dt}= \left(\begin{matrix} -Q_{_{Gl-b}} IL6_{Gl}+Q_{Tl-Gl} IL6_{Tl} \\ + k_{\mathrm{IL}6,NK} (NK_{Gl}) V_{Gl}+ k_{\mathrm{IL}6,\mathrm{M}} (M_{Gl}) V_{Gl} \\ + k_{\mathrm{IL}6,\mathrm{T}} (\mathrm{Th}^{E}_{Gl}+\mathrm{T}^{E}_{Gl}) V_{Gl}+ k_{\mathrm{IL}6,DC} (DC^{*}) V_{Gl} \\ - k_{IL6} IL6 _{Gl}V_{Gl} \end{matrix}\right)/V_{Gl}$ | (75) |
| --- | --- |

- 1. The reaction rate of the IL-2 reads

| $\frac{dIL2_{Gl}}{dt}= \left(\begin{matrix} -Q_{_{Gl-b}} IL2_{Gl}+Q_{Tl-Gl} IL2_{Tl} \\ + k_{\mathrm{IL}2,\mathrm{M}} (M_{Gl}) V_{Gl} \\ + k_{\mathrm{IL}2,\mathrm{T}} (\mathrm{Th}^{E}_{Gl}+\mathrm{T}^{E}_{Gl}) V+ k_{\mathrm{IL}2,DC} (DC^{*}_{Gl}) V_{Gl} \\ - k_{IL2} IL2_{Gl}V_{Gl} \end{matrix}\right)/V_{Gl}$ | (76) |
| --- | --- |

- 1. The reaction rate of the IL-12 reads

| $\frac{dIL12_{Gl}}{dt}= \left(\begin{matrix} -Q_{_{Gl-b}} IL12_{Gl}+Q_{Tl-Gl} IL12_{Tl} \\ + k_{\mathrm{IL}12,\mathrm{N}} (N_{Gl}) V_{Gl} \\ + k_{\mathrm{IL}12,M} (M_{Gl}) V_{Gl}+ k_{\mathrm{IL}12,DC} (DC^{*}) V_{Gl} \\ - k_{IL12} IL12_{Gl}V_{Gl} \end{matrix}\right)/V_{Gl}$ | (77) |
| --- | --- |

- 1. The reaction rate of the VEGF reads

|  | (78) |
| --- | --- |

- 1. Interferon gamma ($\boldsymbol{IF}\boldsymbol{N}_{\boldsymbol{\gamma}}$)

| $\frac{dIFN\gamma_{Gl}}{dt}= \left(\begin{matrix} -Q_{_{Gl-b}} IFN\gamma_{Gl}+Q_{Tl-GL} IFN\gamma_{Tl} \\ + k_{IFN\gamma,NK} (NK_{Gl}) V_{Gl}+ k_{IFN\gamma,\mathrm{Th}} (\mathrm{Th}^{\mathrm{E}}_{Gl}) V_{Gl} \\ + k_{IFN\gamma,\mathrm{T}} (T^{E}_{Gl}) V_{Gl}- k_{IFN\gamma} IFN\gamma_{Gl}V_{Gl} \end{matrix}\right)/V_{Gl}$ | (79) |
| --- | --- |

- 1. Interferon gamma ($\boldsymbol{TNF}_{\boldsymbol{a}}$)

| $\frac{dTNFa_{Gl}}{dt}= \left(\begin{matrix} -Q_{_{Gl-b}} TNFa_{Gl}+Q_{Tl-Gl} TNFa_{Tl} \\ + k_{TNFa,NK} (NK_{Gl}) V_{Gl}+ k_{TNFa,\mathrm{Th}} (\mathrm{Th}^{\mathrm{E}}_{Gl}) V_{Gl} \\ + k_{TNFa,\mathrm{T}} (T^{E}_{Gl}) V_{Gl}+ k_{TNFa,\mathrm{M}} (M_{Gl}) V_{Gl} \\ - k_{TNFa} TNFa_{Gl}V_{Gl} \end{matrix}\right)/V_{Gl}$ | (80) |
| --- | --- |

- 1. Neutrophils

| $\frac{dN_{Gl}}{dt}= \left(\begin{matrix} -Q_{_{Gl-b}} N_{Gl}+Q_{Tl-Gl} N_{Tl} \\ + k_{\mathrm{N}} \frac{c_{Gl}}{1+a_{Gl}}+ k_{\mathrm{N},\mathrm{IL}6} (IL6_{Gl}) V_{Gl} \\ - k_{N} N_{Gl}V_{Gl} \end{matrix}\right)/V_{Gl}$ | (81) |
| --- | --- |

- 1. Neutrophils Extracellular Traps (NETs)

| $\frac{dNETs_{Gl}}{dt}= \left(\begin{matrix} -Q_{_{Gl-b}} NETs_{Gl}+Q_{Tl-Gl} NETs_{Tl} \\ + k_{N}N_{Gl}V_{Gl}-d_{NETs}NETs_{Gl}V_{Gl} \end{matrix}\right)/V_{Gl}$ | (82) |
| --- | --- |

- 1. Immature dendritic cells (DC)

| $\frac{dDC_{Gl}}{dt}= \left(\begin{matrix} -Q_{_{Gl-b}} DC_{Gl}+Q_{Tl-Gl} DC_{Tl} \\ +S_{DC}-[x_{DC}\frac{c_{Gl}}{1+a_{Gl}}DC_{Gl}+k_{Ag,DC}DC_{Gl}Ag_{Gl}]V_{Gl} \\ -k_{Ag,Terg}Treg_{Gl}DC_{Gl}V_{Gl}-d_{DC}DC_{Gl}V_{Gl} \end{matrix}\right)/V_{Gl}$ | (83) |
| --- | --- |

- 1. Antigen presenting cells APCs (DC^*^)

| $\frac{dDC^{*}_{Gl}}{dt}= \left(\begin{matrix} Q_{Tl-Gl} DC^{*}_{Tl} \\ +S_{DC^{*}}+[x_{DC}\frac{c_{Gl}}{1+a_{Gl}}DC_{Gl}+k_{Ag,DC}DC_{Gl}Ag_{Gl}]V_{Gl} \\ -k_{Ag,Terg}Treg_{Gl}DC^{*}_{Gl}V_{Gl}-d_{DC}DC^{*}_{Gl}V_{Gl} \end{matrix}\right)/V_{Gl}$ | (84) |
| --- | --- |

- 1. Naïve CD4^+^ T cells (*Th^N^*)

| $\frac{dTh^{N}_{Gl}}{dt}= \left(\begin{matrix} -Q_{_{Gl-b}} Th^{N}_{Gl}+Q_{Tl-Gl} Th^{N}_{Tl} \\ -h_{Th}DC^{*}Th^{N}_{Gl}\left[\begin{matrix} w_{Ag-CD4}^{i=1-34}\left(\frac{Ag_{Gl}}{1+Ag_{Gl}}\right)\left(\frac{c_{Gl}}{1+c_{Gl}}\right)\left(\frac{IFN_{\gamma Gl}}{1+IFN_{\gamma Gl}}\right) \\ \left(\frac{IL12_{Gl}}{1+IL12_{Gl}}\right)\left(\frac{IL2_{Gl}}{1+IL2_{Gl}}\right)\left(\frac{K_{T}}{K_{T}+PD1_{Gl}}\right) \end{matrix}\right] \\ -\left(\frac{h_{Treg}DC_{Gl}Th^{N}_{Gl}a_{Gl}}{(K_{Th,DC}+DC_{Gl})(K_{DC,Th}+Th^{N}_{Gl})(K_{Treg,a}+a_{Gl})}\right)-d_{Th^{N}}Th^{N}_{Gl}V_{Gl} \end{matrix}\right)/V_{b}$ | (85) |
| --- | --- |

- 1. Effector CD4^+^ T cells (*Th^E^*)

| $\frac{dTh^{E}_{Gl}}{dt}= \left(\begin{matrix} -Q_{_{Gl-b}} Th^{E}_{Gl}-Q_{Tl-Gl} Th^{E}_{Tl} \\ +h_{Th}DC^{*}Th^{N}_{Gl}\left[\begin{matrix} w_{Ag-CD4}^{i=1-34}\left(\frac{Ag_{Gl}}{1+Ag_{Gl}}\right)\left(\frac{c_{Gl}}{1+c_{Gl}}\right)\left(\frac{IFN_{\gamma Gl}}{1+IFN_{\gamma Gl}}\right) \\ \left(\frac{IL12_{Gl}}{1+IL12_{Gl}}\right)\left(\frac{IL2_{Gl}}{1+IL2_{Gl}}\right)\left(\frac{K_{T}}{K_{T}+PD1_{Gl}}\right) \end{matrix}\right] \\ +\rho_{Th^{E}}Th^{E}\left[\begin{matrix} \left(\frac{c_{Gl}}{1+c_{Gl}}\right)\left(\frac{IFN_{\gamma Gl}}{1+IFN_{\gamma Gl}}\right)\left(\frac{IL12_{Gl}}{1+IL12_{Gl}}\right) \\ \left(\frac{IL2_{Gl}}{1+IL2_{Gl}}\right)\left(\frac{K_{T}}{K_{T}+PD1_{Gl}}\right) \end{matrix}\right] \\ -\left(\frac{k_{Treg}Th^{E}_{Gl}Treg_{Gl}}{(1+Th^{E}_{Gl})(1+Treg_{Gl})}\right)-\pi_{Th^{M}}Th^{E}_{Gl}V_{Gl}-d_{Th^{E}}Th^{E}_{b}V_{b} \end{matrix}\right)/V_{Gl}$ | (86) |
| --- | --- |

- 1. Memory CD4^+^ T cells (*Th^M^*)

| $\frac{dTh^{M}_{Gl}}{dt}= \left(\begin{matrix} -Q_{_{Gl-b}} Th^{M}_{Gl}-Q_{Tl-Gl} Th^{M}_{Tl} \\ +\pi_{Th^{M}}Th^{E}_{Gl}V_{Gl} \\ -d_{Th^{M}}Th^{M}_{Gl}V_{Gl} \end{matrix}\right)/V_{Gl}$ | (87) |
| --- | --- |

- 1. Naïve CD8^+^ T cells (*T^N^*)

| $\frac{dT^{N}_{Gl}}{dt}= \left(\begin{matrix} -Q_{_{Gl-b}} T^{N}_{Gl}+Q_{Tl-Gl} T^{N}_{Tl} \\ -h_{T}DC^{*}T^{N}_{Gl}\left[\begin{matrix} w_{Ag-CD8}^{i=1-34}\left(\frac{Ag_{Gl}}{1+Ag_{Gl}}\right)\left(\frac{c_{Gl}}{1+c_{Gl}}\right)\left(\frac{IFN_{\gamma Gl}}{1+IFN_{\gamma Gl}}\right) \\ \left(\frac{IL12_{Gl}}{1+IL12_{Gl}}\right)\left(\frac{IL2_{Gl}}{1+IL2_{Gl}}\right)\left(\frac{K_{T}}{K_{T}+PD1_{Gl}}\right) \end{matrix}\right] \\ -d_{T^{N}}T^{N}_{Gl}V_{Gl} \end{matrix}\right)/V_{Gl}$ | (88) |
| --- | --- |

- 1. Effector CD8^+^ T cells (*T^E^*)

| $\frac{dT^{E}_{Gl}}{dt}= \left(\begin{matrix} -Q_{_{Gl-b}} T^{E}_{Gl}+Q_{Tl-Gl} T^{E}_{Tl} \\ +h_{T}DC^{*}T^{N}_{Gl}\left[\begin{matrix} w_{Ag-CD8}^{i=1-34}\left(\frac{Ag_{Gl}}{1+Ag_{Gl}}\right)\left(\frac{c_{Gl}}{1+c_{Gl}}\right)\left(\frac{IFN_{\gamma Gl}}{1+IFN_{\gamma Gl}}\right) \\ \left(\frac{IL12_{Gl}}{1+IL12_{Gl}}\right)\left(\frac{IL2_{Gl}}{1+IL2_{Gl}}\right)\left(\frac{K_{T}}{K_{T}+PD1_{Gl}}\right) \end{matrix}\right] \\ +\rho_{T^{E}}T^{E}\left[\begin{matrix} \left(\frac{c_{Gl}}{1+c_{Gl}}\right)\left(\frac{IFN_{\gamma Gl}}{1+IFN_{\gamma Gl}}\right)\left(\frac{IL12_{Gl}}{1+IL12_{Gl}}\right) \\ \left(\frac{IL2_{Gl}}{1+IL2_{Gl}}\right)\left(\frac{K_{T}}{K_{T}+PD1_{Gl}}\right) \end{matrix}\right]-\left(\frac{k_{Treg}T^{E}_{Gl}Treg_{Gl}}{(1+T^{E}_{Gl})(1+Treg_{Gl})}\right) \\ -\pi_{T^{M}}T^{E}_{Gl}V_{Gl}-d_{T^{E}}T^{E}_{Gl}V_{Gl} \end{matrix}\right)/V_{Gl}$ | (89) |
| --- | --- |

- 1. Memory CD8^+^ T cells (*T^M^*)

| $\frac{dT^{M}_{Gl}}{dt}= \left(\begin{matrix} -Q_{_{Gl-b}} T^{M}_{Gl}+Q_{Tl-Gl} T^{M}_{Tl} \\ +\pi_{T^{M}}T^{E}_{Gl}V_{Gl}-d_{T^{M}}T^{M}_{Gl}V_{Gl} \end{matrix}\right)/V_{Gl}$ | (90) |
| --- | --- |

- 1. Regulatory T cells (*Treg*)

| $\frac{dTreg_{Gl}}{dt}= \left(\begin{matrix} -Q_{_{Gl-b}} Treg_{Gl}+Q_{Tl-Gl} Treg_{Tl} \\ +S_{Treg}+\rho_{Treg}Treg_{Gl}\left(\frac{a_{Gl}}{1+c_{Gl}}\right)\left(\frac{IL12_{Gl}}{1+IL12_{Gl}}\right)+\rho_{Treg,TNFa}Treg_{Gl}TNFa_{Gl} \\ -d_{Treg}Treg_{Gl}V_{Gl} \end{matrix}\right)/V_{b}$ | (91) |
| --- | --- |

- 1. Macrophages

| $\frac{dM_{_{Gl}}}{dt}= \left(\begin{matrix} -Q_{_{Gl-b}} M_{Gl}+Q_{Tl-Gl} M_{Gl} \\ x_{M}c_{Gl}+x_{M,IL6}IL6-x_{c,M}\frac{c_{Gl}}{1+a_{Gl}} \\ -d_{M}M_{Gl}V_{Gl} \end{matrix}\right)/V_{Gl}$ | (92) |
| --- | --- |

- 1. Natural killer cells

| $\frac{dNK_{b}}{dt}= \left(\begin{matrix} -Q_{_{Gl-b}} NK_{Gl}+Q_{Tl-Gl}NK_{Tl} \\ +x_{c,\mathrm{NK}}\frac{c_{Gl}}{1+a_{Gl}}\left(\frac{IL12_{Gl}}{1+IL12_{Gl}}\right) \\ -d_{NK}NK_{b}V_{b} \end{matrix}\right)/V_{b}$ | (93) |
| --- | --- |

- 1. Naïve B cells (*B^N^*)

| $\frac{dB^{N}_{Gl}}{dt}= \left(\begin{matrix} Q_{_{Gl-b}} B^{N}_{Gl}+Q_{Tl-Gl}B^{N}_{Tl} \\ +S_{B}-h_{T}B^{N}_{Gl}DC^{*}_{Gl}Th^{E}_{Gl}\left[\begin{matrix} w_{Ag-B}^{i=1-34}\left(\frac{Ag_{Gl}}{1+Ag_{Gl}}\right)\left(\frac{c_{Gl}}{1+c_{Gl}}\right)\left(\frac{IFN_{\gamma Gl}}{1+IFN_{\gamma Gl}}\right) \\ \left(\frac{IL12_{Gl}}{1+IL12_{Gl}}\right)\left(\frac{K_{T}}{K_{T}+PD1_{Gl}}\right) \end{matrix}\right] \\ -d_{B^{N}}B^{N}_{Gl}V_{Gl} \end{matrix}\right)/V_{Gl}$ | (94) |
| --- | --- |

- 1. Activated B cells (*B^N^*)

| $\frac{dB^{A}_{Gl}}{dt}= \left(\begin{matrix} -Q_{_{Gl-b}} B^{A}_{Gl}+Q_{Tl-Gl}B^{A}_{Tl} \\ +h_{T}B^{N}_{Gl}DC^{*}_{Gl}Th^{E}_{Gl}\left[\begin{matrix} w_{Ag-B}^{i=1-34}\left(\frac{Ag_{Gl}}{1+Ag_{Gl}}\right)\left(\frac{c_{Gl}}{1+c_{Gl}}\right)\left(\frac{IFN_{\gamma Gl}}{1+IFN_{\gamma Gl}}\right) \\ \left(\frac{IL12_{Gl}}{1+IL12_{Gl}}\right)\left(\frac{K_{T}}{K_{T}+PD1_{Gl}}\right) \end{matrix}\right] \\ +\rho_{B}Th^{E}_{Gl}B^{A}_{Gl}-\pi_{s}B^{A}_{Gl}-\pi_{L}(Th^{E}_{Gl}+Th^{M}_{Gl})B^{A}_{Gl} \\ -d_{B^{A}}B^{A}_{Gl}V_{Gl} \end{matrix}\right)/V_{Gl}$ | (95) |
| --- | --- |

- 1. Long-lived plasma (antibody-secreting) B cells (*P^L^*)

| $\frac{dP^{L}_{Gl}}{dt}= \left(\begin{matrix} -Q_{_{Gl-b}}P^{L}_{Gl}+Q_{Tl-Gl}P^{L}_{Tl} \\ +\pi_{L}(Th^{E}_{Gl}+Th^{M}_{Gl})B^{A}_{Gl} \\ -d_{P^{L}}P^{L}_{Gl}V_{Gl} \end{matrix}\right)/V_{Gl}$ | (96) |
| --- | --- |

- 1. Short-lived plasma (antibody-secreting) B cells (*P^S^*)

| $\frac{dP^{S}_{Gl}}{dt}= \left(\begin{matrix} -Q_{_{Gl-b}}P^{S}_{Gl}+Q_{Tl-Gl}P^{S}_{Tl} \\ +\pi_{s}B^{A}_{Gl} \\ -d_{P^{S}}P^{S}_{Gl}V_{Gl} \end{matrix}\right)/V_{Gl}$ | (97) |
| --- | --- |

1. Tumor draining lymph nodes
   1. Pro-inflammatory cytokines

|  | (98) |
| --- | --- |

- 1. Anti-inflammatory cytokines

| $\frac{da_{Tl}}{dt}= \left(\begin{matrix} Q_{_{T-Tl}} a_{T}-Q_{Tl-Gl} a_{Tl} \\ + k_{\mathrm{a},N} N_{Tl}(\mathrm{M}_{Tl}+\mathrm{DC}_{Tl}) V_{Tl}+ k_{\mathrm{a},Treg} (Treg) V_{Tl} \\ + k_{\mathrm{a},\mathrm{M}} (M_{Tl}) V_{Tl}-d_{a} a_{Tl}V_{Tl} \end{matrix}\right)/V_{Tl}$ | (99) |
| --- | --- |

- 1. Antibody

| $\frac{dA_{Tl}}{dt}= \left(\begin{matrix} Q_{_{T-Tl}} A_{T}+Q_{Tl-Gl} A_{Tl} \\ + \pi_{AS}P^{S}_{Tl}+ \pi_{AL}P^{L}_{Tl}-d_{a}A_{Tl} \end{matrix}\right)/V_{Tl}$ | (100) |
| --- | --- |

- 1. Antigen

|  | (101) |
| --- | --- |

- 1. The reaction rate of the IL-6 reads

| $\frac{dIL6_{Tl}}{dt}= \left(\begin{matrix} Q_{_{T-Tl}} IL6_{T}-Q_{Tl-Gl} IL6_{Tl} \\ + k_{\mathrm{IL}6,NK} (NK_{Tl}) V_{Tl}+ k_{\mathrm{IL}6,\mathrm{M}} (M_{Tl}) V_{Tl} \\ + k_{\mathrm{IL}6,\mathrm{T}} (\mathrm{Th}^{E}_{Tl}+\mathrm{T}^{E}_{Tl}) V_{Tl}+ k_{\mathrm{IL}6,DC} (DC^{*}_{Tl}) V_{Tl} \\ - k_{IL6} IL6 _{Tl}V_{Tl} \end{matrix}\right)/V_{Tl}$ | (102) |
| --- | --- |

- 1. The reaction rate of the IL-2 reads

| $\frac{dIL2_{Tl}}{dt}= \left(\begin{matrix} Q_{_{T-Tl}} IL2_{T}-Q_{Tl-Gl} IL2_{Tl} \\ + k_{\mathrm{IL}2,\mathrm{M}} (M_{Tl}) V_{Tl} \\ + k_{\mathrm{IL}2,\mathrm{T}} (\mathrm{Th}^{E}_{Tl}+\mathrm{T}^{E}_{Tl}) V+ k_{\mathrm{IL}2,DC} (DC^{*}_{Tl}) V_{Tl} \\ - k_{IL2} IL2_{Tl}V_{Tl} \end{matrix}\right)/V_{Tl}$ | (103) |
| --- | --- |

- 1. The reaction rate of the IL-12 reads

| $\frac{dIL12_{Tl}}{dt}= \left(\begin{matrix} Q_{_{T-Tl}} IL12_{Tl}-Q_{Tl-Gl} IL12_{Tl} \\ + k_{\mathrm{IL}12,\mathrm{N}} (N_{Tl}) V_{Tl} \\ + k_{\mathrm{IL}12,M} (M_{Tl}) V_{Tl}+ k_{\mathrm{IL}12,DC} (DC^{*}_{Tl}) V_{Tl} \\ - k_{IL12} IL12_{Tl}V_{Tl} \end{matrix}\right)/V_{Tl}$ | (104) |
| --- | --- |

- 1. The reaction rate of the VEGF reads

|  | (105) |
| --- | --- |

- 1. Interferon gamma ($\boldsymbol{IF}\boldsymbol{N}_{\boldsymbol{\gamma}}$)

| $\frac{dIFN\gamma_{Tl}}{dt}= \left(\begin{matrix} Q_{_{T-Tl}} IFN\gamma_{T}-Q_{Tl-GL} IFN\gamma_{Tl} \\ + k_{IFN\gamma,NK} (NK_{Tl}) V_{Tl}+ k_{IFN\gamma,\mathrm{Th}} (\mathrm{Th}^{\mathrm{E}}_{Tl}) V_{Tl} \\ + k_{IFN\gamma,\mathrm{T}} (T^{E}_{Tl}) V_{Tl}- k_{IFN\gamma} IFN\gamma_{Tl}V_{Tl} \end{matrix}\right)/V_{Tl}$ | (106) |
| --- | --- |

- 1. Interferon gamma ($\boldsymbol{TNF}_{\boldsymbol{a}}$)

| $\frac{dTNFa_{Tl}}{dt}= \left(\begin{matrix} Q_{_{T-Tl}} TNFa_{T}-Q_{Tl-Gl} TNFa_{Tl} \\ + k_{TNFa,NK} (NK_{Tl}) V_{Tl}+ k_{TNFa,\mathrm{Th}} (\mathrm{Th}^{\mathrm{E}}_{Tl}) V_{Tl} \\ + k_{TNFa,\mathrm{T}} (T^{E}_{Tl}) V_{Tl}+ k_{TNFa,\mathrm{M}} (M_{Tl}) V_{Tl} \\ - k_{TNFa} TNFa_{Tl}V_{Tl} \end{matrix}\right)/V_{Tl}$ | (107) |
| --- | --- |

- 1. Neutrophils

| $\frac{dN_{Tl}}{dt}= \left(\begin{matrix} Q_{_{T-Tl}} N_{T}-Q_{Tl-Gl} N_{Tl} \\ + k_{\mathrm{N}} \frac{c_{Tl}}{1+a_{Tl}}+ k_{\mathrm{N},\mathrm{IL}6} (IL6_{Tl}) V_{Tl} \\ - k_{N} N_{Tl}V_{Tl} \end{matrix}\right)/V_{Tl}$ | (108) |
| --- | --- |

- 1. Neutrophils Extracellular Traps (NETs)

| $\frac{dNETs_{Tl}}{dt}= \left(\begin{matrix} Q_{_{T-Tl}} NETs_{T}-Q_{Tl-Gl} NETs_{Tl} \\ + k_{N}N_{Tl}V_{Tl}-d_{NETs}NETs_{Tl}V_{Tl} \end{matrix}\right)/V_{Tl}$ | (109) |
| --- | --- |

- 1. Immature dendritic cells (DC)

| $\frac{dDC_{Tl}}{dt}= \left(\begin{matrix} Q_{_{T-Tl}} DC_{T}-Q_{Tl-Gl} DC_{Tl} \\ +S_{DC}-[x_{DC}\frac{c_{Tl}}{1+a_{Tl}}DC_{Tl}+k_{Ag,DC}DC_{Tl}Ag_{Tl}]V_{Tl} \\ -k_{Ag,Terg}Treg_{Tl}DC_{Tl}V_{Tl}-d_{DC}DC_{Tl}V_{Tl} \end{matrix}\right)/V_{Tl}$ | (110) |
| --- | --- |

- 1. Antigen presenting cells APCs (DC^*^)

| $\frac{dDC^{*}_{Tl}}{dt}= \left(\begin{matrix} Q_{T-Tl} DC^{*}_{T}-Q_{Tl-Gl} DC^{*}_{Tl} \\ +S_{DC^{*}}+[x_{DC}\frac{c_{Tl}}{1+a_{Tl}}DC_{Tl}+k_{Ag,DC}DC_{Tl}Ag_{Tl}]V_{Tl} \\ -k_{Ag,Terg}Treg_{Gl}DC^{*}_{Tl}V_{Tl}-d_{DC}DC^{*}_{Tl}V_{Tl} \end{matrix}\right)/V_{Tl}$ | (111) |
| --- | --- |

- 1. Naïve CD4^+^ T cells (*Th^N^*)

| $\frac{dTh^{N}_{Tl}}{dt}= \left(\begin{matrix} Q_{_{T-Tl}} Th^{N}_{Tl}-Q_{Tl-Gl} Th^{N}_{Tl} \\ -h_{Th}DC^{*}_{Tl}Th^{N}_{Tl}\left[\begin{matrix} w_{Ag-CD4}^{i=1-34}\left(\frac{Ag_{Tl}}{1+Ag_{Tl}}\right)\left(\frac{c_{Tl}}{1+c_{Tl}}\right)\left(\frac{IFN_{\gamma Tl}}{1+IFN_{\gamma Tl}}\right) \\ \left(\frac{IL12_{Tl}}{1+IL12_{Tl}}\right)\left(\frac{IL2_{Tl}}{1+IL2_{Tl}}\right)\left(\frac{K_{T}}{K_{T}+PD1_{Tl}}\right) \end{matrix}\right] \\ -\left(\frac{h_{Treg}DC_{Tl}Th^{N}_{Tl}a_{Tl}}{(K_{Th,DC}+DC_{Tl})(K_{DC,Th}+Th^{N}_{Tl})(K_{Treg,a}+a_{Tl})}\right)-d_{Th^{N}}Th^{N}_{Tl}V_{Tl} \end{matrix}\right)/V_{Tl}$ | (112) |
| --- | --- |

- 1. Effector CD4^+^ T cells (*Th^E^*)

| $\frac{dTh^{E}_{Tl}}{dt}= \left(\begin{matrix} Q_{_{T-Tl}} Th^{E}_{T}-Q_{Tl-Gl} Th^{E}_{Tl} \\ +h_{Th}DC^{*}_{Tl}Th^{N}_{Tl}\left[\begin{matrix} w_{Ag-CD4}^{i=1-34}\left(\frac{Ag_{Tl}}{1+Ag_{Tl}}\right)\left(\frac{c_{Tl}}{1+c_{Tl}}\right)\left(\frac{IFN_{\gamma Tl}}{1+IFN_{\gamma Tl}}\right) \\ \left(\frac{IL12_{Tl}}{1+IL12_{Tl}}\right)\left(\frac{IL2_{Tl}}{1+IL2_{Tl}}\right)\left(\frac{K_{T}}{K_{T}+PD1_{Tl}}\right) \end{matrix}\right] \\ +\rho_{Th^{E}}Th^{E}_{Tl}\left[\begin{matrix} \left(\frac{c_{Tl}}{1+c_{Tl}}\right)\left(\frac{IFN_{\gamma Tl}}{1+IFN_{\gamma Tl}}\right)\left(\frac{IL12_{Tl}}{1+IL12_{Tl}}\right) \\ \left(\frac{IL2_{Tl}}{1+IL2_{Tl}}\right)\left(\frac{K_{T}}{K_{T}+PD1_{Tl}}\right) \end{matrix}\right] \\ -\left(\frac{k_{Treg}Th^{E}_{Tl}Treg_{Tl}}{(1+Th^{E}_{Tl})(1+Treg_{Tl})}\right)-\pi_{Th^{M}}Th^{E}_{Tl}V_{Tl}-d_{Th^{E}}Th^{E}_{Tl}V_{Tl} \end{matrix}\right)/V_{Tl}$ | (113) |
| --- | --- |

- 1. Memory CD4^+^ T cells (*Th^M^*)

| $\frac{dTh^{M}_{Tl}}{dt}= \left(\begin{matrix} Q_{_{T-Tl}} Th^{M}_{T}-Q_{Tl-Gl} Th^{M}_{Tl} \\ +\pi_{Th^{M}}Th^{E}_{Tl}V_{Tl} \\ -d_{Th^{M}}Th^{M}_{Tl}V_{Tl} \end{matrix}\right)/V_{Tl}$ | (114) |
| --- | --- |

- 1. Naïve CD8^+^ T cells (*T^N^*)

| $\frac{dT^{N}_{Tl}}{dt}= \left(\begin{matrix} Q_{_{T-Tl}} T^{N}_{T}-Q_{Tl-Gl} T^{N}_{Tl} \\ -h_{T}DC^{*}T^{N}_{Tl}\left[\begin{matrix} w_{Ag-CD8}^{i=1-34}\left(\frac{Ag_{Tl}}{1+Ag_{Tl}}\right)\left(\frac{c_{Tl}}{1+c_{Tl}}\right)\left(\frac{IFN_{\gamma Tl}}{1+IFN_{\gamma Tl}}\right) \\ \left(\frac{IL12_{Tl}}{1+IL12_{Tl}}\right)\left(\frac{IL2_{Tl}}{1+IL2_{Tl}}\right)\left(\frac{K_{T}}{K_{T}+PD1_{Tl}}\right) \end{matrix}\right] \\ -d_{T^{N}}T^{N}_{Tl}V_{Tl} \end{matrix}\right)/V_{Tl}$ | (115) |
| --- | --- |

- 1. Effector CD8^+^ T cells (*T^E^*)

| $\frac{dT^{E}_{Tl}}{dt}= \left(\begin{matrix} Q_{_{T-Tl}} T^{E}_{T}-Q_{Tl-Gl} T^{E}_{Tl} \\ +h_{T}DC^{*}_{Tl}T^{N}_{Tl}\left[\begin{matrix} w_{Ag-CD8}^{i=1-34}\left(\frac{Ag_{Tl}}{1+Ag_{Tl}}\right)\left(\frac{c_{Tl}}{1+c_{Tl}}\right)\left(\frac{IFN_{\gamma Tl}}{1+IFN_{\gamma Tl}}\right) \\ \left(\frac{IL12_{Tl}}{1+IL12_{Tl}}\right)\left(\frac{IL2_{Tl}}{1+IL2_{Tl}}\right)\left(\frac{K_{T}}{K_{T}+PD1_{Tl}}\right) \end{matrix}\right] \\ +\rho_{T^{E}}T^{E}_{Tl}\left[\begin{matrix} \left(\frac{c_{Tl}}{1+c_{Tl}}\right)\left(\frac{IFN_{\gamma Tl}}{1+IFN_{\gamma Tl}}\right)\left(\frac{IL12_{Tl}}{1+IL12_{_{Tl}}}\right) \\ \left(\frac{IL2_{Tl}}{1+IL2_{Tl}}\right)\left(\frac{K_{T}}{K_{T}+PD1_{Tl}}\right) \end{matrix}\right]-\left(\frac{k_{Treg}T^{E}_{Tl}Treg_{Tl}}{(1+T^{E}_{Tl})(1+Treg_{Tl})}\right) \\ -\pi_{T^{M}}T^{E}_{Tl}V_{Tl}-d_{T^{E}}T^{E}_{Tl}V_{Tl} \end{matrix}\right)/V_{Tl}$ | (116) |
| --- | --- |

- 1. Memory CD8^+^ T cells (*T^M^*)

| $\frac{dT^{M}_{Tl}}{dt}= \left(\begin{matrix} Q_{_{T-Tl}} T^{M}_{T}-Q_{Tl-Gl} T^{M}_{Tl} \\ +\pi_{T^{M}}T^{E}_{Tl}V_{Tl}-d_{T^{M}}T^{M}_{Tl}V_{Tl} \end{matrix}\right)/V_{Tl}$ | (117) |
| --- | --- |

- 1. Regulatory T cells (*Treg*)

| $\frac{dTreg_{Tl}}{dt}= \left(\begin{matrix} Q_{_{T-Tl}} Treg_{T}-Q_{Tl-Gl} Treg_{Tl} \\ +S_{Treg}+\rho_{Treg}Treg_{Tl}\left(\frac{a_{Tl}}{1+c_{Tl}}\right)\left(\frac{IL12_{Tl}}{1+IL12_{Tl}}\right)+\rho_{Treg,TNFa}Treg_{Tl}TNFa_{Tl} \\ -d_{Treg}Treg_{Tl}V_{Tl} \end{matrix}\right)/V_{Tl}$ | (118) |
| --- | --- |

- 1. Macrophages

| $\frac{dM_{Tl}}{dt}= \left(\begin{matrix} Q_{_{T-Tl}} M_{T}-Q_{Tl-Gl} M_{Gl} \\ x_{M}c_{Tl}+x_{M,IL6}IL6_{Tl}-x_{c,M}\frac{c_{Tl}}{1+a_{Tl}} \\ -d_{M}M_{Tl}V_{Tl} \end{matrix}\right)/V_{Tl}$ | (119) |
| --- | --- |

- 1. Natural killer cells

| $\frac{dNK_{Tl}}{dt}= \left(\begin{matrix} Q_{_{T-Tl}} NK_{T}-Q_{Tl-Gl}NK_{Tl} \\ +x_{c,\mathrm{NK}}\frac{c_{Tl}}{1+a_{Tl}}\left(\frac{IL12_{Tl}}{1+IL12_{Tl}}\right) \\ -d_{NK}NK_{Tl}V_{Tl} \end{matrix}\right)/V_{Tl}$ | (120) |
| --- | --- |

- 1. Naïve B cells (*B^N^*)

| $\frac{dB^{N}_{Tl}}{dt}= \left(\begin{matrix} Q_{_{T-Tl}} B^{N}_{T}-Q_{Tl-Gl}B^{N}_{Tl} \\ +S_{B}-h_{T}B^{N}_{Tl}DC^{*}_{Tl}Th^{E}_{Tl}\left[\begin{matrix} w_{Ag-B}^{i=1-34}\left(\frac{Ag_{Tl}}{1+Ag_{Tl}}\right)\left(\frac{c_{Tl}}{1+c_{Tl}}\right)\left(\frac{IFN_{\gamma Tl}}{1+IFN_{\gamma Tl}}\right) \\ \left(\frac{IL12_{Tl}}{1+IL12_{Tl}}\right)\left(\frac{K_{T}}{K_{T}+PD1_{Tl}}\right) \end{matrix}\right] \\ -d_{B^{N}}B^{N}_{Tl}V_{Tl} \end{matrix}\right)/V_{Tl}$ | (121) |
| --- | --- |

- 1. Activated B cells (*B^N^*)

| $\frac{dB^{A}_{Tl}}{dt}= \left(\begin{matrix} Q_{_{T-Tl}} B^{A}_{T}-Q_{Tl-Gl}B^{A}_{Tl} \\ +h_{T}B^{N}_{Tl}DC^{*}_{Tl}Th^{E}_{Tl}\left[\begin{matrix} w_{Ag-B}^{i=1-34}\left(\frac{Ag_{Tl}}{1+Ag_{Tl}}\right)\left(\frac{c_{Tl}}{1+c_{Tl}}\right)\left(\frac{IFN_{\gamma Tl}}{1+IFN_{\gamma Tl}}\right) \\ \left(\frac{IL12_{Tl}}{1+IL12_{Tl}}\right)\left(\frac{K_{T}}{K_{T}+PD1_{Tl}}\right) \end{matrix}\right] \\ +\rho_{B}Th^{E}_{Tl}B^{A}_{Tl}-\pi_{s}B^{A}_{Tl}-\pi_{L}(Th^{E}_{Tl}+Th^{M}_{Tl})B^{A}_{Tl} \\ -d_{B^{A}}B^{A}_{Tl}V_{Tl} \end{matrix}\right)/V_{Tl}$ | (122) |
| --- | --- |

- 1. Long-lived plasma (antibody-secreting) B cells (*P^L^*)

| $\frac{dP^{L}_{Tl}}{dt}= \left(\begin{matrix} Q_{_{T-Tl}}P^{L}_{T}-Q_{Tl-Gl}P^{L}_{Tl} \\ +\pi_{L}(Th^{E}_{Tl}+Th^{M}_{Tl})B^{A}_{Tl} \\ -d_{P^{L}}P^{L}_{Tl}V_{Tl} \end{matrix}\right)/V_{Tl}$ | (123) |
| --- | --- |

- 1. Short-lived plasma (antibody-secreting) B cells (*P^S^*)

| $\frac{dP^{S}_{Tl}}{dt}= \left(\begin{matrix} Q_{_{T-Tl}}P^{S}_{T}-Q_{Tl-Gl}P^{S}_{Tl} \\ +\pi_{s}B^{A}_{Tl} \\ -d_{P^{S}}P^{S}_{Tl}V_{Tl} \end{matrix}\right)/V_{Tl}$ | (124) |
| --- | --- |

**Table A**. Values of model parameters.

| Parameter | Description | Value [Units] | Reference |
| --- | --- | --- | --- |
| $\boldsymbol{S}_{\boldsymbol{n}}$, $\boldsymbol{K}_{\boldsymbol{g}}$, $\boldsymbol{\chi}_{\boldsymbol{n}}$ | Cytokine production by innate immune cells and infected cells.  Production of anti-inflammatory cytokines by macrophages and neutrophils.  Production rate of neutrophils by pro-inflammatory cytokines. | 2.1x10^-2^ [pg/h] | Smith A.M. et al [6]  Dunster J.L. et al. [7] |
| $\boldsymbol{d}_{\boldsymbol{c}}$ | Degradation rate of pro-inflammatory cytokines | 8.3x10^-1^ [1/h] | Smith A.M. et al [6] |
| $\boldsymbol{\varphi}_{\boldsymbol{a}}$ | Production of anti-inflammatory cytokines by macrophages interaction neutrophils | 2.1x10^-6^ [ml] | Dunster J.L. et al. [7] |
| $\boldsymbol{\gamma}_{\boldsymbol{a}}$ | Degradation rate of anti-inflammatory cytokines | 3 [1/day] | Dunster J.L. et al. [7] |
| $\boldsymbol{R}_{\boldsymbol{H}}$ | Rate of production of healthy epithelial cells | 2.75x10^-3^ [1/h] | Mahasa KJ et al.[8] |
| $\boldsymbol{\gamma}_{\boldsymbol{n}}$ , $\boldsymbol{\gamma}_{\boldsymbol{Ma}}$, $\boldsymbol{\gamma}_{\boldsymbol{IL}\boldsymbol{6}}$ | Rate of production of neutrophil NETs.  Degradation rate of macrophages.  Degradation rate of IL6 | 6.3x10^-4^ [1/h] | Dunster J.L. et al. [7]  Voutouri C. et al. [9, 10] |
| $\boldsymbol{\gamma}_{\boldsymbol{NETS}}$ | Degradation rate of NETs. | 6.3x10^-7^[1/h] | Voutouri C. et al. [9, 10] |
| $\boldsymbol{\chi}_{\boldsymbol{m}}$ | Production rate of macrophages by pro-inflammatory cytokines | 0.02 [1/pg/h] | Dunster J.L. et al. [7] |
| $\boldsymbol{K}_{\boldsymbol{IL}\boldsymbol{6}}$ | Production rate of IL6 by activated macrophages | 0.5 [ml/h] | Voutouri C. et al. [9, 10] |
| $\boldsymbol{K}_{\boldsymbol{IL}\boldsymbol{6}}^{\boldsymbol{on}}$ | Rate of IL6 binding to IL6 receptors | 15 [ml/h/nmol] | Mok W. et al. [11] |
| $\boldsymbol{K}_{\boldsymbol{IL}\boldsymbol{6}}^{\boldsymbol{off}}$ | Rate of IL6 detachment from IL6 receptors | 5.22[1/h] | Mok W. et al. [11] |
| $\boldsymbol{h}_{\boldsymbol{IL}\boldsymbol{6}\boldsymbol{R}}$ | The half-life for IL6 receptor | 1.5 [min] | Voutouri C. et al. [9, 10] |
| $\boldsymbol{S}_{\boldsymbol{sIL}\boldsymbol{6}\boldsymbol{R}}$,  $\boldsymbol{S}_{\boldsymbol{IL}\boldsymbol{6}\boldsymbol{R}}$*,* $\boldsymbol{S}_{\boldsymbol{sACE}\boldsymbol{2}}$ | Source production of sIL6R.  Source production of IL6R receptor,  Source production of sACE2 | 0.57[fmol/ml/h] | Voutouri C. et al. [9, 10] |
| $\boldsymbol{K}_{\boldsymbol{sIL}\boldsymbol{6}\boldsymbol{R}}^{\boldsymbol{on}}$ | Rate of IL6 binding to soluble IL6 receptors | 17 [ml/h/nmol] | Mok W. et al. [11] |
| $\boldsymbol{S}_{\boldsymbol{VEGF}}$ | Source production of VEGF | 0.235[fmol/ml/h] | Voutouri C. et al. [9, 10] |
| $\boldsymbol{K}_{\boldsymbol{VEGF}}$,  $\boldsymbol{K}_{\boldsymbol{sIL}\boldsymbol{6}\boldsymbol{R}}$ | Production rate of VEGF by IL6 bound on the soluble IL6 receptor.  Conversion rate of IL6 receptor to soluble IL6 receptor. | 5.22[1/h] | Mok W. et al. [11] |
| $\boldsymbol{K}_{\boldsymbol{sIL}\boldsymbol{6}\boldsymbol{R}}^{\boldsymbol{off}}$ | Rate of IL6 detachment from soluble IL6 receptors | 2.22[1/h] | Mok W. et al. [11] |
| $\boldsymbol{K}_{\boldsymbol{sACE}\boldsymbol{2}}$ | Rate of soluble ACE2 receptor binding to the virus | 17 [ml/h/nmol] | Mok W. et al. [11] |
| $\boldsymbol{\chi}_{\boldsymbol{N-IL}\boldsymbol{6}}\boldsymbol{,}$  $\boldsymbol{\chi}_{\boldsymbol{Ma-IL}\boldsymbol{6}}$ | Production of neutrophils by IL6-R activation,  Production of macrophages by IL6-R activation | 5.26[1/fmol/h] | Dunster J.L. et al. [7] |
| $\boldsymbol{K}_{\boldsymbol{ec}}$ | Proliferation of endothelial cells | 5.22x10^-7^ [1/h] | Voutouri C. et al. [9, 10] |
| $\boldsymbol{S}_{\boldsymbol{v}}^{\boldsymbol{0}}$ | Vascular density | 70 [1/cm] | Mpekris F. et al [12] |
| $\boldsymbol{V}_{\boldsymbol{O}\boldsymbol{2}}^{\boldsymbol{max}}$ | oxygen uptake rate | 3700 [mlO_2_/min] | Weibel E.R. et al [13] |
| $\boldsymbol{P}_{\boldsymbol{A}}$ | Partial pressure of oxygen in alveolar air | 100 [mmHg] | T.K. Roy, T.W. Secomb [14] |
| $\boldsymbol{K}_{\boldsymbol{02}}^{\boldsymbol{0}}$ | Krogh permeability coefficient KO2 | 3.3x10^-8^ [cm^2^/min/mmHg] | Weibel E.R. et al [13] |
| $\boldsymbol{S(A)}$ | Alveolar gas exchange areas | 130 [m^2^] | Weibel E.R. et al [13] |
| $\boldsymbol{S(c)}$ | Capillary gas exchange areas | 115 [m^2^] | Weibel E.R. et al [13] |
| $\boldsymbol{\tau}_{\boldsymbol{hb}}$ | Harmonic mean thickness of the air–blood barrier | 1 [μm] | Weibel E.R. et al [13] |
| $\boldsymbol{\theta}_{\boldsymbol{O}\boldsymbol{2}}$ | Oxygen unloading conductance of blood | 1.8[mlO_2_/ml/min/mmHg] | Roy T.K., Secomb T.W. [14] |
| $\boldsymbol{V(c)}$ | Lung blood volume | 194 [ml] | Roy T.K., Secomb T.W. [14] |
| $\boldsymbol{n}$ | Hill coefficient | 2.7 | Roy T.K., Secomb T.W. [14] |
| $\boldsymbol{P}_{\boldsymbol{50}}$ | Oxygen tension when the binding sites are 50 percent saturated. | 26.3 [mmHg] | Roy T.K., Secomb T.W. [14] |
| $\boldsymbol{K}_{\boldsymbol{IL}\boldsymbol{6-TN,TE}}$ | Production rate of IL6 by Naïve T cells, Activated T cells | 0.02554[mol/pg/s] | Lai, X., & Friedman A. [15] |
| $\boldsymbol{\gamma}_{\boldsymbol{VEGF}}$ | Production rate of VEGF by hypoxia | 0.0152[mol/ml/h] | Voutouri C. et al. [9, 10] |
| $\boldsymbol{K}_{\boldsymbol{c-IL}\boldsymbol{6}}$ | Production of pro-inflammatory cytokines by IL6-R activation | 0.03 [ml/h/fmol] | Voutouri C. et al. [9, 10] |
| $\boldsymbol{K}_{\boldsymbol{c-EC}}$ | Production of pro-inflammatory cytokines by healthy endothelial cells | 1 [pg] | Voutouri C. et al. [9, 10] |
| $\boldsymbol{h}_{\boldsymbol{T}}\boldsymbol{=}\boldsymbol{h}_{\boldsymbol{TE}}$=$\boldsymbol{h}_{\boldsymbol{ThE}}\mathbf{=}\boldsymbol{h}_{\boldsymbol{BE}}$ | Conversion rate of Naïve T and B cells to activated cells | 0.00254 [1/h] | Lai, X., & Friedman A. [15] |
| $\boldsymbol{As}$ | Antigen strength | 1 | Lai, X., & Friedman A. [15] |
| $\boldsymbol{K}_{\boldsymbol{T}}$ | Constant for blocking PD-1 inhibition | 1.365x10^-18^ [g/cm^3^] | Lai, X., & Friedman A. [15] |
| $\boldsymbol{\varepsilon}$ | Degradation of activated T cells by PD1 bound to PDL1 | 0.01575 [cm^3^/g/s] | Lai, X., & Friedman A. [15] |
| $\boldsymbol{d}_{\boldsymbol{TN,N,Ma}}\boldsymbol{,}$  $\boldsymbol{d}_{\boldsymbol{H,EC}}$ | Degradation rate PD1 by Naïve T cells, neutrophils, macrophages,  Degradation rate PDL1 by healthy epithelial, endothelial cells | 0.00215 [1/s] | Lai, X., & Friedman A. [15] |
| a_PL_ | Association of PD-1 with PD-L1 | 0.258[mm^3/g/s] | Lai, X., & Friedman A. [15] |
| $\boldsymbol{d}_{\boldsymbol{Q}}$ | Dissociation rate of PD-L1 from PD-1 | 0.1 [1/d] | Lai, X., & Friedman A. [15] |
| γ_A_ | Source of anti-PD1 | 1x10^-10^[g/cm^3/d] | Lai, X., & Friedman A. [15] |
| µ_PD1 ,_ $\boldsymbol{d}_{\boldsymbol{TE}}$ | Efficiency of PD1 blocking by ICI,  Degradation rate PD1 by Activated T cells | 0.00215x10^-3^[1/s] | Lai, X., & Friedman A. [15] |
| $\boldsymbol{d}_{\boldsymbol{A}}$ | Degradation rate of anti-PD1 | 0.0462 [1/d] | Lai, X., & Friedman A. [15] |
| $\boldsymbol{\lambda}_{\boldsymbol{H,EC}}$  $\boldsymbol{\lambda}_{\boldsymbol{TE,TN,N,Ma}}$  $\boldsymbol{h}_{\boldsymbol{PDL}\boldsymbol{1}}$ | Production rate of PDL1 by healthy epithelial, endothelial cells,  Production rate of PD1 by activated T cells, Naïve T cells, neutrophils, macrophages,  Production rate of the PD1 ligand by Effector (Activated) T | 0.154 [1/s] | Lai, X., & Friedman A. [15] |
| $\boldsymbol{a}_{\boldsymbol{PL}}$ | rate of PD1 binding to PDL1 | 0.258 [mm^3^/g/s] | Lai, X., & Friedman A. [15] |
| $\boldsymbol{\gamma}_{\boldsymbol{A}}$ | Source term of anti-PD1 | 1x10^-10^ [g/cm^3^/d] | Lai, X., & Friedman A. [15] |
| $\boldsymbol{\mu}_{\boldsymbol{A}}$ | Degradation rate of anti-PD1 by PD1 | 6.87x10^6^ [cm^3^/g/d] | Lai, X., & Friedman A. [15] |
| $Q_{lower body}$ | Blood flow rate of lower body | 413 [ml/min] | Voutouri C. et al. [9, 10] |
| $Q_{uppe body}$ | Blood flow rate of upper body | 138 [ml/min] | Voutouri C. et al. [9, 10] |
| $L_{lower body}$ | Lymphatic flow rate of lower body | 1x 10^-3^ [ml/min] | Voutouri C. et al. [9, 10] |
| $L_{uppe body}$ | Lymphatic flow rate of upper body | 2.6 x 10^-2^ [ml/min] | Voutouri C. et al. [9, 10] |
| $\boldsymbol{L}_{\boldsymbol{H}}$ | Lymphatic flow rate of healthy tissues | 4.3x 10^-2^ [ml/min] | Voutouri C. et al. [9, 10] |
| $\boldsymbol{L}_{\boldsymbol{T}}$ | Lymphatic flow rate of tumor part | 2x 10^-2^ [ml/min] | Voutouri C. et al. [9, 10] |
| $V_{lower body}$ | Averaged vascular volume of lower body | 700 [ml] | Voutouri C. et al. [9, 10] |
| $V_{uppe body}$ | Averaged vascular volume of upper body | 150 [ml] | Voutouri C. et al. [9, 10] |
| $\boldsymbol{V}_{\boldsymbol{H}}$ | Averaged vascular volume of healthy tissues | 99.9 [ml] | Voutouri C. et al. [9, 10] |
| $\boldsymbol{V}_{\boldsymbol{T}}$ | Averaged vascular volume of tumor part | 50 [ml] | Voutouri C. et al. [9, 10] |
| $mt'_{i}$ | Maximum production of micro-thrombus (IL6) | 7x 10^-3^ [mM/min] | Voutouri C. et al. [9, 10] |
| $k_{a}$ | Natural decay rate of antibody | 4x10^-7^ [1/d ] | Voutouri C. et al. [9, 10] |
| $k_{c,innate}$ | Production rate of the cytokine by innate immune cells | 2.3x10^-9^ [ml/h/nmol] | Voutouri C. et al. [9, 10] |
| $K_{c-IL6}$ | Production rate of the cytokine by IL6 bound to sIL6R | 0.03 [ml/h/fmol] | Voutouri C. et al. [9, 10] |
| $d_{s}$ | Degradation rate of pro-inflammatory cytokines | 8.3x10^-1^ [1/h] | Smith A.M. et al [6] |
| $K_{F}$ | Killing rate of infected cells by NK cells | 4.2 [1/d] | A. Haghnegahdar et all [16] |
| $S_{DC}$ | source term of dendritic cells | 1 [1/d] | Lee et al [1] |
| $\beta_{DC}$ | Conversion rate of immature dendritic cells to APC | 1x10^-2^ [1/d/M] | Lee et al [1] |
| $d_{DC}$ | Death rate of dendritic cells | 1x10^-3^ [1/d] | Lee et al [1] |
| $S_{ThN}$ | source term of Naïve CD4+ T cells | 4x10^-4^ [1/d] | Lee et al [1] |
| $d_{ThN}$ $d_{TN}$ | Death rate of Naïve CD4+ T cells  Death rate of Naïve CD8+ T cells | 0.75 [1/d] | Lee et al [1] |
| $\boldsymbol{\pi}_{\boldsymbol{ThM}}$ | conversion rate of effector CD4+ T cells to memory CD4+ T cells | - | Voutouri C. et al. [9, 10] |
| $S_{TN}$ | source term of Naïve CD8+ T cells | 750 [1/d] | Lee et al [1] |
| $\boldsymbol{\pi}_{\boldsymbol{TM}}$ | conversion rate of effector CD8+ T cells to memory CD8+ T cells | - | Voutouri C. et al. [9, 10] |
| $S_{B}$ | source term of Naïve B cells | 2 [1/d] | Lee et al [1] |
| $d_{B}$ | Death rate of Naïve B cells | 0.002 [1/d] | Lee et al [1] |
| $\boldsymbol{\pi}_{\boldsymbol{s}}$ | differentiation rate of activated B cells into short-lived antibody-secreting plasma cells. | 1e-3 [1/d] | Lee et al [1] |
| $\boldsymbol{\pi}_{\boldsymbol{L}}$ | differentiation rate of activated B cells into long-lived antibody-secreting plasma cells. | 8e-9 [1/d] | Lee et al [1] |
| $\boldsymbol{\rho}_{\boldsymbol{B}}$ | the proliferation rate of activated B cells | 2.6 [1/d] | Lee et al [1] |
| $\boldsymbol{\delta}_{\boldsymbol{L}}$ | death rate of long-lived antibody-secreting plasma cell | 3e-2 [1/d] | Lee et al [1] |
| $\boldsymbol{\delta}_{\boldsymbol{s}}$ | death rate of short-lived antibody-secreting plasma cell | 0.1 [1/d] | Lee et al [1] |
| $\boldsymbol{\delta}_{\boldsymbol{ThM, TM}}$ | death rate of memory CD4+ cells, CD8+ cells | 3e-4 [1/d] | Voutouri C. et al. [9, 10] |
| $\boldsymbol{S}^{\boldsymbol{Va}}$ | Source term for Free vaccine | 1[M/d] | Voutouri C. et al. [9, 10] |
| $\boldsymbol{K}_{\boldsymbol{cell}\mathbf{-}\boldsymbol{Vaccine}}^{\boldsymbol{on}}$ | Binding of the vaccine particles to cells | 17 [ml/h] | Voutouri C. et al. [9, 10] |
| $\boldsymbol{K}_{\boldsymbol{cell}\mathbf{-}\boldsymbol{Vaccine}}^{\boldsymbol{off}}$ | Unbinding of the vaccine particles to cells | 5.22[m^3^/h] | Voutouri C. et al. [9, 10] |
| $\boldsymbol{K}_{\boldsymbol{dVa}}$ | Degradation rate of vaccine particles | 8.1e-4 [1/d] | Voutouri C. et al. [9, 10] |
| $\boldsymbol{K}_{\boldsymbol{intVa}}$ | internalization of the bound vaccine particles into the cells | 5.78e-2[1/s] | Voutouri C. et al. [9, 10] |
| $\boldsymbol{K}_{\boldsymbol{dVab}}$ | degradation of the bound vaccine particles | 4e-4 [1/d] | Voutouri C. et al. [9, 10] |
| $\boldsymbol{K}_{\boldsymbol{Tran}}$ | DNA transcription to mRNA of the internalize vaccine particles | 6.21 e-4 [1/s] | Voutouri C. et al. [9, 10] |
| $\boldsymbol{K}_{\boldsymbol{protein}}$ | production of antigen from the translation of the mRNA | 8.52 e-5 [1/s] | Voutouri C. et al. [9, 10] |
| $\boldsymbol{d}_{\boldsymbol{Vaint}}$ | constant degradation of internalized vaccine | 3.4 e-4 [mole/m^3^/s] | Voutouri C. et al. [9, 10] |
| $\boldsymbol{d}_{\boldsymbol{VaTran}}$ | constant degradation of translation DNA | 1.5 e-4 [mole/m^3^/s] | Voutouri C. et al. [9, 10] |
| $\boldsymbol{d}_{\boldsymbol{Vprotein}}$ | constant degradation of viral proteins | 6.5 e-5 [mole/m^3^/s] | Voutouri C. et al. [9, 10] |
| $\boldsymbol{K}_{\boldsymbol{cell}\mathbf{-}\boldsymbol{Vaccine}}^{\boldsymbol{on}}$ | Binding of the vaccine particles to healthy cells | Baseline:17[ml/h] | Voutouri C. et al. [9, 10] |
| $\boldsymbol{K}_{\boldsymbol{cell}\mathbf{-}\boldsymbol{Vaccine}}^{\boldsymbol{off}}$ | unbinding of the vaccine particles to healthy cells | 6.22[m^3^/h] | Voutouri C. et al. [9, 10] |
| $\boldsymbol{K}_{\boldsymbol{dVa}}$ | Degradation rate of vaccine particles | 9.4e-4 [1/d] | Voutouri C. et al. [9, 10] |
| $\boldsymbol{K}_{\boldsymbol{intVa}}$ | internalization of the bound vaccine particles into the cells | 4.78e-2[1/s] | Voutouri C. et al. [9, 10] |
| $\boldsymbol{K}_{\boldsymbol{dVab}}$ | degradation of the bound vaccine particles | 4.5e-4 [1/d] | Voutouri C. et al. [9, 10] |
| $\boldsymbol{K}_{\boldsymbol{protein}}$ | production of viral protein or viral antigen from the translation of the mRNA | 8.0e-5 [1/s] | Voutouri C. et al. [9, 10] |
| $\boldsymbol{d}_{\boldsymbol{Vaint}}$ | constant degradation of internalized vaccine | 3.8 e-4 [mole/m^3^/s] | Voutouri C. et al. [9, 10] |
| $\boldsymbol{d}_{\boldsymbol{VaTran}}$ | constant degradation of translation DNA | 2 e-4 [mole/m^3^/s] | Voutouri C. et al. [9, 10] |
| $\boldsymbol{d}_{\boldsymbol{Vprotein}}$ | constant degradation of viral proteins | 7e-5 [mole/m^3^/s] | Voutouri C. et al. [9, 10] |

**Table B**. Contribution Scores.

| Component | Program-1 Score | Program-2 Score |
| --- | --- | --- |
| IL2 in Tumor | 0.15 | 0 |
| Treg in TDLN | 0.12 | 0 |
| TNF-Î± in Tumor | 0.13 | 0 |
| Neutrophils in Tumor | 0.1 | 0 |
| Naive CD8+ T in Tumor | 0.08 | 0 |
| Activated CD8+ T in Tumor | 0.09 | 0 |
| TNF-Î± in LN | 0.07 | 0 |
| Activated B in TDLN | 0.06 | 0 |
| NETs in Tumor | 0.05 | 0 |
| Long-lived Plasma B in TDLN | 0.05 | 0 |
| Naive CD4+ T in Tumor | 0 | 0.12 |
| Short-lived Plasma B in Tumor | 0 | 0.11 |
| NK in LN | 0 | 0.09 |
| Naive CD4+ T in LN | 0 | 0.08 |
| Activated CD4+ T in Tumor | 0 | 0.07 |
| Naive CD4+ T in TDLN | 0 | 0.06 |
| Activated CD8+ T in TDLN | 0 | 0.06 |
| IL2 in TDLN | 0 | 0.05 |
| Antigen in TDLN | 0 | 0.04 |
| Treg in Tumor | 0 | 0.03 |

**References**

1. Lee HY, Topham DJ, Park SY, Hollenbaugh J, Treanor J, Mosmann TR, et al. Simulation and prediction of the adaptive immune response to influenza A virus infection. J Virol. 2009;83(14):7151-65. doi: 10.1128/JVI.00098-09. PubMed PMID: 19439465; PubMed Central PMCID: PMCPMC2704765.

2. Li Y, Tenchov R, Smoot J, Liu C, Watkins S, Zhou Q. A Comprehensive Review of the Global Efforts on COVID-19 Vaccine Development. ACS Cent Sci. 2021;7(4):512-33. doi: 10.1021/acscentsci.1c00120. PubMed PMID: 34056083; PubMed Central PMCID: PMCPMC8029445.

3. Dong Y, Dai T, Wei Y, Zhang L, Zheng M, Zhou F. A systematic review of SARS-CoV-2 vaccine candidates. Signal Transduct Target Ther. 2020;5(1):237. doi: 10.1038/s41392-020-00352-y. PubMed PMID: 33051445; PubMed Central PMCID: PMCPMC7551521.

4. Callaway E. The race for coronavirus vaccines: a graphical guide. Nature. 2020;580(7805):576-7. doi: 10.1038/d41586-020-01221-y. PubMed PMID: 32346146.

5. Pushparajah D, Jimenez S, Wong S, Alattas H, Nafissi N, Slavcev RA. Advances in gene-based vaccine platforms to address the COVID-19 pandemic. Adv Drug Deliv Rev. 2021;170:113-41. doi: 10.1016/j.addr.2021.01.003. PubMed PMID: 33422546; PubMed Central PMCID: PMCPMC7789827.

6. Smith AM, McCullers JA, Adler FR. Mathematical model of a three-stage innate immune response to a pneumococcal lung infection. J Theor Biol. 2011;276(1):106-16. doi: 10.1016/j.jtbi.2011.01.052. PubMed PMID: 21300073; PubMed Central PMCID: PMCPMC3066295.

7. Dunster JL, Byrne HM, King JR. The resolution of inflammation: a mathematical model of neutrophil and macrophage interactions. Bull Math Biol. 2014;76(8):1953-80. doi: 10.1007/s11538-014-9987-x. PubMed PMID: 25053556.

8. Mahasa KJ, Eladdadi A, de Pillis L, Ouifki R. Oncolytic potency and reduced virus tumor-specificity in oncolytic virotherapy. A mathematical modelling approach. PLoS One. 2017;12(9):e0184347. doi: 10.1371/journal.pone.0184347. PubMed PMID: 28934210; PubMed Central PMCID: PMCPMC5608221.

9. Voutouri C, Hardin CC, Naranbhai V, Nikmaneshi MR, Khandekar MJ, Gainor JF, et al. Dynamic heterogeneity in COVID-19: Insights from a mathematical model. PloS one. 2024;19(5):e0301780. doi: 10.1371/journal.pone.0301780. PubMed PMID: 38820409; PubMed Central PMCID: PMCPMC11142552 Bristol-Myers Squibb, Genentech/Roche, Ariad/Takeda, Loxo/Lilly, Blueprint, Oncorus, Regeneron, Gilead, Moderna, Mirati, AstraZeneca, Pfizer, Novartis, iTeos, Nuvalent, Karyopharm, Beigene, Silverback Therapeutics, Merck, and GlydeBio; research support from Novartis, Genentech/Roche, and Ariad/Takeda; institutional research support from Bristol-Myers Squibb, Tesaro, Moderna, Blueprint, Jounce, Array Biopharma, Merck, Adaptimmune, Novartis, and Alexo; and has an immediate family member who is an employee with equity at Ironwood Pharmaceuticals. LLM owns equity in Bayer AG and is a consultant for SimBiosys. RKJ received consultant fees from Elpis, Innocoll, SPARC, SynDevRx; owns equity in Accurius, Enlight, Ophthotech, SynDevRx; and serves on the Boards of Trustees of Tekla Healthcare Investors, Tekla Life Sciences Investors, Tekla Healthcare Opportunities Fund, Tekla World Healthcare Fund; and received a grant from Boehringer Ingelheim. Neither any reagent nor any funding from these organizations was used in this study. Other co-authors have no conflict of interests to declare. This does not alter our adherence to PLOS ONE policies on sharing data and materials.

10. Voutouri C, Hardin CC, Naranbhai V, Nikmaneshi MR, Khandekar MJ, Gainor JF, et al. Mechanistic model for booster doses effectiveness in healthy, cancer, and immunosuppressed patients infected with SARS-CoV-2. Proceedings of the National Academy of Sciences. 2023;120(3):e2211132120.

11. Mok W, Stylianopoulos T, Boucher Y, Jain RK. Mathematical modeling of herpes simplex virus distribution in solid tumors: implications for cancer gene therapy. Clinical cancer research : an official journal of the American Association for Cancer Research. 2009;15(7):2352-60. doi: 10.1158/1078-0432.CCR-08-2082. PubMed PMID: 19318482; PubMed Central PMCID: PMC2872130.

12. Mpekris F, Angeli S, Pirentis AP, Stylianopoulos T. Stress-mediated progression of solid tumors: effect of mechanical stress on tissue oxygenation, cancer cell proliferation, and drug delivery. Biomech Model Mechanobiol. 2015;14(6):1391-402. doi: 10.1007/s10237-015-0682-0. PubMed PMID: 25968141; PubMed Central PMCID: PMCPMC4568293.

13. Weibel ER, Sapoval B, Filoche M. Design of peripheral airways for efficient gas exchange. Respir Physiol Neurobiol. 2005;148(1-2):3-21. doi: 10.1016/j.resp.2005.03.005. PubMed PMID: 15921964.

14. Roy TK, Secomb TW. Theoretical analysis of the determinants of lung oxygen diffusing capacity. J Theor Biol. 2014;351:1-8. doi: 10.1016/j.jtbi.2014.02.009. PubMed PMID: 24560722; PubMed Central PMCID: PMCPMC4070740.

15. Lai X, Friedman A. Combination therapy of cancer with cancer vaccine and immune checkpoint inhibitors: A mathematical model. PLoS One. 2017;12(5):e0178479. doi: 10.1371/journal.pone.0178479. PubMed PMID: 28542574; PubMed Central PMCID: PMCPMC5444846.

16. Haghnegahdar A, Zhao J, Feng Y. Lung Aerosol Dynamics of Airborne Influenza A Virus-Laden Droplets and the Resultant Immune System Responses: An In Silico Study. J Aerosol Sci. 2019;134:34-55. doi: 10.1016/j.jaerosci.2019.04.009. PubMed PMID: 31983771; PubMed Central PMCID: PMCPMC6980466.
